# Diversity and distribution of type A influenza viruses: an updated panorama analysis based on protein sequences

**DOI:** 10.1186/s12985-019-1188-7

**Published:** 2019-06-26

**Authors:** Qingye Zhuang, Suchun Wang, Shuo Liu, Guangyu Hou, Jinping Li, Wenming Jiang, Kaicheng Wang, Cheng Peng, Dong Liu, Aizhen Guo, Jiming Chen

**Affiliations:** 1 The State Key Laboratory of Agricultural Microbiology, College of Veterinary Medicine, Huazhong Agricultural University, 1 Shizishan Street, Wuhan, 430070 China; 2 National Avian Influenza Professional Laboratory, China Animal Health and Epidemiology Center, 369 Nanjing Road, Qingdao, 266032 China; 3 Qingdao YeBio Bioengineering Co., Ltd, 369 Nanjing Road, Qingdao, 266032 China

**Keywords:** Distribution, Diversity, Influenza virus, Nomenclature, Phylogenetics, Protein

## Abstract

**Background:**

Type A influenza viruses (IAVs) cause significant infections in humans and multiple species of animals including pigs, horses, birds, dogs and some marine animals. They are of complicated phylogenetic diversity and distribution, and analysis of their phylogenetic diversity and distribution from a panorama view has not been updated for multiple years.

**Methods:**

139,872 protein sequences of IAVs from GenBank were selected, and they were aligned and phylogenetically analyzed using the software tool MEGA 7.0. Lineages and subordinate lineages were classified according to the topology of the phylogenetic trees and the host, temporal and spatial distribution of the viruses, and designated using a novel universal nomenclature system.

**Results:**

Large phylogenetic trees of the two external viral genes (HA and NA) and six internal genes (PB2, PB1, PA, NP, MP and NS) were constructed, and the diversity and the host, temporal and spatial distribution of these genes were calculated and statistically analyzed. Various features regarding the diversity and distribution of IAVs were confirmed, revised or added through this study, as compared with previous reports. Lineages and subordinate lineages were classified and designated for each of the genes based on the updated panorama views.

**Conclusions:**

The panorama views of phylogenetic diversity and distribution of IAVs and their nomenclature system were updated and assumed to be of significance for studies and communication of IAVs.

**Electronic supplementary material:**

The online version of this article (10.1186/s12985-019-1188-7) contains supplementary material, which is available to authorized users.

## Background

Type A influenza viruses (IAVs) causes frequent infections and occasional pandemics in humans and various animals, including birds, pigs, horses, dogs, bats, and marine mammals [1–9]. The groups of IAVs mainly circulating in birds, humans, pigs, horses and dogs were designated as type A avian influenza viruses (AIVs), human influenza viruses (HuIVs), swine influenza viruses (SIVs), equine influenza viruses (EIVs), and canine influenza viruses (CIVs).

The viral genome hosts eight segments. The fourth and sixth segments contain the viral external genes, HA and NA. The other six segments contain the viral internal genes, PB2, PB1, PA, NP, MP, and NS, respectively. The PB1, MP and NS genes all encode two overlapping proteins, PB1-F2 overlapping with PB1, M2 overlapping with M1, and NS2 overlapping with NS1.

According to the diversity of the viral HA and NA genes, IAVs have been classified into 18 HA subtypes (H1-H18) and 11 NA subtypes (N1-N11) [1–8, 10–13]. Combination of the HA and NA subtypes further formed dozens of subtypes (e.g. H1N1, H2N1, H5N1, H3N2, H5N8, H7N9, and H9N2). Additionally, according to the sequences of each of the viral genes and the host, temporal and spatial distribution of the viruses, multiple lineages and subordinate lineages within various subtypes have been classified [1–6, 10–24].

Phylogenetic diversity and distribution of IAVs have been analyzed in a panorama view 9 years ago [1–5]. Now the analysis needs to be updated because some sequences of the viruses (e.g. A/swine/Quebec/4001/2005, A/mink/Nova Scotia/1055488/2007) in GenBank have been revised, and many novel sequences have been uploaded in GenBank, and some novel subtypes, lineages and subordinate lineages of IAVs have emerged. Meanwhile, the analysis capacity of computers also has increased so greatly that much more sequences can be covered in phylogenetic analysis than several years ago, to better reveal the panorama views.

It remains challenging to invent a rational, concise, universal nomenclature system for all lineages and subordinate lineages of IAVs, although a couple of numeral nomenclature systems have been proposed or used for all or some lineages and subordinate lineages of IAVs. For example, clades 2.3.4.4, 2.3.2.1 and 7.2 have been used for designations of some subordinate lineages of the H5 subtype highly pathogenic avian influenza viruses (HPAIVs) circulating in the Eastern Hemisphere in recent years [18, 25, 26]. This nomenclature can be simplified if some of the numbers are replaced by letters, e.g. clade 2.3.4.4 can be simplified as clade 2c4d which is more convenient for communication.

In this report, thousands of protein sequences of each of the genes of IAVs were aligned, and their phylogenetic trees were constructed. Then lineages and subordinate lineages of the viruses were designated according to the topology of the phylogenetic trees and the host, temporal and spatial distribution of the viruses using a novel universal and concise nomenclature system with the aim to update the panorama views of IAVs.

## Materials and methods

### Download and selection of sequences

Viral proteins sequences were downloaded from the Influenza Virus Resource at the website of https://www.ncbi.nlm.nih.gov/genomes/FLU/Database/nph-select.cgi [27]. As for those internal genes and those subtypes of HA or NA genes with ≥2000 downloaded sequences, only the sequences with all amino acid residues revealed were selected for further analysis. For those internal genes and those subtypes of HA or NA genes with < 2000 downloaded sequences, only the sequences with ≥80% amino acid residues revealed were selected for further analysis.

### Phylogenetic analysis of selected sequences

The selected sequences were aligned by the MUSCLE method using the software package of MEGA 7.0 (https://www.megasoftware.net/) [28, 29]. The phylogenetic relationships among the aligned sequences were calculated with the neighbor-joining method under the Poisson model [28, 30]. Substitution rates among sites were set in Gamma distribution (α = 1.0) and gaps in the sequences were treated in pairwise deletion.

### Annotation of lineages and subordinate lineages

Lineages and subordinate lineages were classified according to topology of the phylogenetic trees, i.e. each lineage or subordinate lineage should be a relatively separated branch of the trees for most cases. Distribution of the viruses in hosts, regions and time was also considered in the classification. Primary lineages were designated with the subtype or gene name followed by a point and a number (e.g. H5.2, N1.1, PA.1, and NP.3), and subordinate lineages were designated using letters and numbers alternately (e.g. H5.2a1 and N1.1b1a) to minimize the characters required for the designation. The numbers or letters in the lineage designations, if possible, were in the order of avian, human, swine, equine, and canine followed by others in terms of hosts, and in the order of the Western Hemisphere followed by the Eastern Hemisphere in geography, and in the order from the past to nowadays in isolation time. Avian, human, swine, equine, and canine lineages were given in blue, green, red, pink, and cyan color, respectively, in phylogenetic trees.

### Selection of representative sequences

Representative sequences were randomly selected with consideration of the distribution in hosts, isolation years, isolation places and genetic diversity, and a representative sequence should share the same or similar host, isolation time, and isolation place with the majority of a group of viruses located together in a phylogenetic tree. Avian, human, swine, equine, and canine representative sequences were showed in red, pink, blue, green, and black solid circles, respectively, in phylogenetic trees.

## Results

### General distribution of protein sequences of IAVs

Up to January 31, 2018, a total of 143,535 protein sequences of IAVs were available in GenBank. After excluding the sequences of unclear background, of manipulated materials, or with sequencing errors, and excluding the redundant ones of identical strain names, and the ones with inadequate sequenced length, 139,872 protein sequences were selected for further analysis.

Of these 139,872 sequences, 79,780 sequences were of the viral six internal genes (10,470 NP, 17494 PA, 16009 PB1, 17,063 PB2, 4194 MP, and 14,550 NS), and 33,066 sequences were of the viral HA gene (11,049 H1, 458 H2, 9178 H3, 1206 H4, 3290 H5, 1654 H6, 1714 H7, 159 H8, 2462 H9, 656 H10, 598 H11, 202 H12, 225 H13, 22 H14, 12 H15, 177 H16, 3 H17, and 2 H18), and 27,026 were of the viral NA gene (9311 N1, 11,753 N2, 866 N3, 331 N4, 406 N5, 1447 N6, 613 N7, 1593 N8, 701 N9, 3 N10, and 2 N11). These data suggested that H1, H3, H5, H9, N1, and N2 were likely the dominant subtypes of IAVs circulating in the world.

### Host distribution of the HA sequences

The hosts of 32,623 of the 33,066 HA sequences were known. Of the 32,623 HA sequences, 13,581 were from birds, 12,460 from humans, 6276 from pigs, 155 from horses, 87 from dogs, 16 from cats, 9 from seals, 7 from tigers, 6 from ferrets, 5 from bats, 5 from minks, 4 from pika, 2 from whales, 1 H1 from cheetah, 1 H3 from donkey, 1 H1 from giant anteater, 1 H5 from leopard, 1 H5 from lion, 1 H2 from muskrat, and 1 H1 from sloth bear (Table 1). These data suggested that birds, humans, pigs are the major hosts of IAVs. Also showed in Table 1, most AIVs belong to H9, H5, H6, H7, H3 or H4 subtypes, and most HuIVs and SIVs belong to H1 or H3 subtypes, and most EIVs belong to H3 or H7 subtypes, and most CIVs belong to H3 subtype.

**Table 1:** Host distribution of the 32,623 HA sequences in HA subtypes

	Birds	Humans	Pigs	Horses	Dogs	Bats	Others^a^
H1	390	6265	4378				8
H2	375	73	2				1
H3	1209	5852	1848	133	85		16
H4	1141		5				2
H5	2921	190	21	1			23
H6	1617		1		1		
H7	1531	68	1	20			3
H8	158						
H9	2399	10	18	1	1		2
H10	644	2	1				2
H11	578		1				
H12	197						
H13	215						2
H14	22						
H15	12						
H16	172						
H17						3	
H18						2	
Total	13,581	12,460	6276	155	87	5	59

^a^: The hosts of others included cats, cheetahs, donkeys, ferrets, giant anteaters, leopards, minks, muskrats, pika, seals, sloth bears, tigers, and whales

### Host distribution of the NA sequences

The hosts of 26,792 of the 27,026 NA sequences were known. Of the 26,792 sequences, 10,599 were from birds, 10,157 from humans, 5717 from pigs, 160 from horses, 109 from dogs, 14 from cats, 9 from minks, 6 from seals, 5 from bats, 4 from ferrets, 1 N8 from camel, 1 N1 cheetah, 1 N8 from donkey, 1 N1 from lion, 1 N6 from muskrat, 6 N1 from tigers, and 1 N9 from whale (Table 2). These data suggested that birds, humans and pigs are the major hosts of IAVs. As showed in Table 2, most AIVs, HuIVs and SIVs belong to N1 or N2 subtypes, and most EIVs belong to N7 or N8 subtypes, and most CIVs belong to N2 or N8 subtypes.

**Table 2:** Host distribution of the 26,792 NA sequences in NA subtypes

	Birds	Humans	Pigs	Horses	Dogs	Bats	Others^a^
N1	2287	4534	2454		12		14
N2	2746	5558	3249	1	47		16
N3	841	2	4				1
N4	326						2
N5	405		1				
N6	1430	3	7				4
N7	585	2	1	8			3
N8	1349	1	1	151	50		4
N9	630	57					1
N10						3	
N11						2	
Total	10,599	10,157	5717	160	109	5	45

^a^: The hosts of others included cats, camels, cheetahs, donkeys, ferrets, lions, minks, muskrats, seals, tigers, and whales

### Temporal distribution of the HA sequences

The temporal distribution of 33,018 of the 33,066 HA sequences were known and given in Table 3, which suggested that the sequences increased more and more rapidly after 1970. Sequences of H1, H3, H5, and H9 were significantly more than those of other subtypes after 2000.

**Table 3:** Temporal distribution of the 33,018 HA sequences of IAVs in HA subtypes

	1900s	1910s	1920s	1930s	1940s	1950s	1960s	1970s	1980s	1990s	2000s	2010s	Total
H1		1		17	17	14	6	131	105	182	3940	6614	11,027
H2						33	39	20	61	57	134	113	457
H3							27	147	169	348	2040	6440	9171
H4						1		52	51	42	651	408	1205
H5						1	5	21	46	66	1708	1438	3285
H6							5	65	67	91	970	456	1654
H7	1		3	3	1	2	5	33	31	208	679	738	1704
H8							2	3	5	3	113	33	159
H9							1	13	26	90	648	1681	2459
H10					1	1	1	8	28	20	358	239	656
H11						2		23	46	66	279	182	598
H12								9	15	3	126	49	202
H13								7	33	16	91	78	225
H14									2			20	22
H15								5	4		1	2	12
H16								4	11	6	54	102	177
H17											2	1	3
H18												2	2
Total	1	1	3	20	19	54	91	541	700	1198	11,794	18,596	33,018

### HA subtype distribution of the NA sequences

Of the 27,026 NA sequences, 26,133 were of known HA subtypes, and the HA-subtype distribution of the 26,133 NA sequences was given in Table 4, which suggested that H1N1, H1N2, H3N2, H5N1, and H9N2 were the predominant subtypes circulating worldwide.

**Table 4:** HA-subtype distribution of the 26,133 NA sequences

Subtype	H1	H2	H3	H4	H5	H6	H7	H8	H9	H10	H11	H12	H13	H14	H15	H16	H17	H18	Total
N1	6921	26	65	8	1715	263	101	1	16	19	19	4							9158
N2	1839	122	7022	89	478	400	143	2	1299	14	71	18	9	1					11,507
N3	39	139	21	22	95	6	297	1	4	78	32	4	2	5		73			818
N4	9	3	2	22	4	11	42	148	2	54		8		1	1				307
N5	11	22	23	17	44	61	4	1	12	25	4	147		2					373
N6	15	5	147	697	121	197	27	1	4	22	10	4	61	4					1315
N7	5	14	4	9	7	3	229	1	6	279	2	3		2					564
N8	19	10	902	147	90	150	20	2	5	51	10	15	12	2	1				1436
N9	20	34	7	11	18	10	253		6	12	258	3	14		4				650
N10																	3		3
N11																		2	2
Total	8878	375	8193	1022	2572	1101	1116	157	1354	554	406	206	98	17	6	73	3	2	26,133

Through analysis of 33,349 protein sequences from GenBank with known HA and NA subtypes, the top five combination subtypes circulating in birds, humans, pigs, horses, and dogs were listed in Table 5, which suggested that most AIVs belong to H5N1 or H9N2 subtypes, and most HuIVs belong to H1N1 or H3N2 subtypes, and most SIVs belong to H1N1, H1N2 or H3N2 subtypes, and most EIVs belong to H3N8 or H7N7 subtypes, and most CIVs belong to H3N8 or H3N2 subtypes. The data suggested that H3 subtype IAVs have adapted to birds, humans, pigs, horses, and dogs.

**Table 5:** Top five combination subtypes of AIVs, HuIVs, SIVs, EIVs, and CIVs with the relevant strain numbers given in parentheses

	First	Second	Third	Fourth	Fifth
AIVs	H5N1 (1931)	H9N2 (1753)	H5N2 (641)	H3N8 (633)	H4N6 (542)
HuIVs	H3N2 (6816)	H1N1 (6328)	H5N1 (215)	H2N2 (80)	H7N9 (59)
SIVs	H1N1 (2375)	H1N2 (1945)	H3N2 (1737)	H3N1 (34)	H5N1 (19)
EIVs	H3N8 (808)	H7N7 (24)	H5N1 (1)	H9N2 (1)	
CIVs	H3N8 (217)	H3N2 (60)	H5N1 (4)	H3N1 (2)	

### Panorama phylogenetic relationships of IAVs

Figures 1, 2, 3, 4, 5, 6, 7, 8, 9, 10, 11, 12, 13, 14, 15, 16 showed the panorama phylogenetic relationships of IAVs based on their viral HA protein sequences, and Figs. 17, 18, 19, 20, 21, 22, 23, 24, 25, 26 showed the panorama phylogenetic relationships of IAVs based on their viral NA protein sequences, with one figure for one or more HA or NA subtypes. Figures 27-32 showed the panorama phylogenetic relationships of IAVs based on the protein sequences of the six internal genes.

**Fig. 1:**
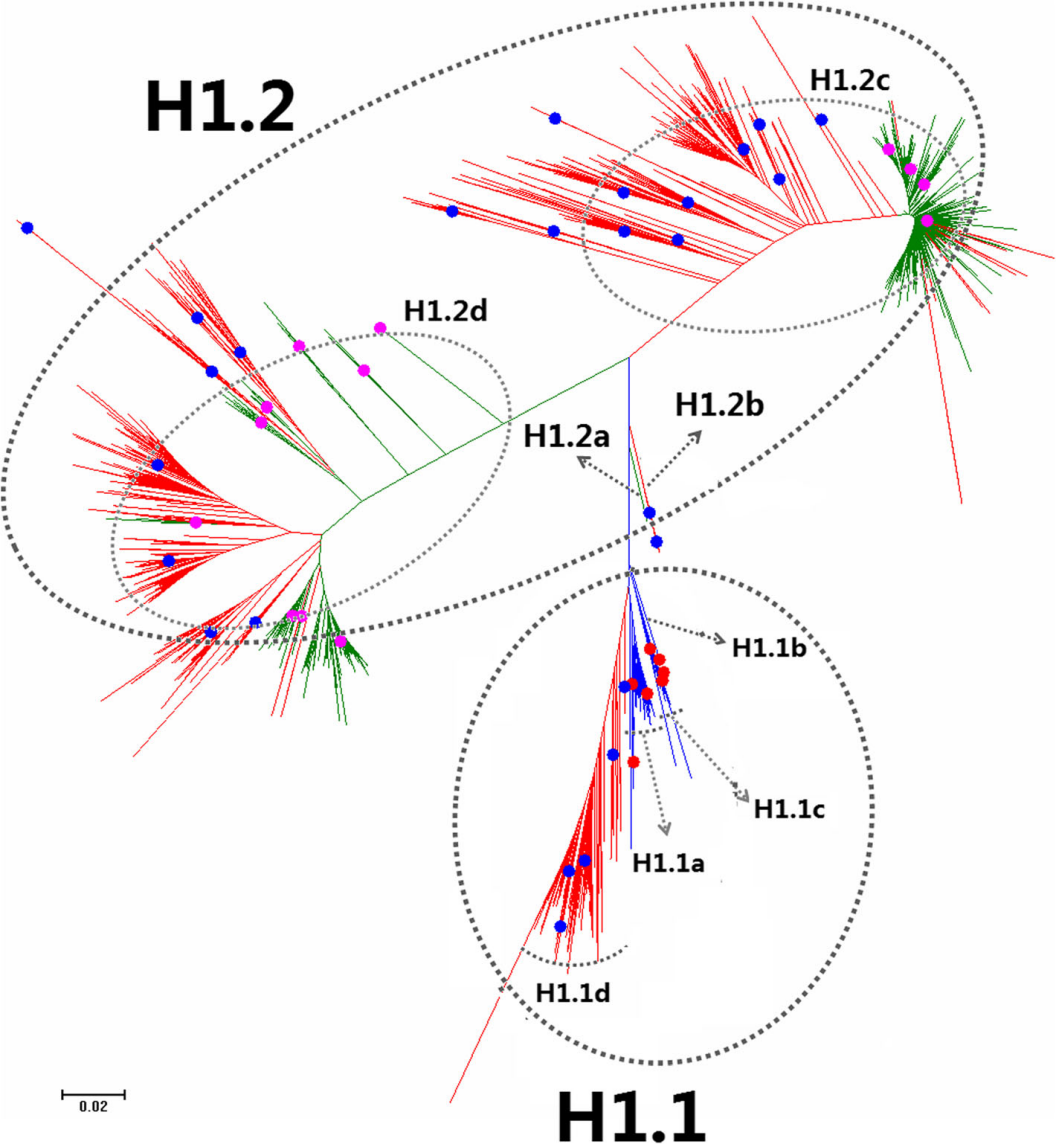
Phylogenetic diversity and distribution of H1 subtypes of IAVs based on HA sequences. H1 subtype IAVs were classified into two primary lineages, H1.1 and H1.2. H1.1 mainly corresponded to H1 subtype AIVs and some SIVs which originated from the AIVs. H1.2 corresponded to classical H1 subtype HuIVs and SIVs

**Fig. 2:**
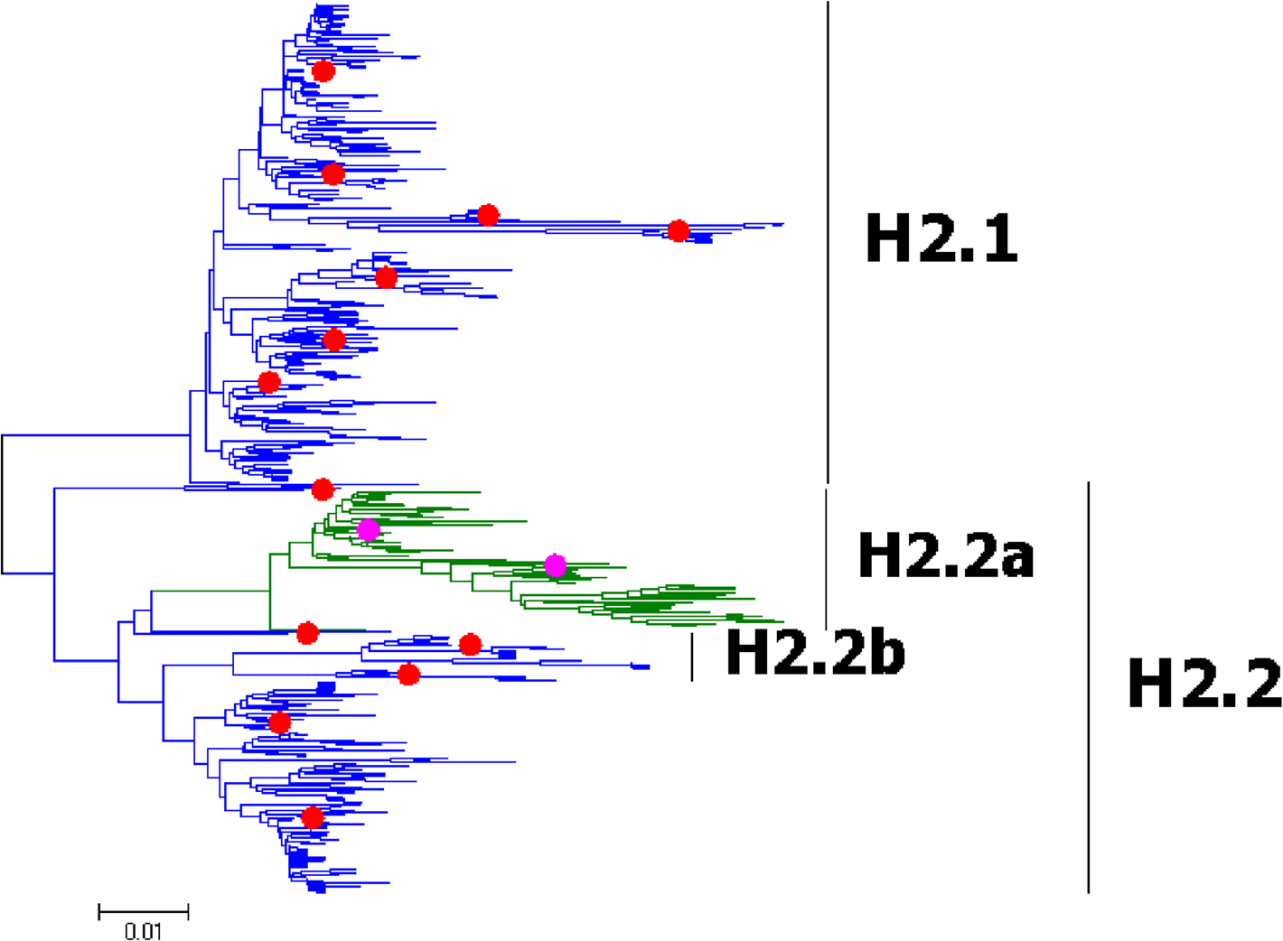
Phylogenetic diversity and distribution of H2 subtypes of IAVs based on HA sequences. H2 subtype IAVs were classified into two primary lineages, largely corresponding to the AIVs isolated from the Western and Eastern Hemispheres, respectively

**Fig. 3:**
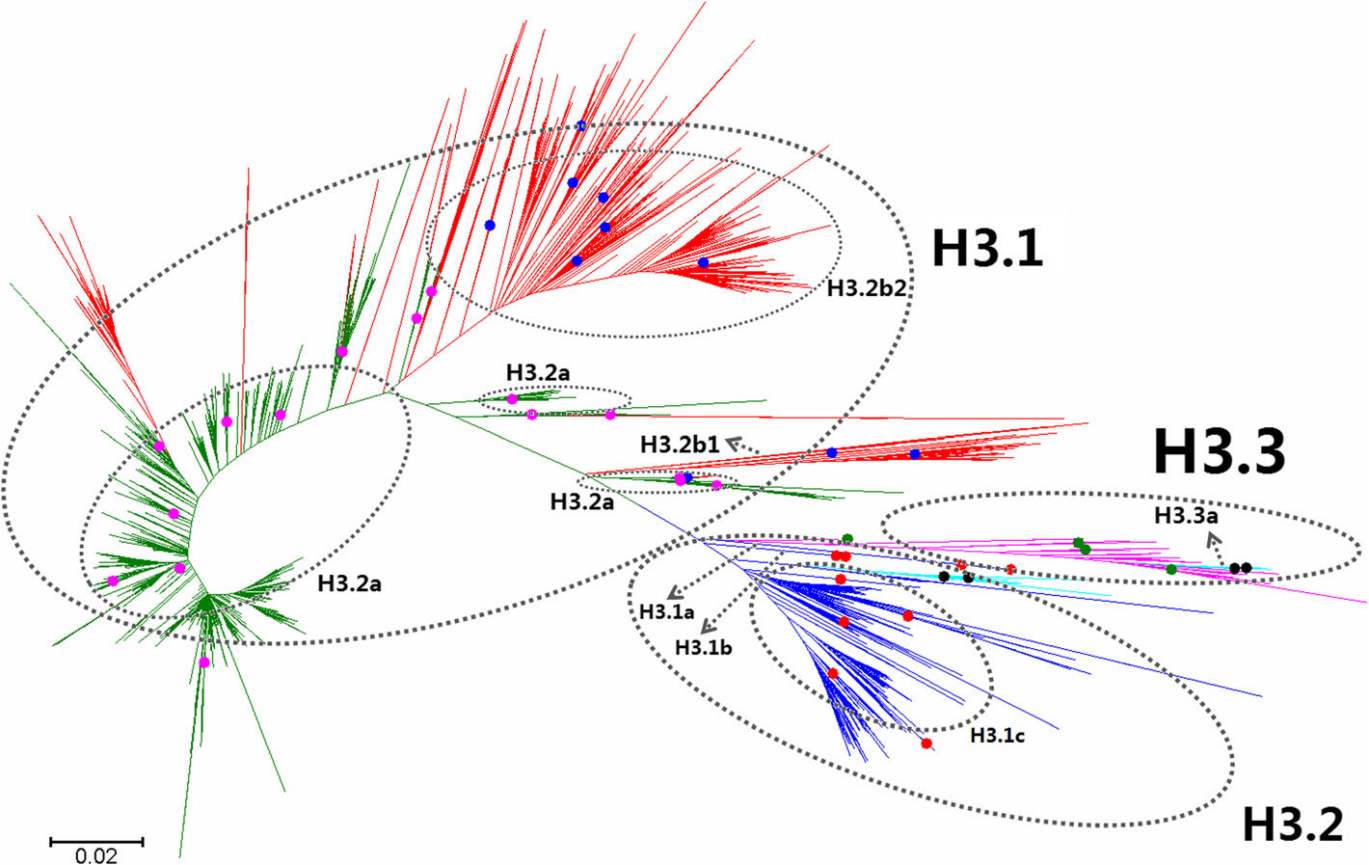
Phylogenetic diversity and distribution of H3 subtypes of IAVs based on HA sequences. H3 subtype IAVs were classified into three primary lineages, H3.1, H3.2 and H3.3. H3.1 corresponded to H3 subtype AIVs, and several secondary lineages within H3.1, H3.1a-H3.1c, were designated. H3.2 corresponded to many HuIVs and SIVs circulating worldwide. H3.3 corresponded to the EIVs circulating from 1963 to nowadays

**Fig. 4:**
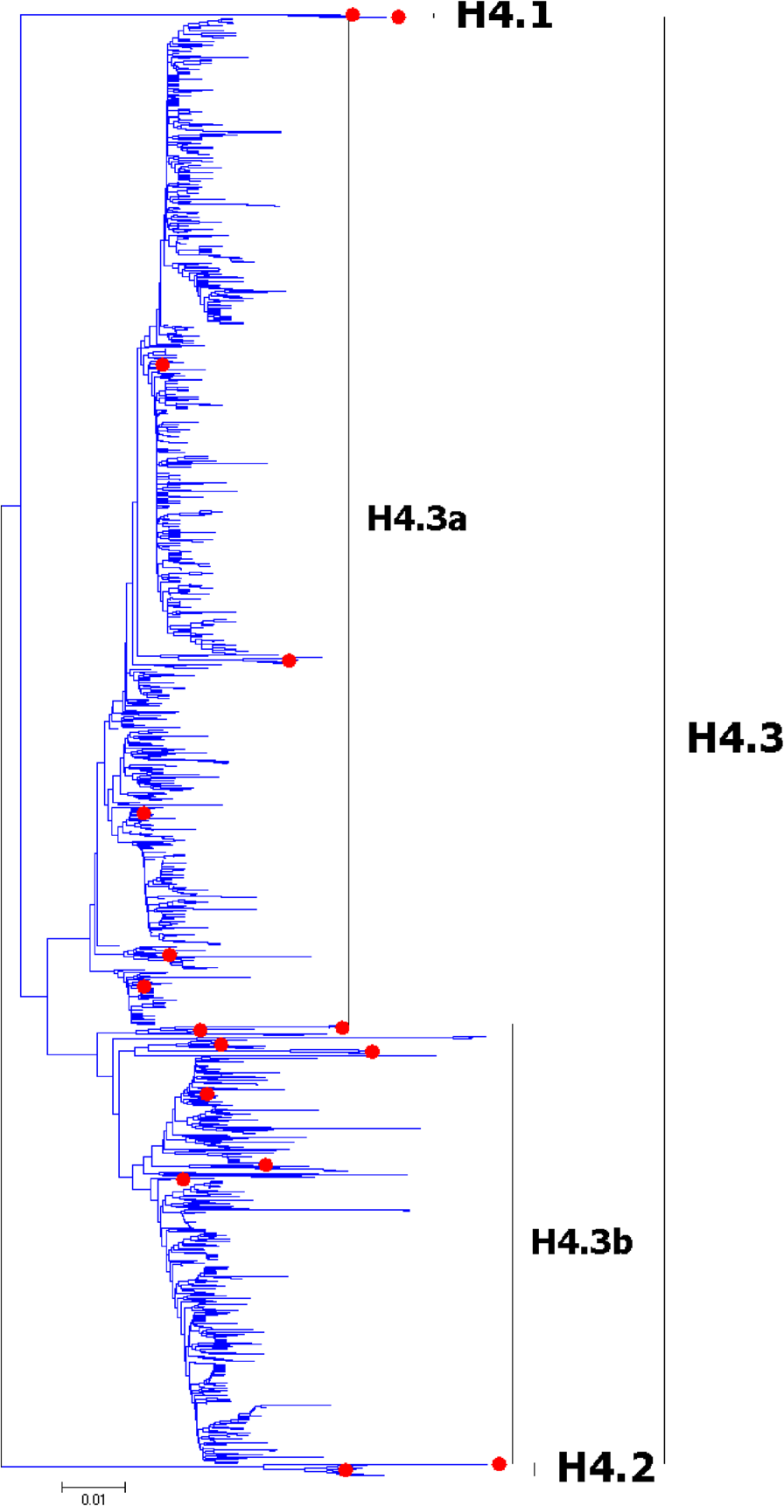
Phylogenetic diversity and distribution of H4 subtypes of IAVs based on HA sequences. H4 subtype IAVs were classified into three primary lineages; H4.1 and H4.2 mainly corresponded to AIVs circulating in the North America; H4.3 corresponded to AIVs from both Hemispheres

**Fig. 5:**
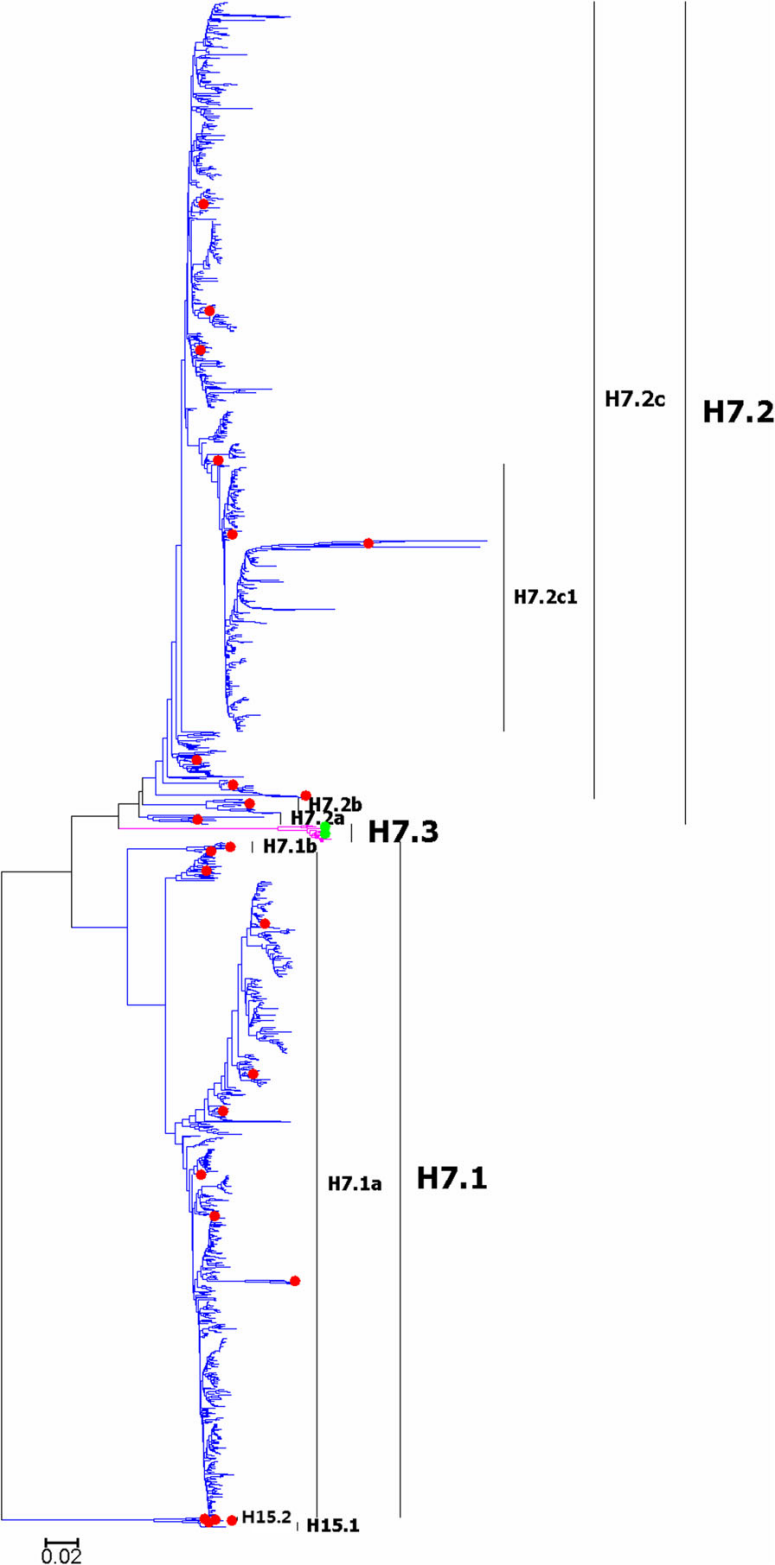
Phylogenetic diversity and distribution of H7 and H15 subtypes of IAVs based on HA sequences. H7 subtype IAVs were classified into three primary lineages, H7.1, H7.2 and H7.3. H7.1 and H7.2 mainly corresponded to H7 subtype AIVs circulating in the Western and Eastern Hemispheres. H7.3 corresponded to H7 subtype EIVs which have not been found after the 1970s. H15 subtype IAVs could be classified into two lineages, H15.1 and H15.2. H15.1 corresponded to AIVs isolated from Australia in the 1970s and the 1980s. H15.2 corresponded to AIVs isolated from Russia around the year 2010

**Fig. 6:**
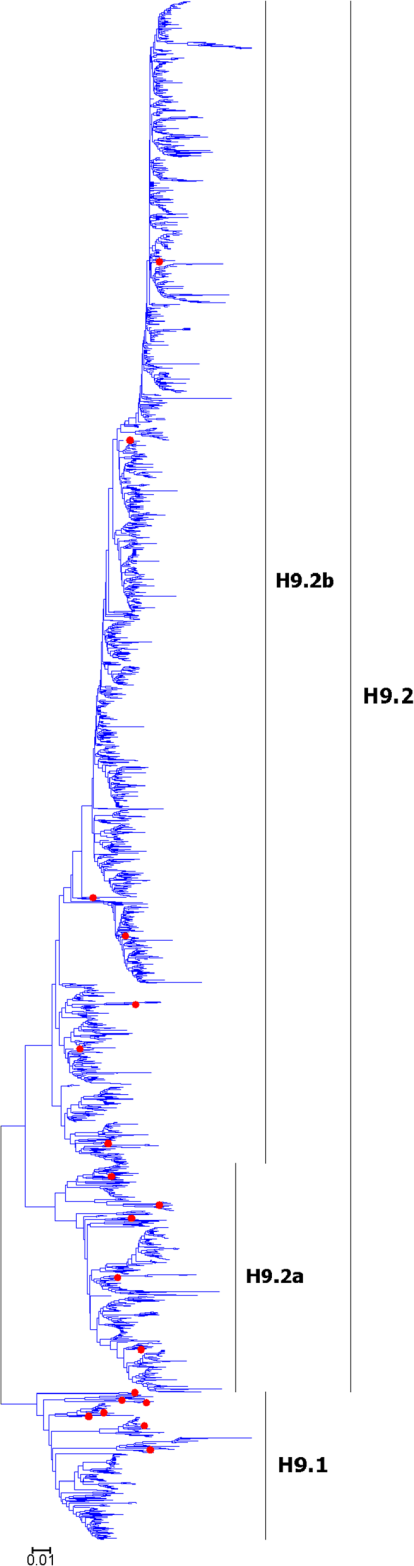
Phylogenetic diversity and distribution of H9 subtypes of IAVs based on HA sequences. H9 subtype IAVs were classified into two primary lineages, H9.1 and H9.2. H9.1 corresponded to some H9 subtype AIVs circulating worldwide. H9.2 corresponded to many AIVs circulating in the Eastern Hemisphere

**Fig. 7:**
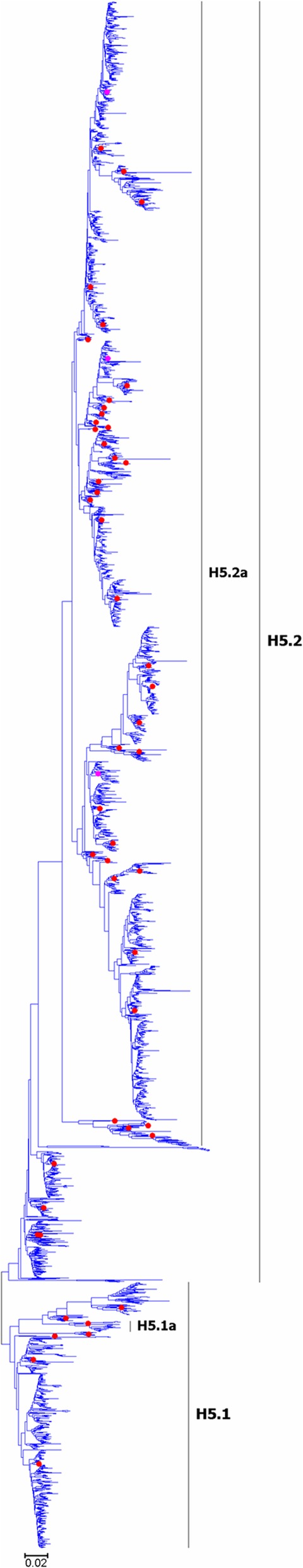
Phylogenetic diversity and distribution of H5 subtypes of IAVs based on HA sequences. H5 subtype IAVs were classified into two primary lineages, which mainly corresponded to AIVs circulating in the Western and Eastern Hemispheres

**Fig. 8:**
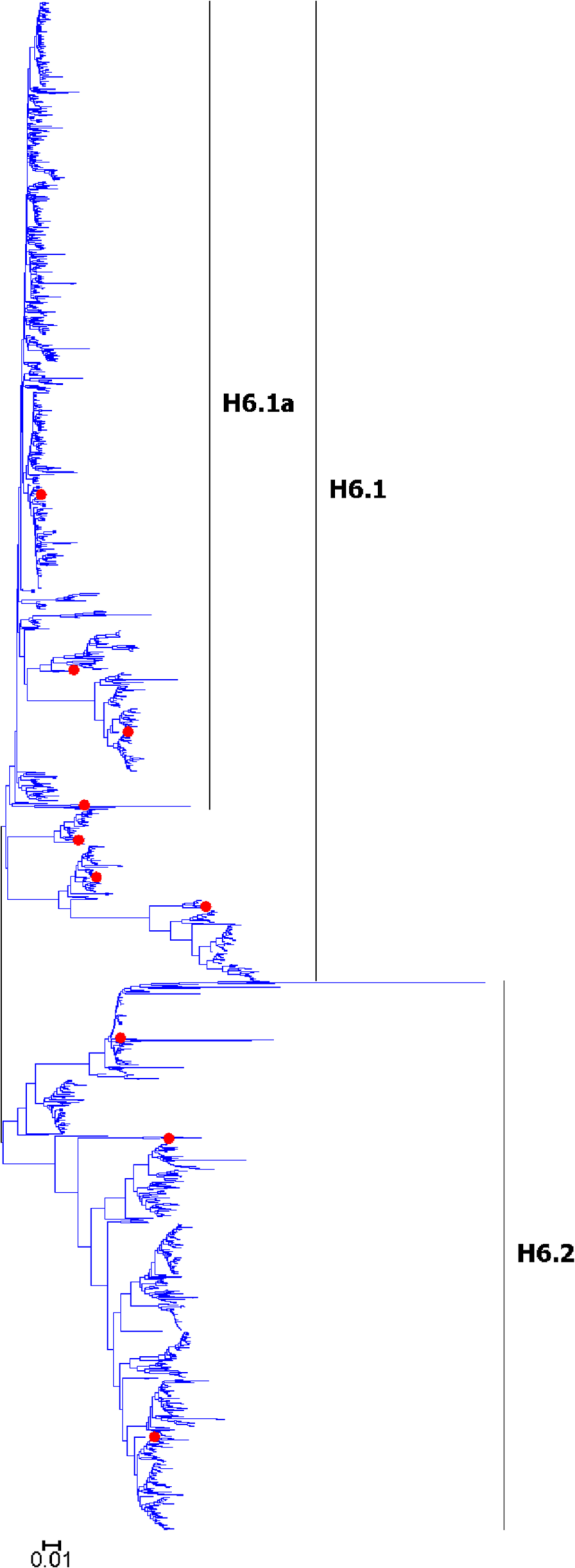
Phylogenetic diversity and distribution of H6 subtypes of IAVs based on HA sequences. H6 subtype IAVs were classified into two primary lineages, which mainly corresponded to AIVs circulating in the Western and Eastern Hemispheres

**Fig. 9:**
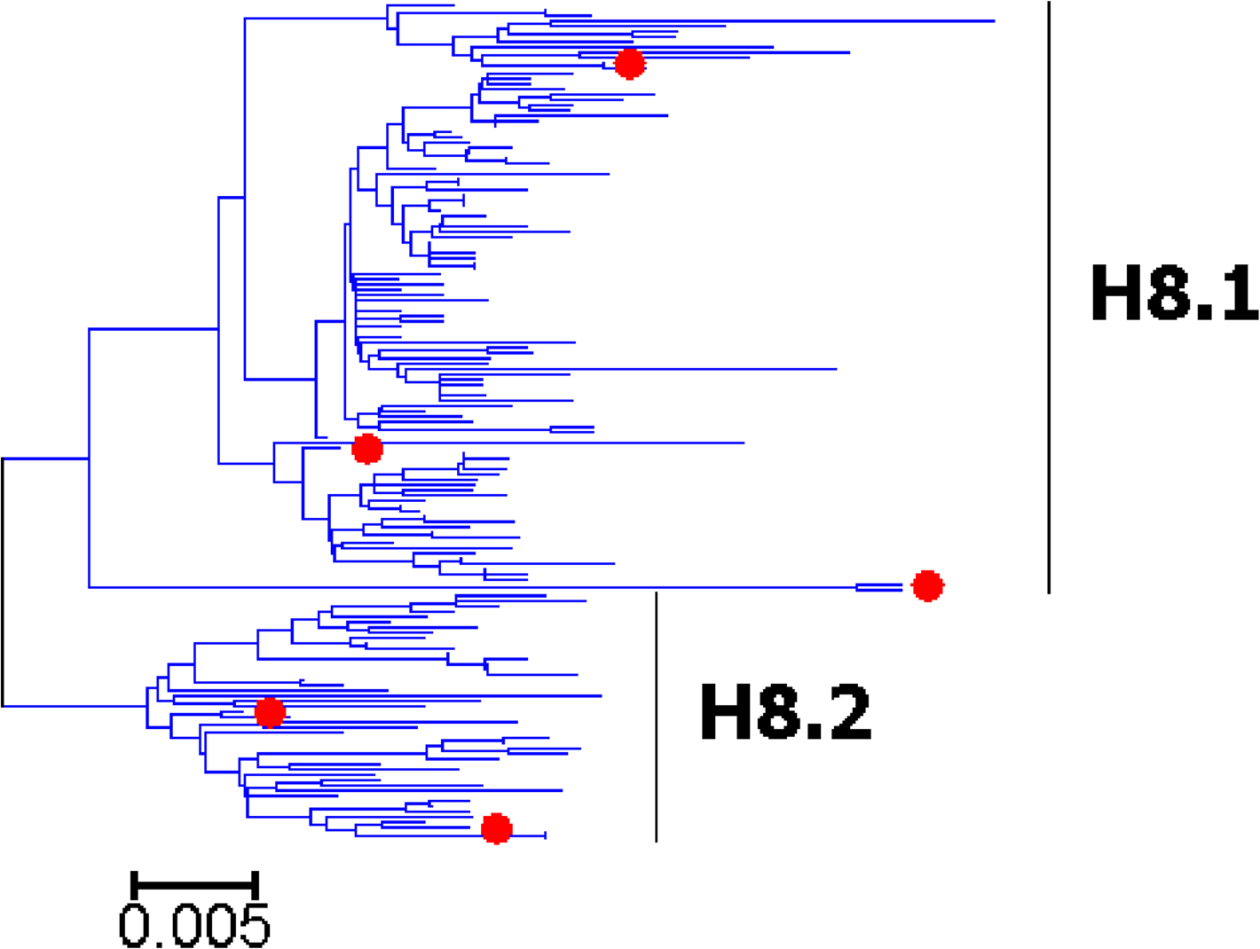
Phylogenetic diversity and distribution of H8 subtypes of IAVs based on HA sequences. H8 subtype IAVs were classified into two primary lineages, which mainly corresponded to AIVs circulating in the Western and Eastern Hemispheres

**Fig. 10:**
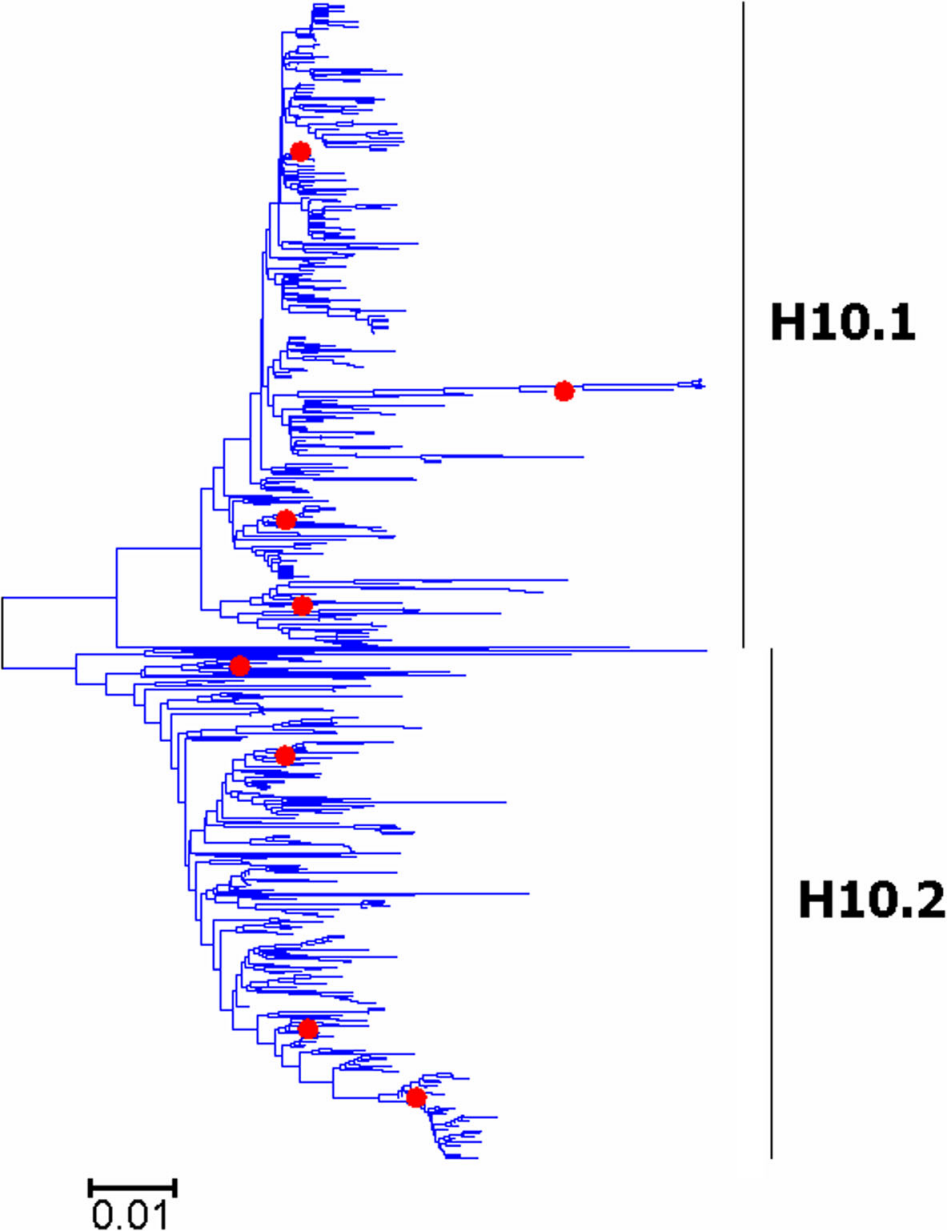
Phylogenetic diversity and distribution of H10 subtypes of IAVs based on HA sequences. H10 subtype IAVs were classified into two primary lineages, which mainly corresponded to AIVs circulating in the Western and Eastern Hemispheres

**Fig. 11:**
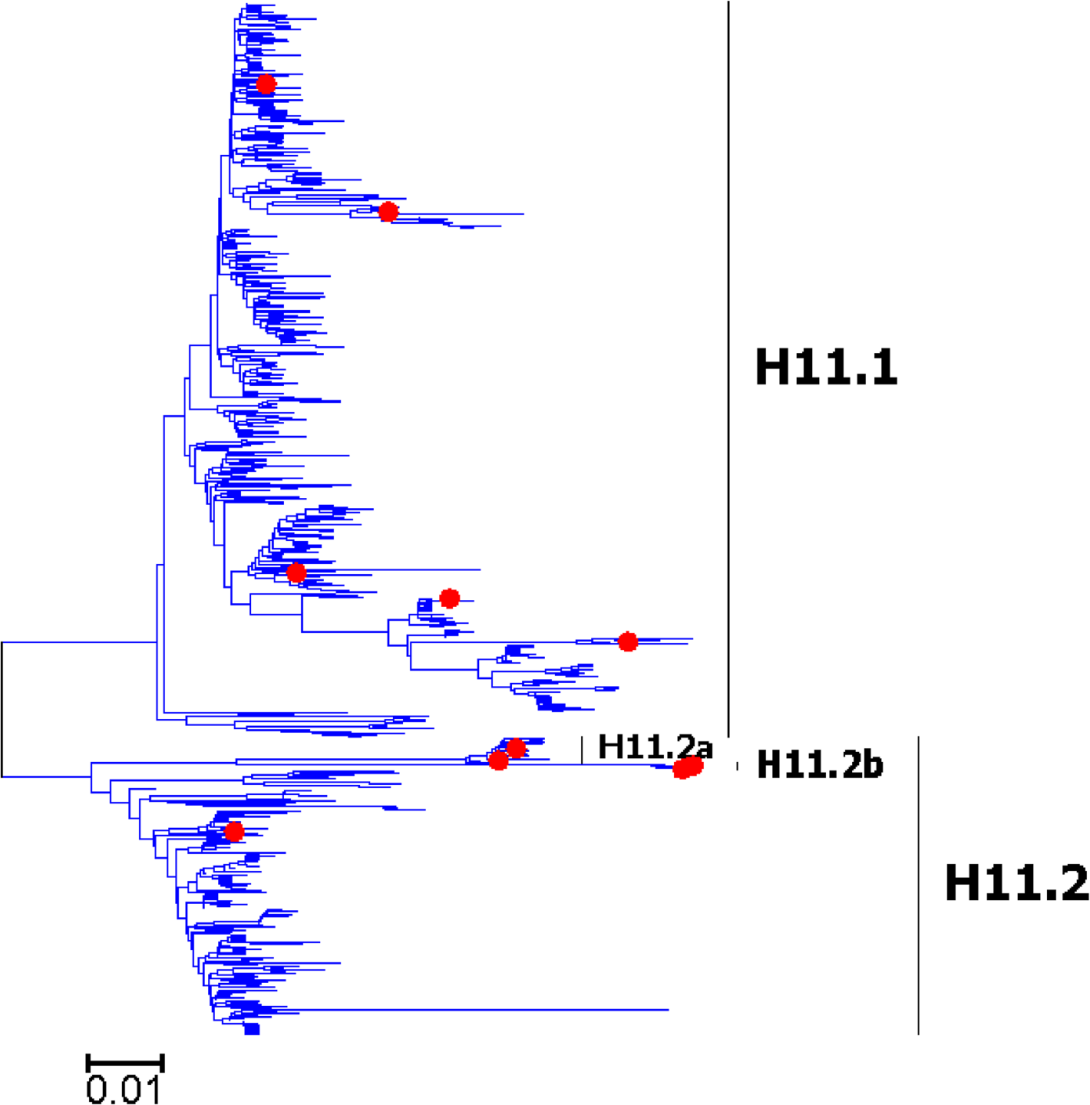
Phylogenetic diversity and distribution of H11 subtypes of IAVs based on HA sequences. H11 subtype IAVs were classified into two primary lineages, which mainly corresponded to AIVs circulating in the Western and Eastern Hemispheres

**Fig. 12:**
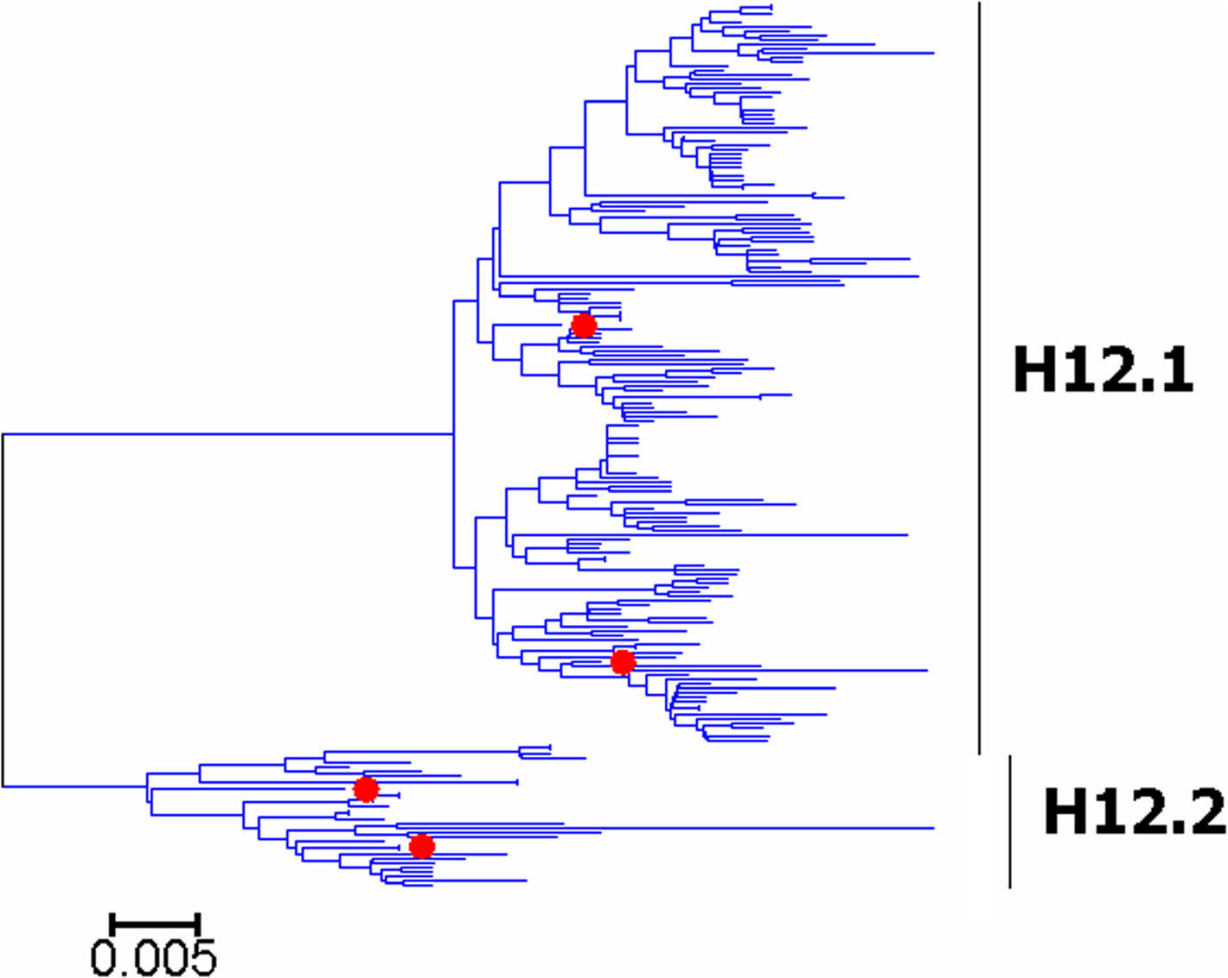
Phylogenetic diversity and distribution of H12 subtypes of IAVs based on HA sequences. H12 subtype IAVs were classified into two primary lineages, which mainly corresponded to AIVs circulating in the Western and Eastern Hemispheres

**Fig. 13:**
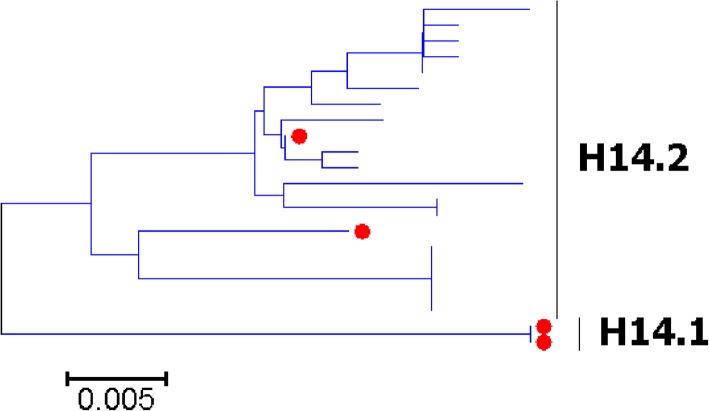
Phylogenetic diversity and distribution of H14 subtypes of IAVs based on HA sequences. H14 subtype IAVs were classified into two primary lineages, which mainly corresponded to AIVs circulating in the Western and Eastern Hemispheres

**Fig. 14:**
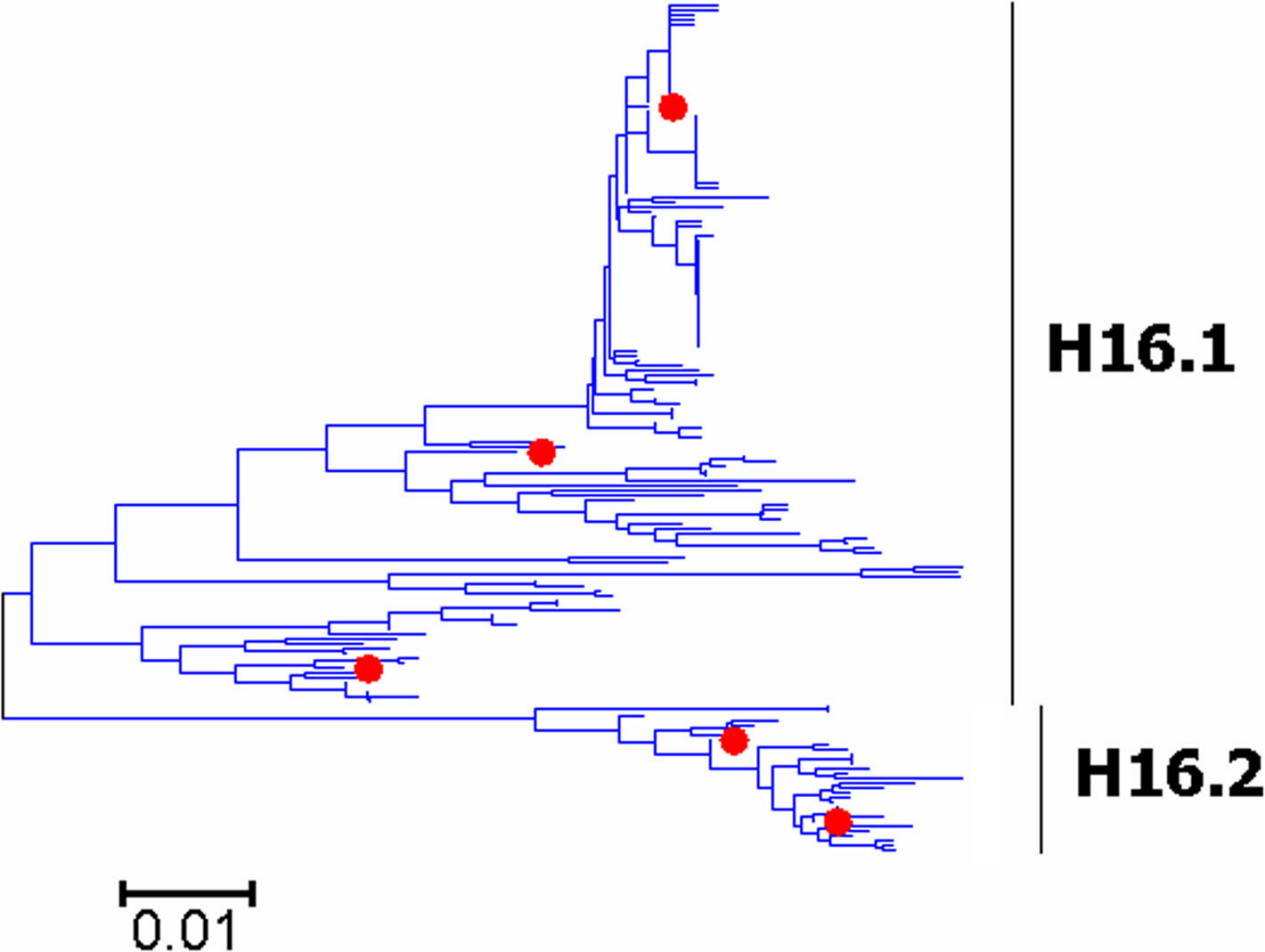
Phylogenetic diversity and distribution of H16 subtypes of IAVs based on HA sequences. H6 subtype IAVs were classified into two primary lineages, which mainly corresponded to AIVs circulating in the Western and Eastern Hemispheres

**Fig. 15:**
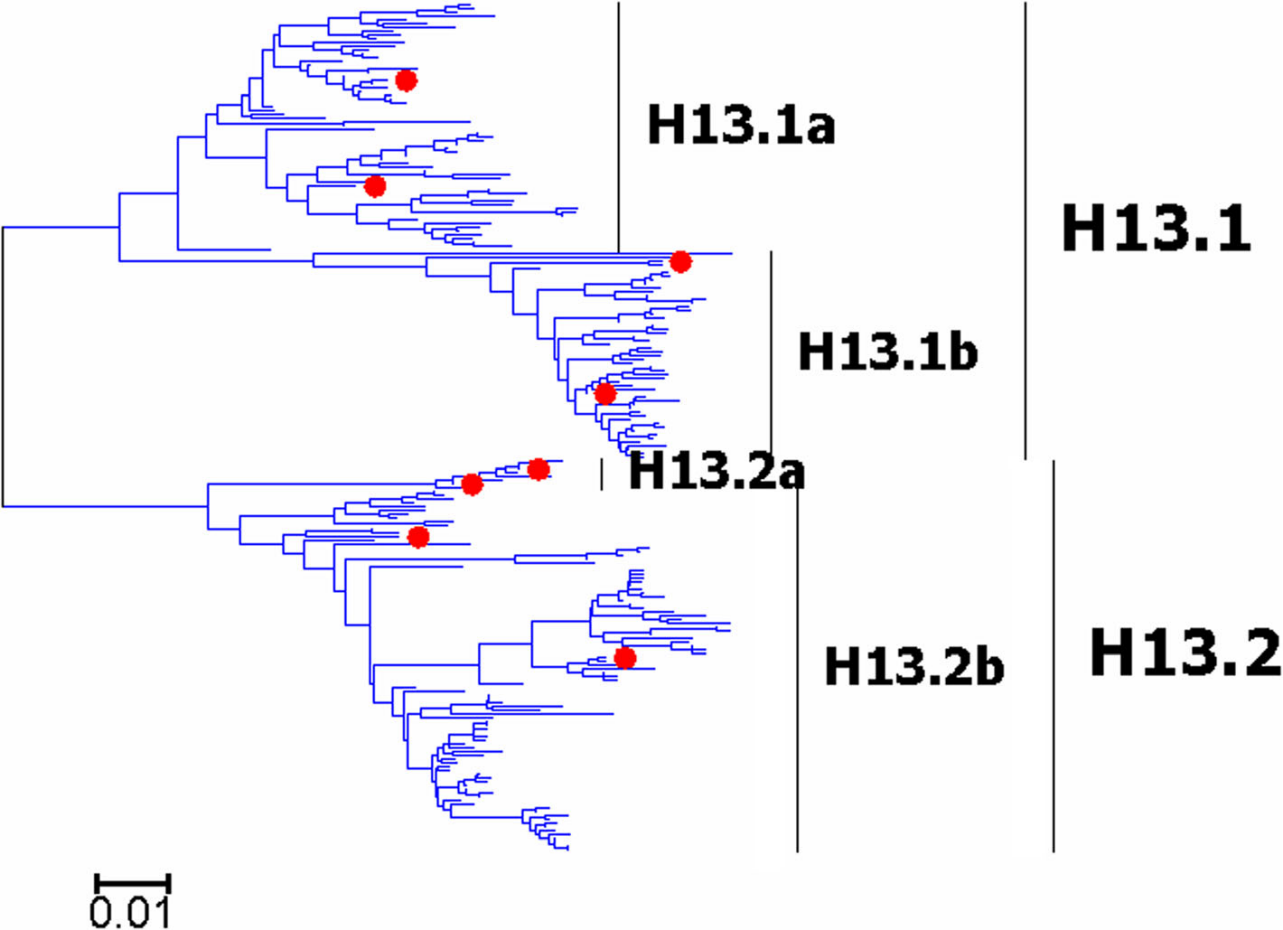
Phylogenetic diversity and distribution of H13 subtypes of IAVs based on HA sequences. H13 subtype IAVs were classified into two global primary lineages

**Fig. 16:**
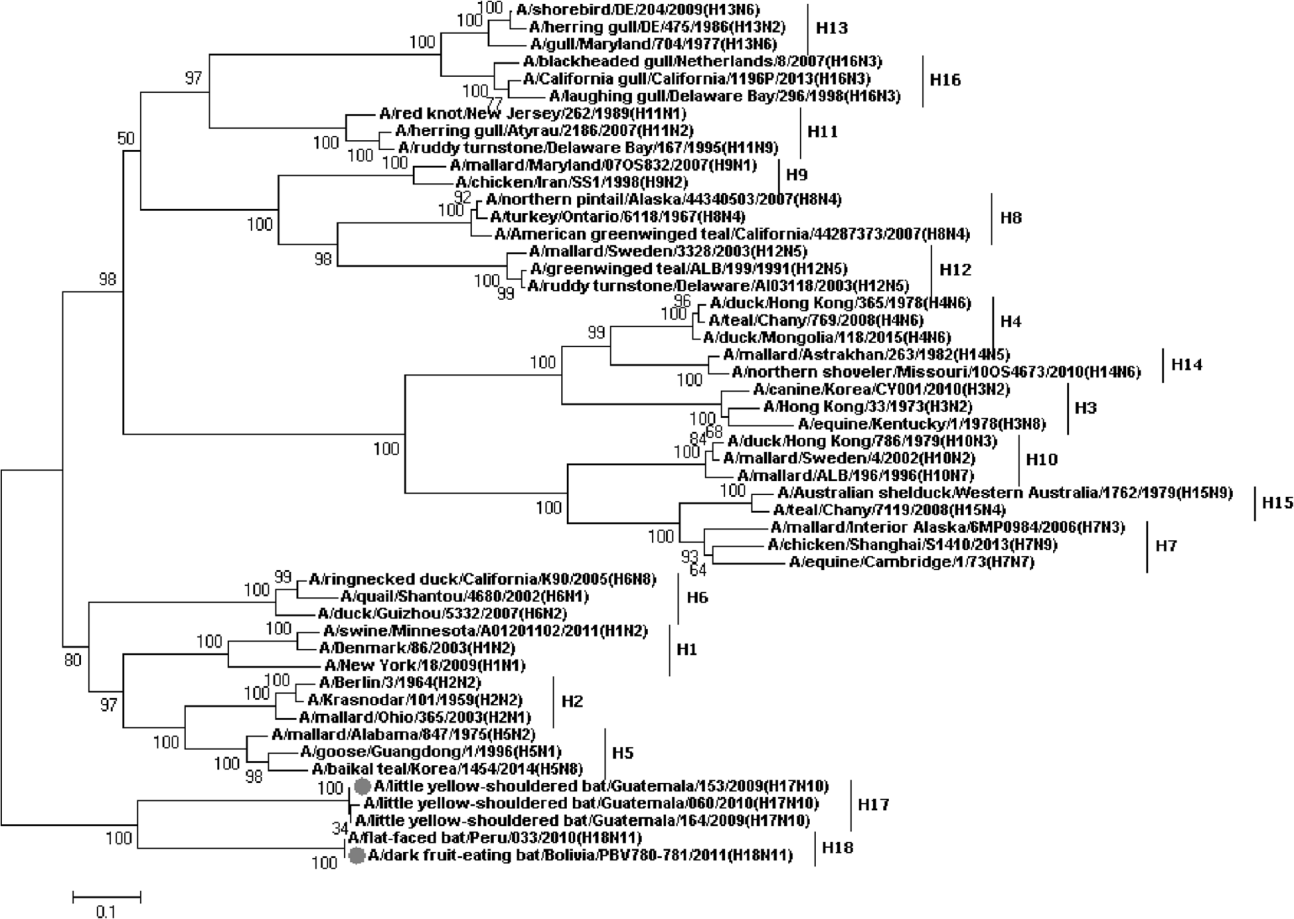
Phylogenetic diversity and distribution of H17 and H18 subtypes of IAVs based on HA sequences. The phylogenetic tree covered some sequences of H1-H16 subtypes as references

**Fig. 17:**
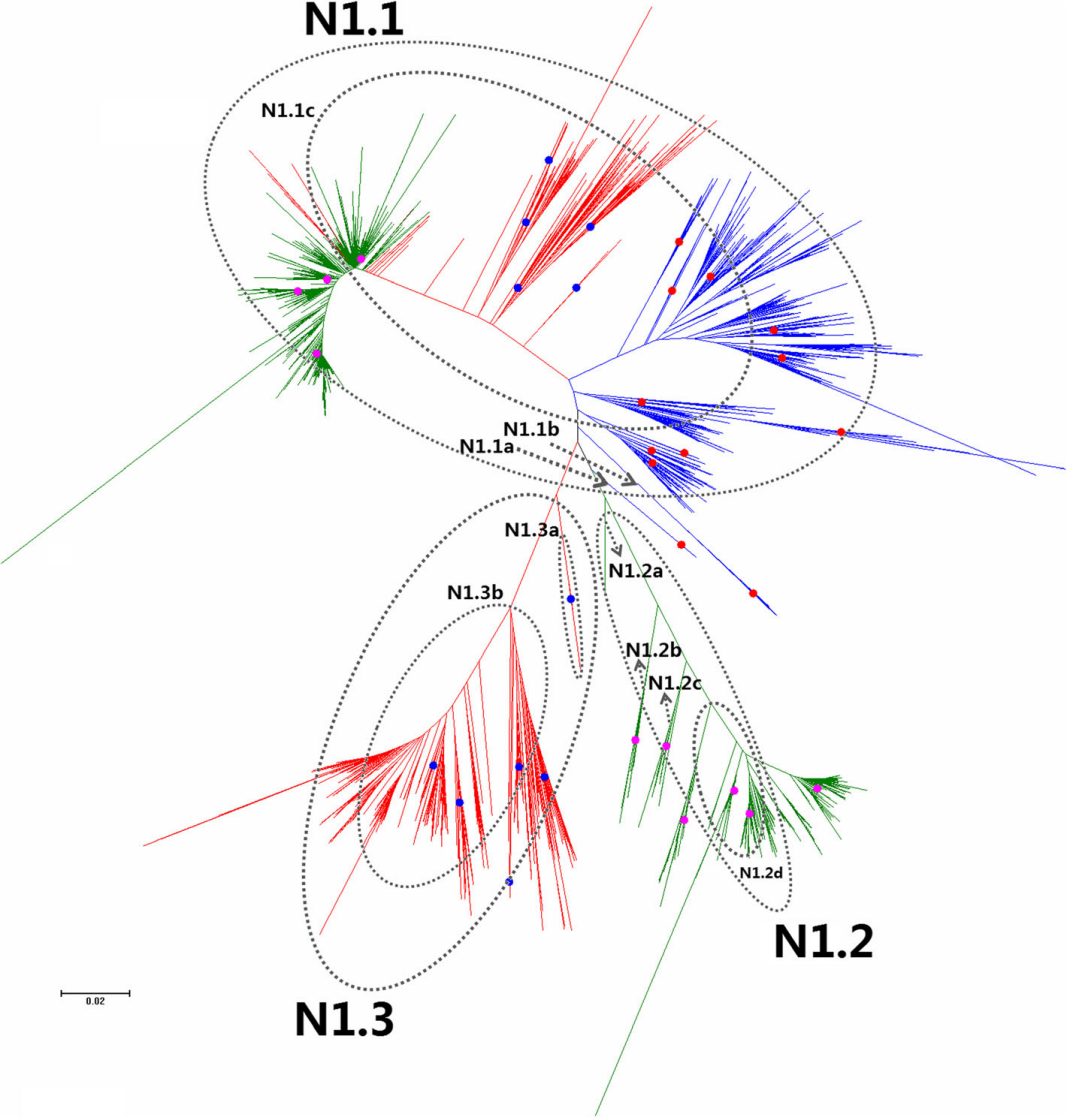
Phylogenetic diversity and distribution of N1 subtype IAVs based on NA sequences. N1 subtype IAVs were classified into three lineages, N1.1, N1.2 and N1.3, largely corresponding to avian, human, and classical swine N1 subtype IAVs

**Fig. 18:**
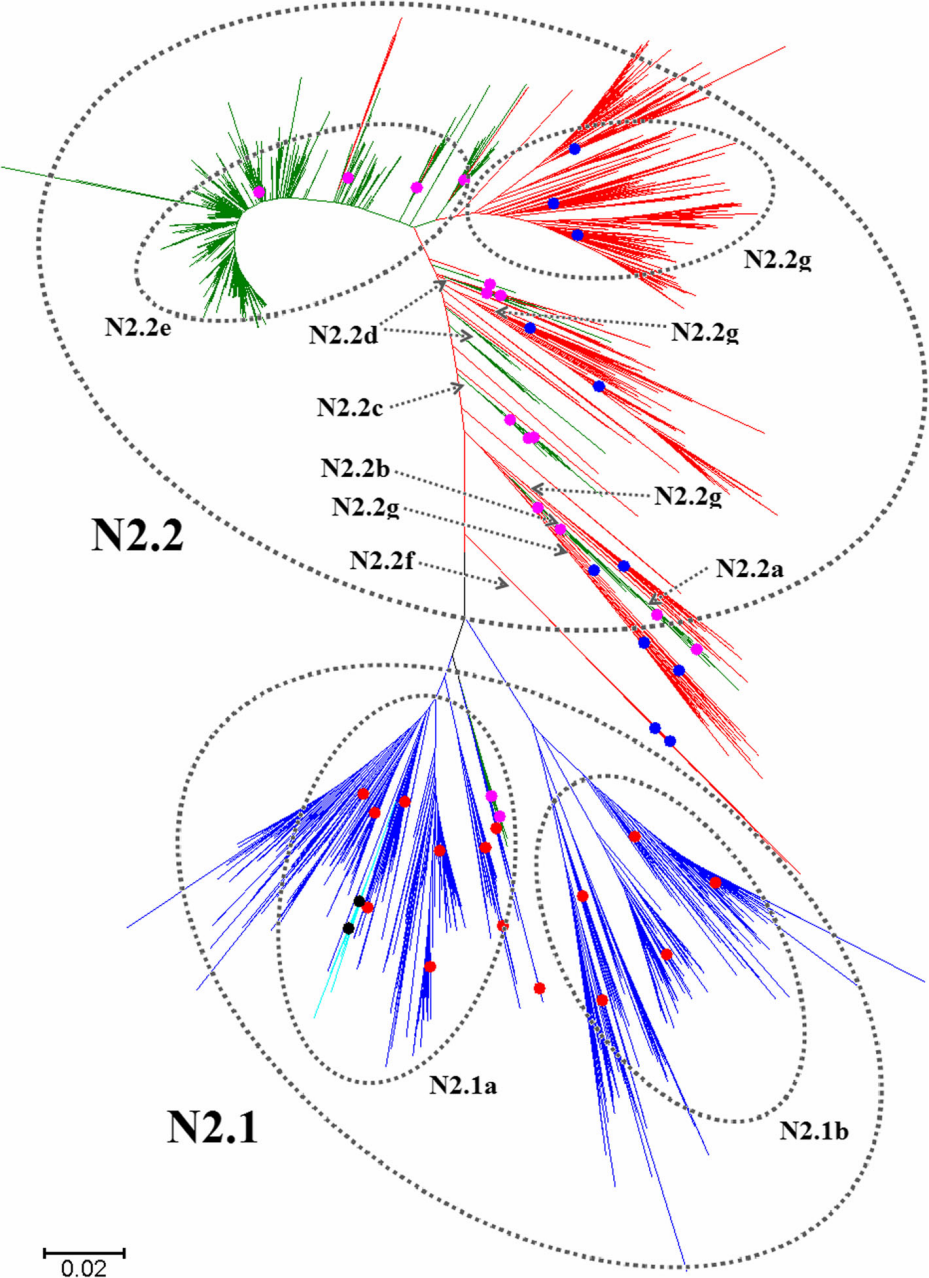
Phylogenetic diversity and distribution of N2 subtype IAVs based on NA sequences. N2 subtype IAVs were classified into two primary lineages, N2.1 and N2.2, largely corresponding to avian and human/swine IAVs

**Fig. 19:**
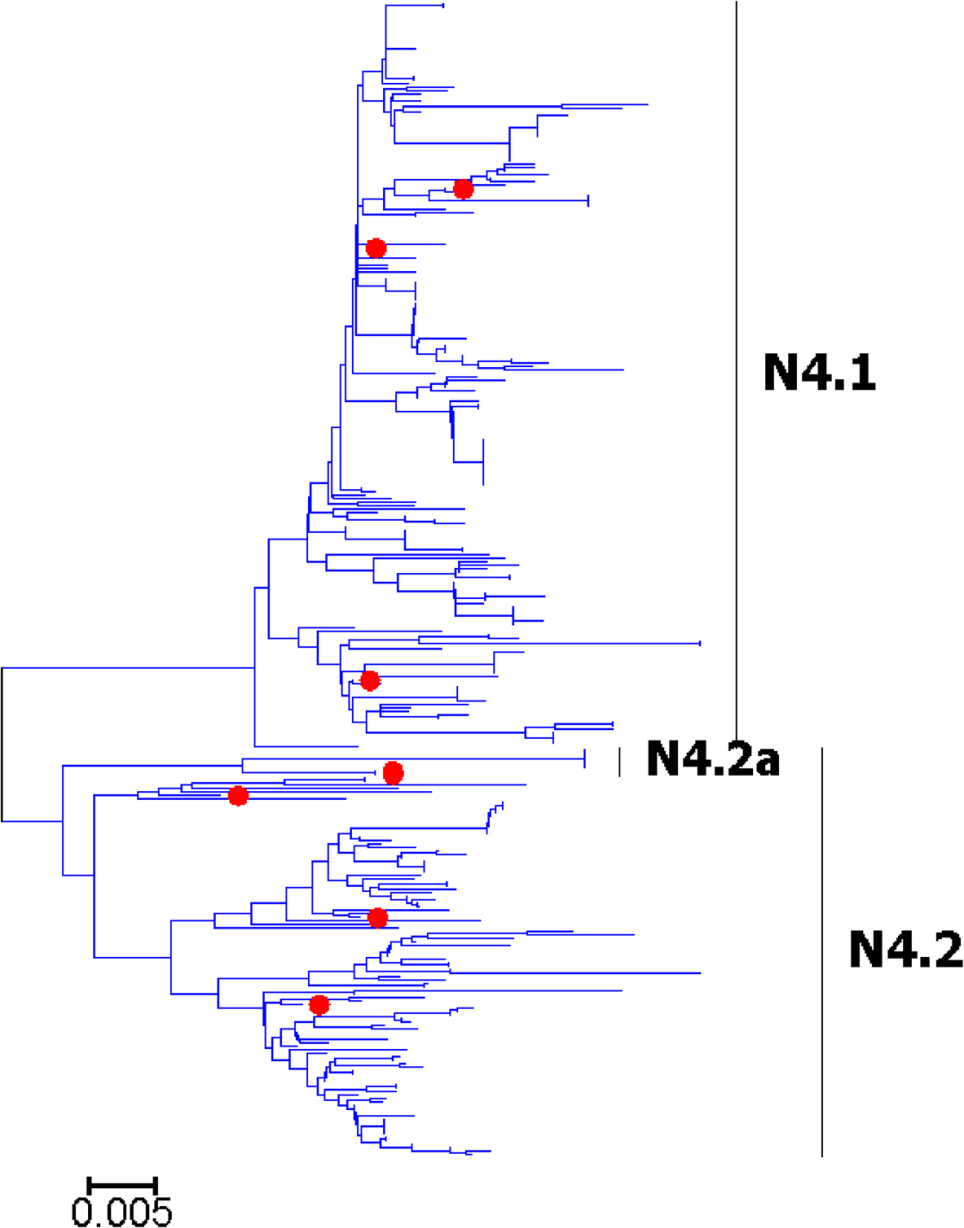
Phylogenetic diversity and distribution of N4 subtype IAVs based on NA sequences. N4 subtype IAVs were classified into two primary lineages, which mainly corresponded to the AIVs circulating in the Western and Eastern Hemispheres

**Fig. 20:**
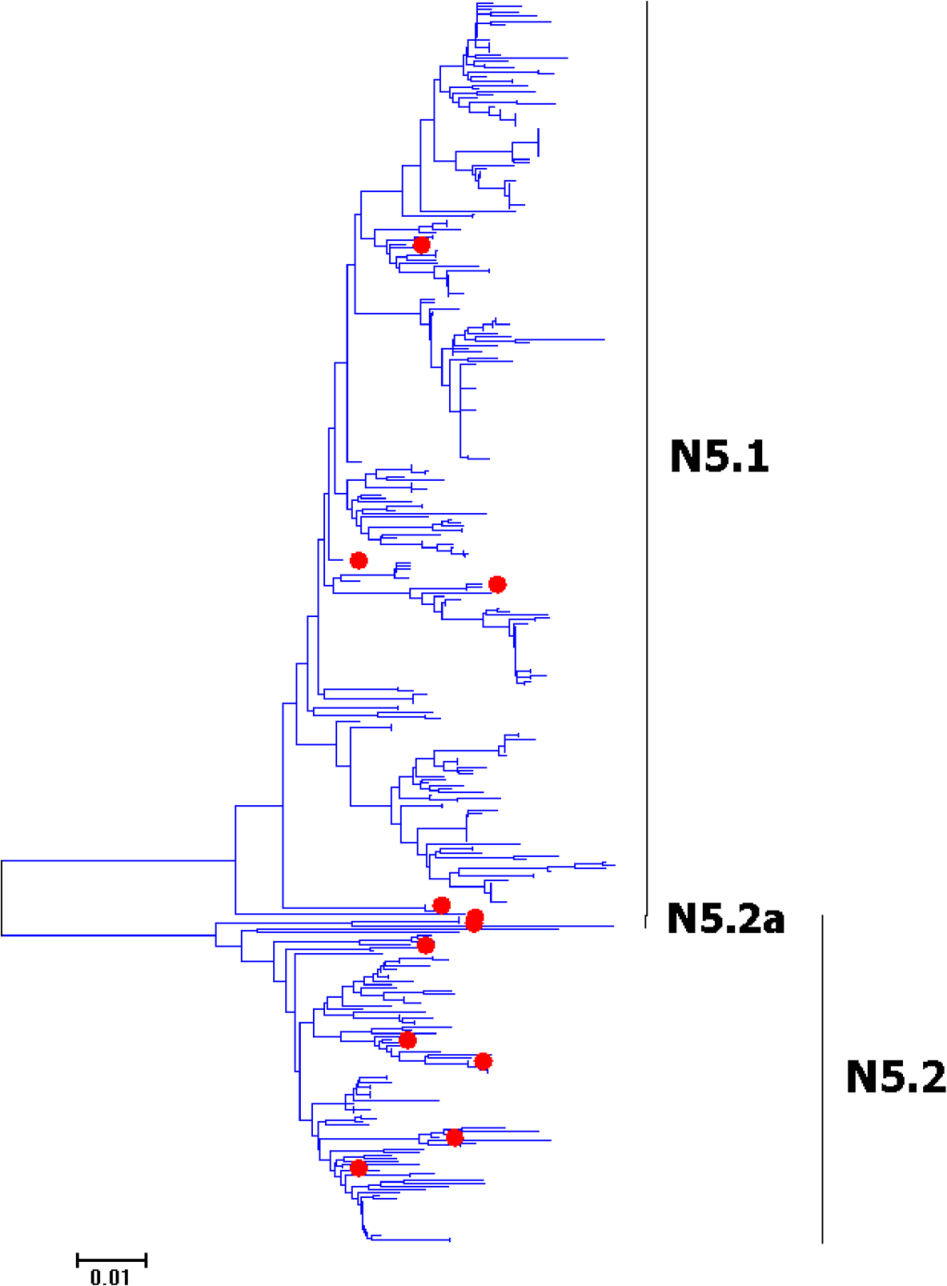
Phylogenetic diversity and distribution of N5 subtype IAVs based on NA sequences. N5 subtype IAVs were classified into two primary lineages, which mainly corresponded to the AIVs circulating in the Western and Eastern Hemispheres

**Fig. 21:**
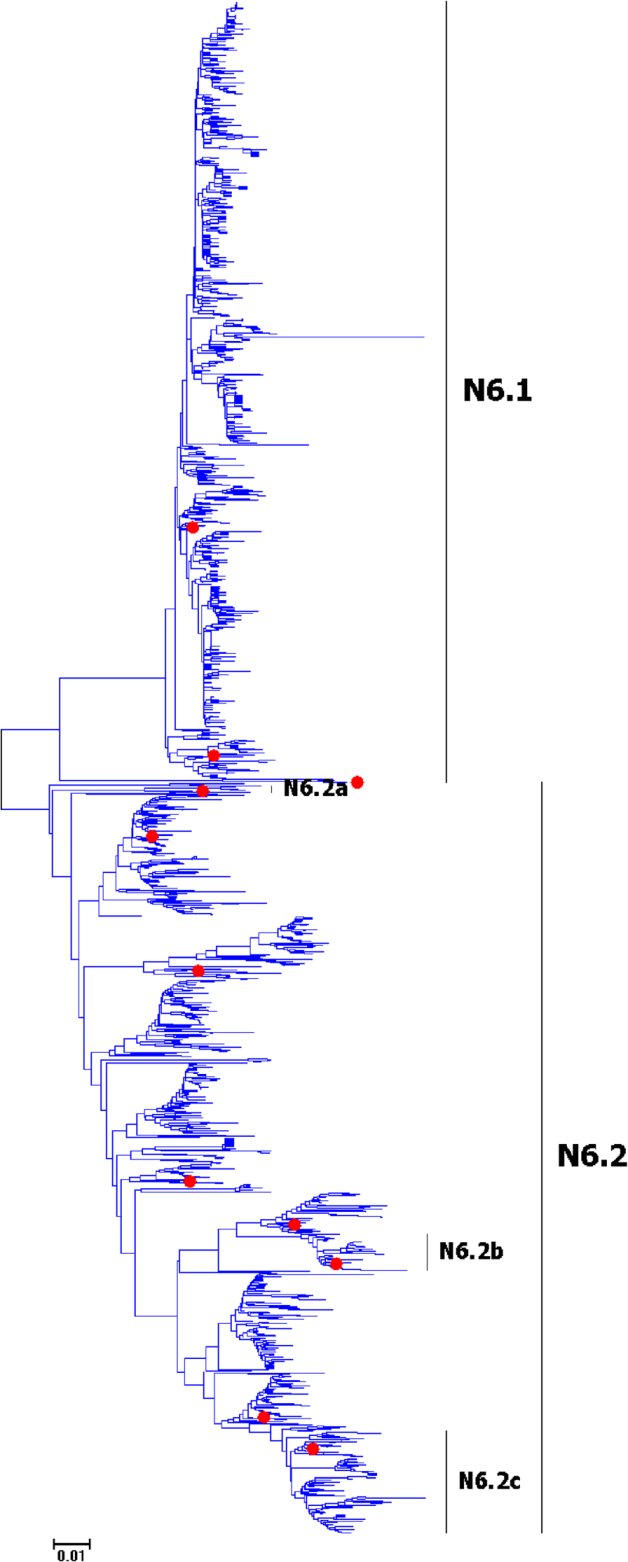
Phylogenetic diversity and distribution of N6 subtype IAVs based on NA sequences. N6 subtype IAVs were classified into two primary lineages, which mainly corresponded to the AIVs circulating in the Western and Eastern Hemispheres

**Fig. 22:**
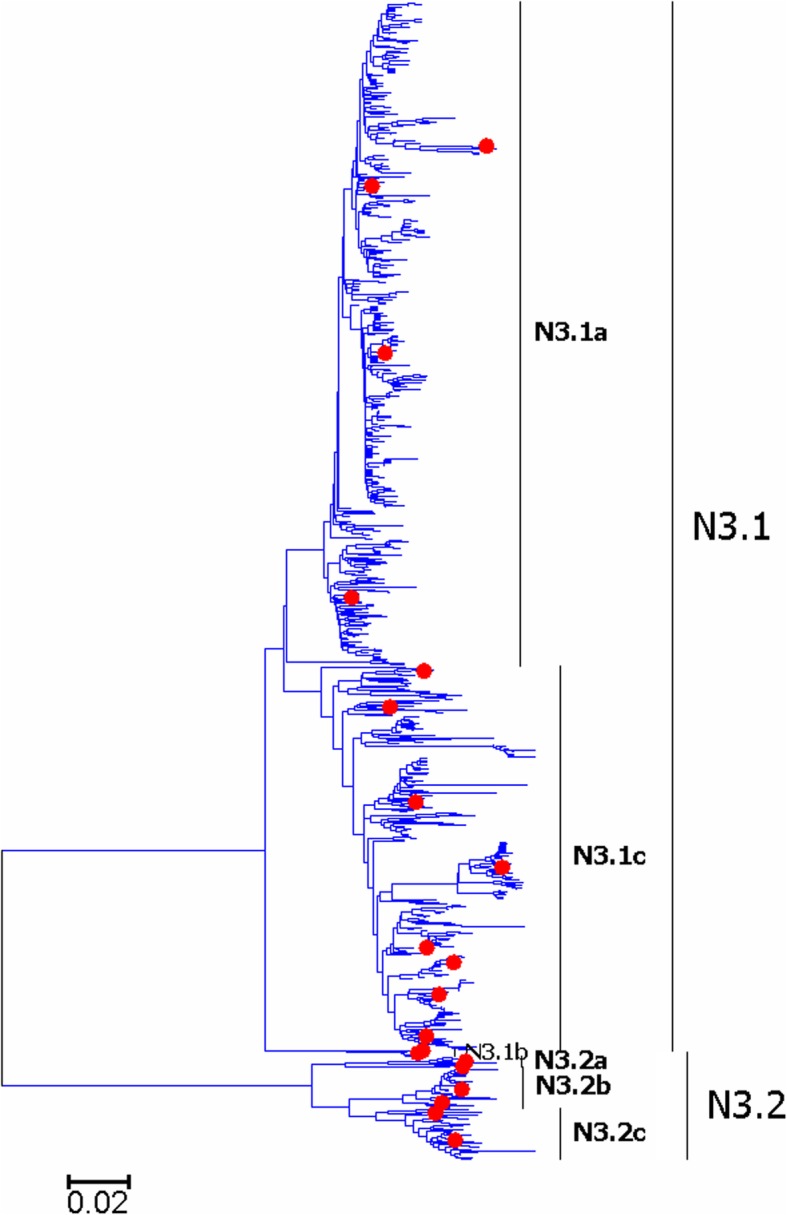
Phylogenetic diversity and distribution of N3 subtype IAVs based on NA sequences. N3 subtype IAVs were classified into two primary lineages

**Fig. 23:**
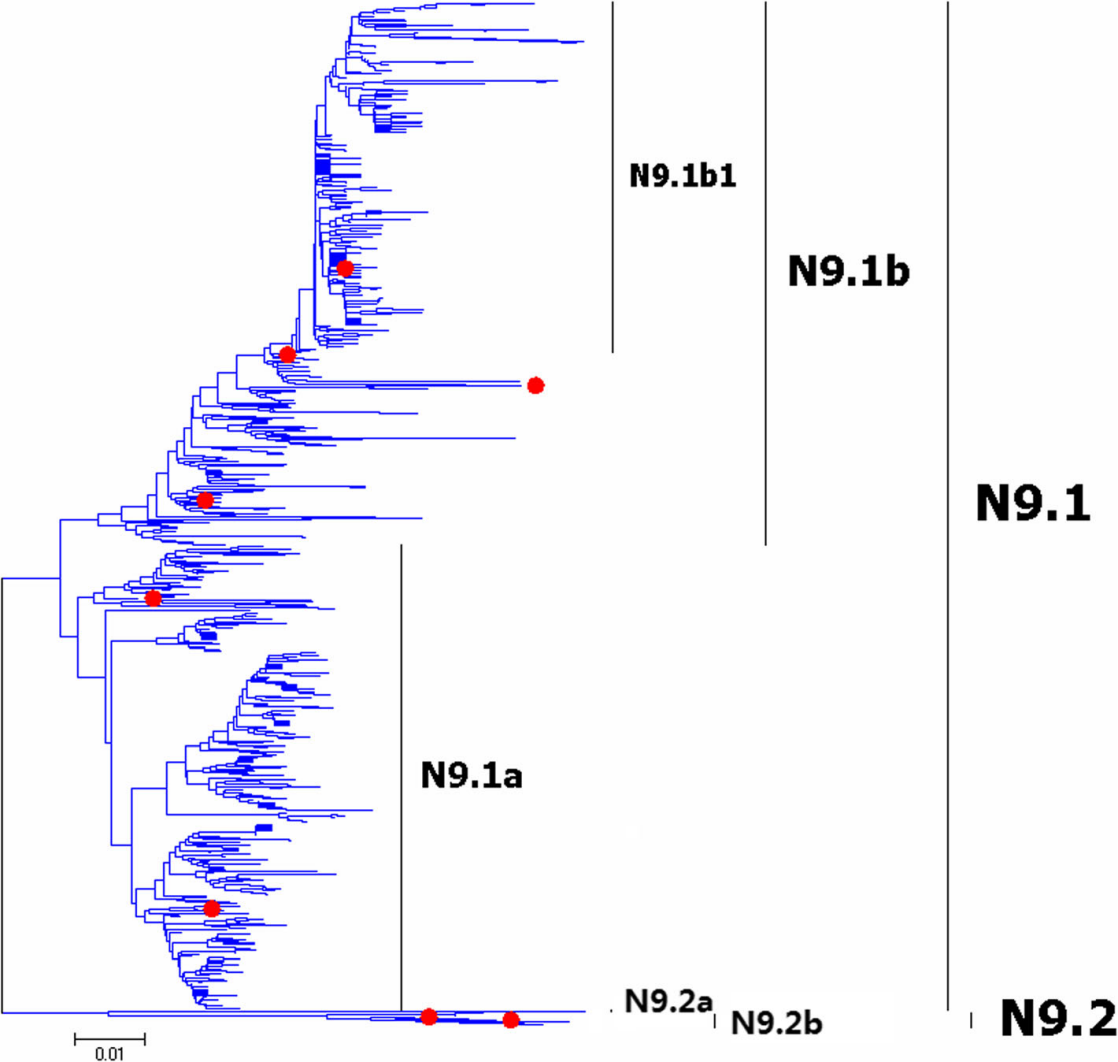
Phylogenetic diversity and distribution of N9 subtype IAVs based on NA sequences. N9 subtype IAVs were classified into two primary lineages

**Fig. 24:**
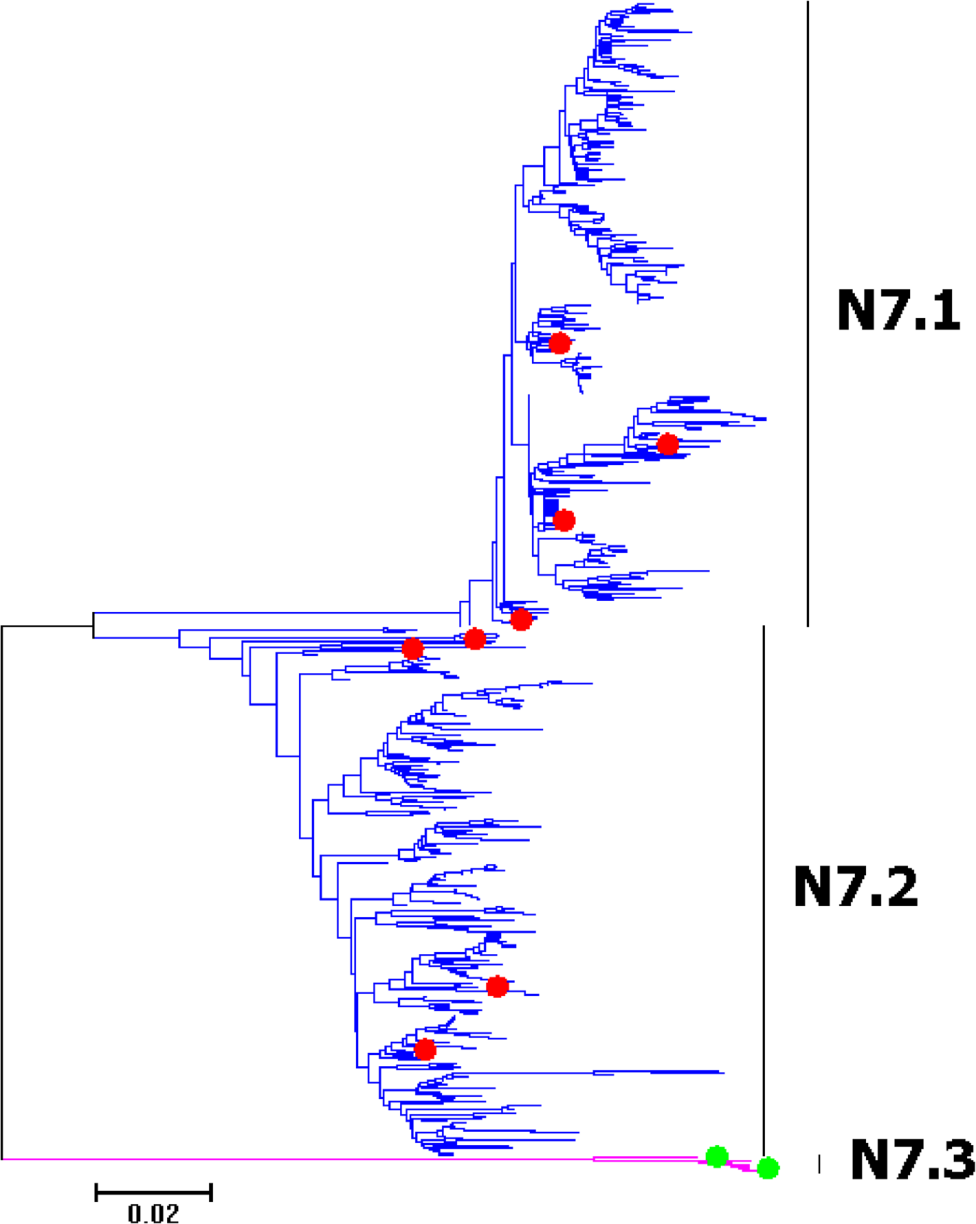
Phylogenetic diversity and distribution of N7 subtype IAVs based on NA sequences. N7 subtype IAVs were classified into three primary lineages. N7.1 corresponded to AIVs circulating in the Western Hemisphere; N7.2 corresponded to AIVs circulating in the Eastern Hemisphere; N7.3corresponded to EIVs

**Fig. 25:**
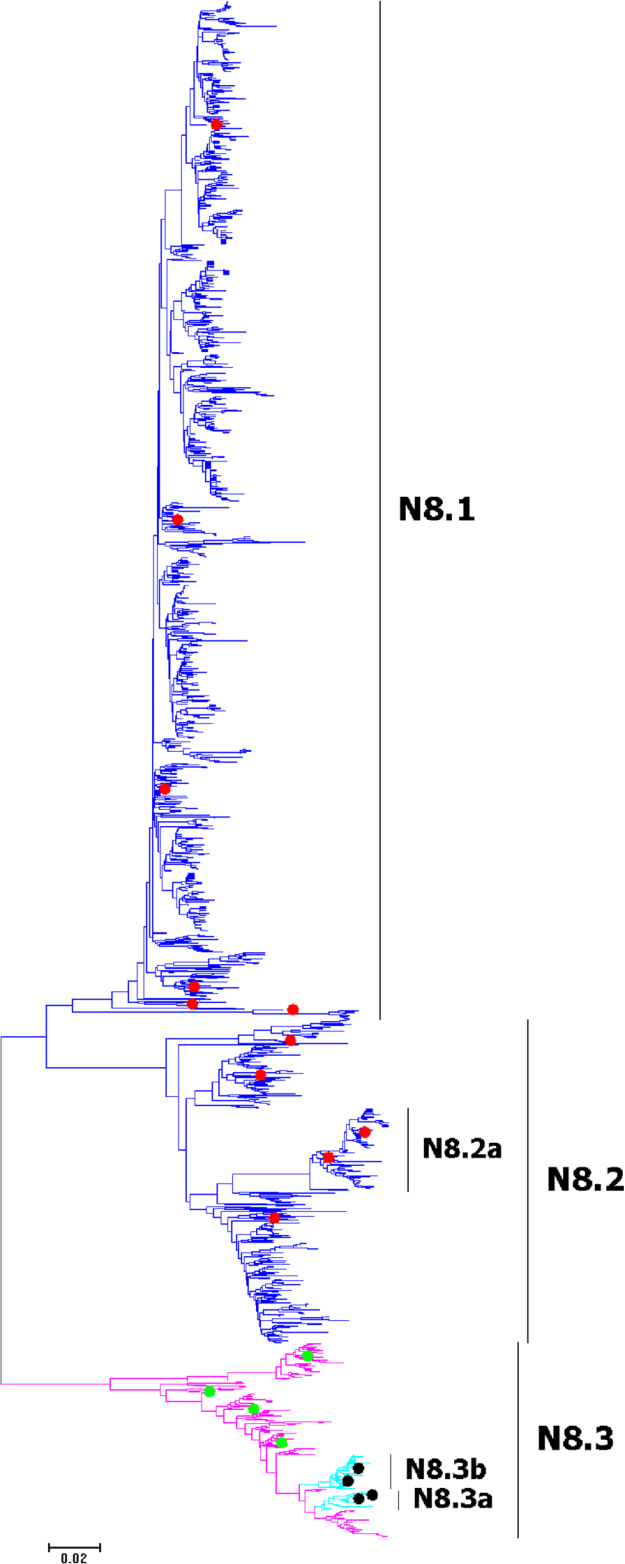
Phylogenetic diversity and distribution of N8 subtype IAVs based on NA sequences. N8 subtype IAVs were classified into three primary lineages. N8.1 corresponded to AIVs circulating in the Western Hemisphere; N8.2 corresponded to AIVs circulating in the Eastern Hemisphere; N8.3 corresponded to EIVs

**Fig. 26:**
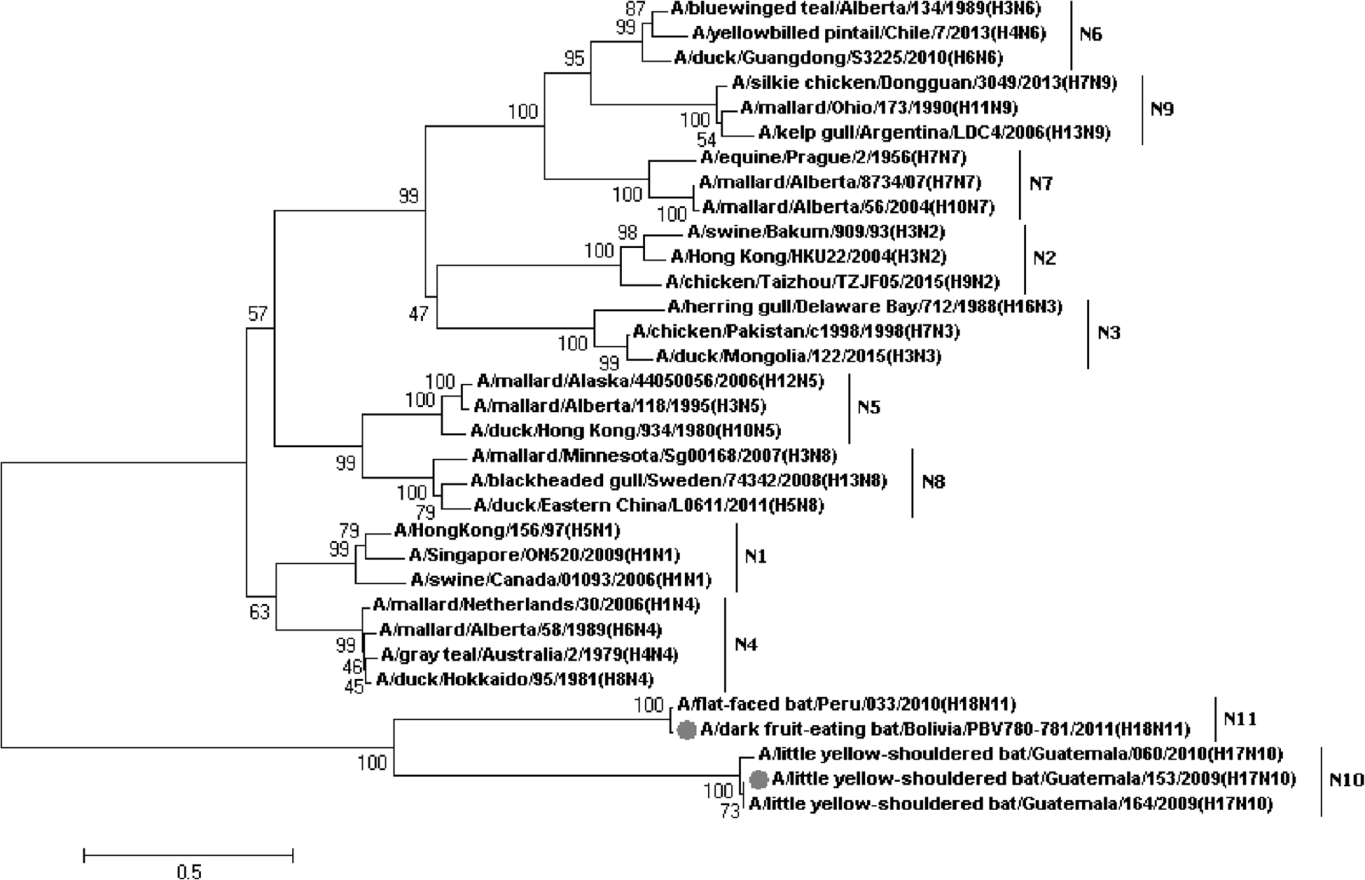
Phylogenetic diversity and distribution of N10 and N11 subtype IAVs based on NA sequences. The phylogenetic tree covered some sequences of N1-N9 subtypes as references

**Fig. 27:**
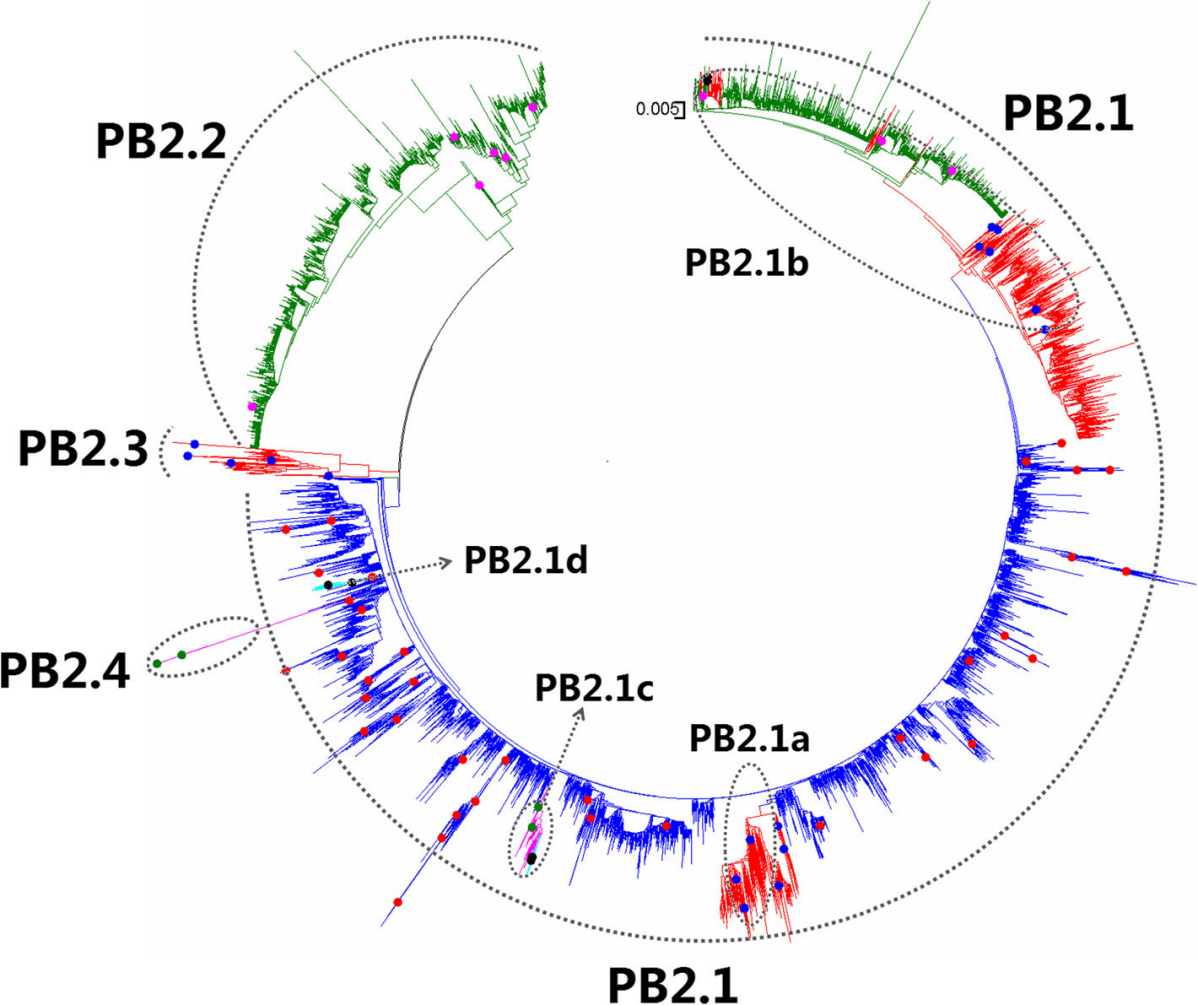
Phylogenetic diversity and distribution of IAVs based on PB2 gene sequences. Based on the viral PB2 gene sequences, IAVs were classified into four primary lineages, PB2.1, PB2.2, PB2.3, and PB2.4 which mainly corresponded to AIVs, HuIVs, SIVs, and EIVs

**Fig. 28:**
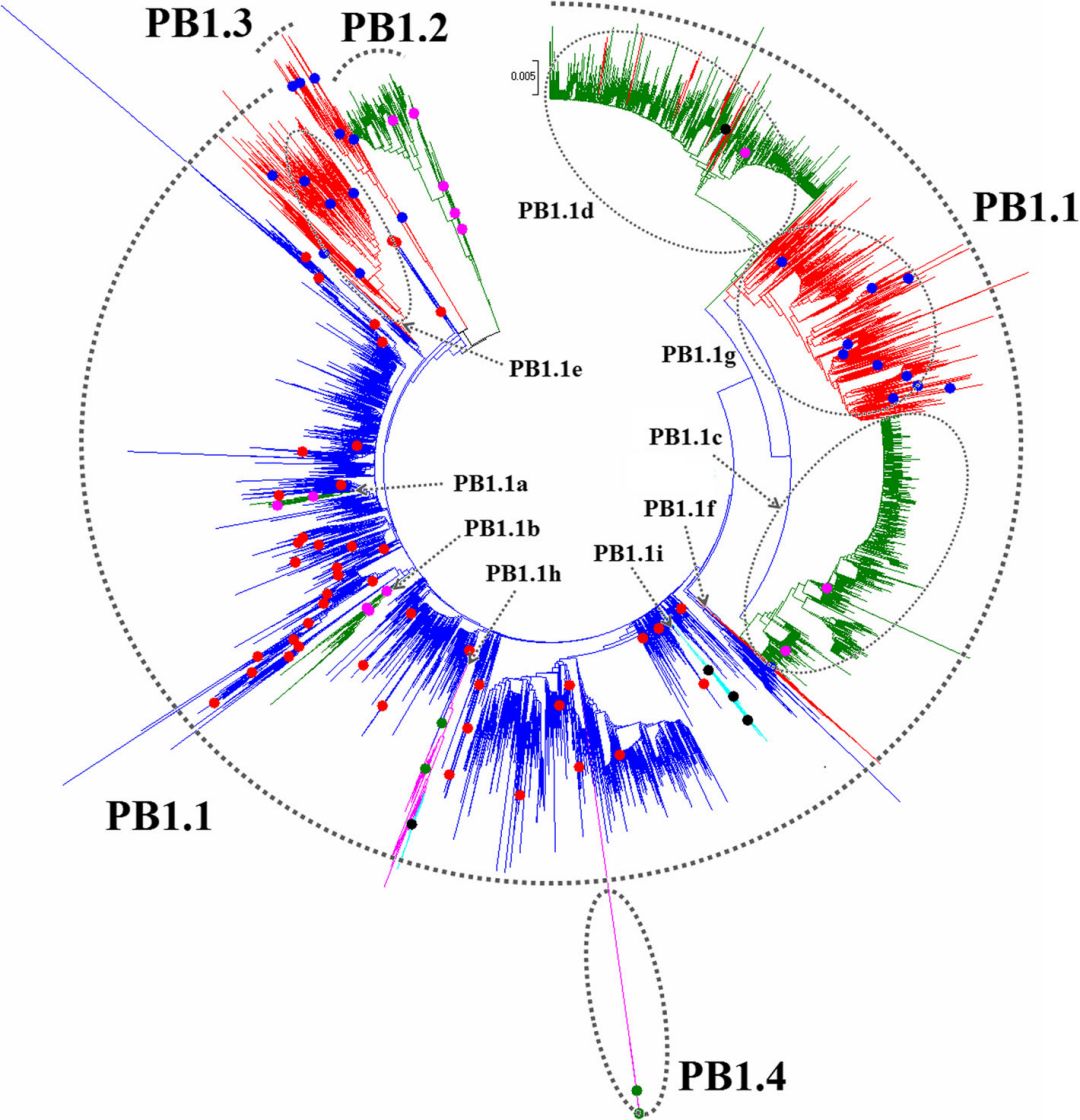
Phylogenetic diversity and distribution of IAVs based on PB1 gene sequences. Based on the viral PB1 gene sequences, IAVs were classified into four primary lineages, PB1.1, PB1.2, PB1.3, and PB1.4 which mainly corresponded to AIVs, HuIVs, SIVs, and EIVs

**Fig. 29:**
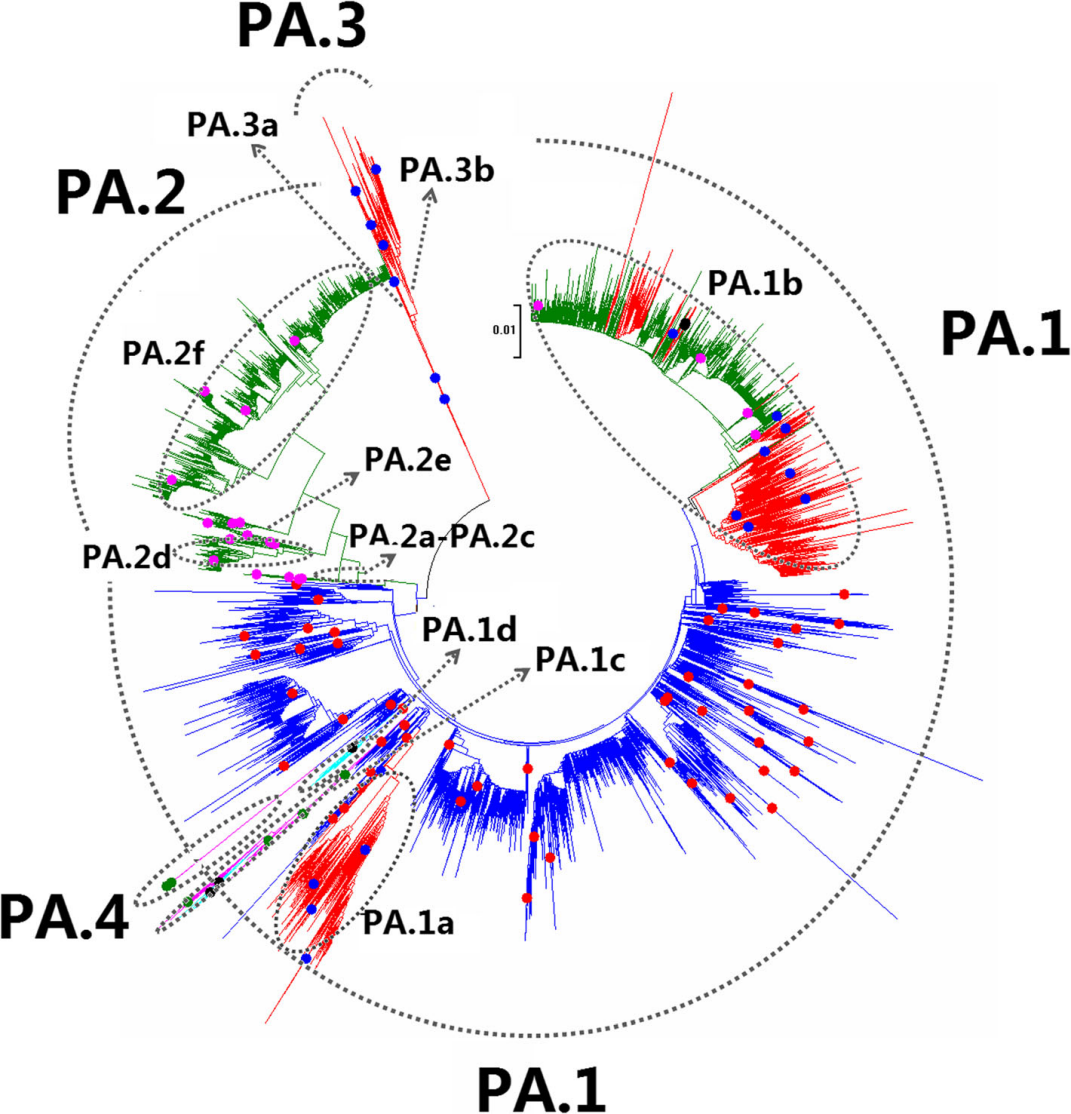
Phylogenetic diversity and distribution of IAVs based on PA gene sequences. Based on the viral PA gene sequences, IAVs were classified into four primary lineages, PA.1, PA.2, PA.3, and PA.4 which mainly corresponded to AIVs, HuIVs, SIVs, and EIVs

**Fig. 30:**
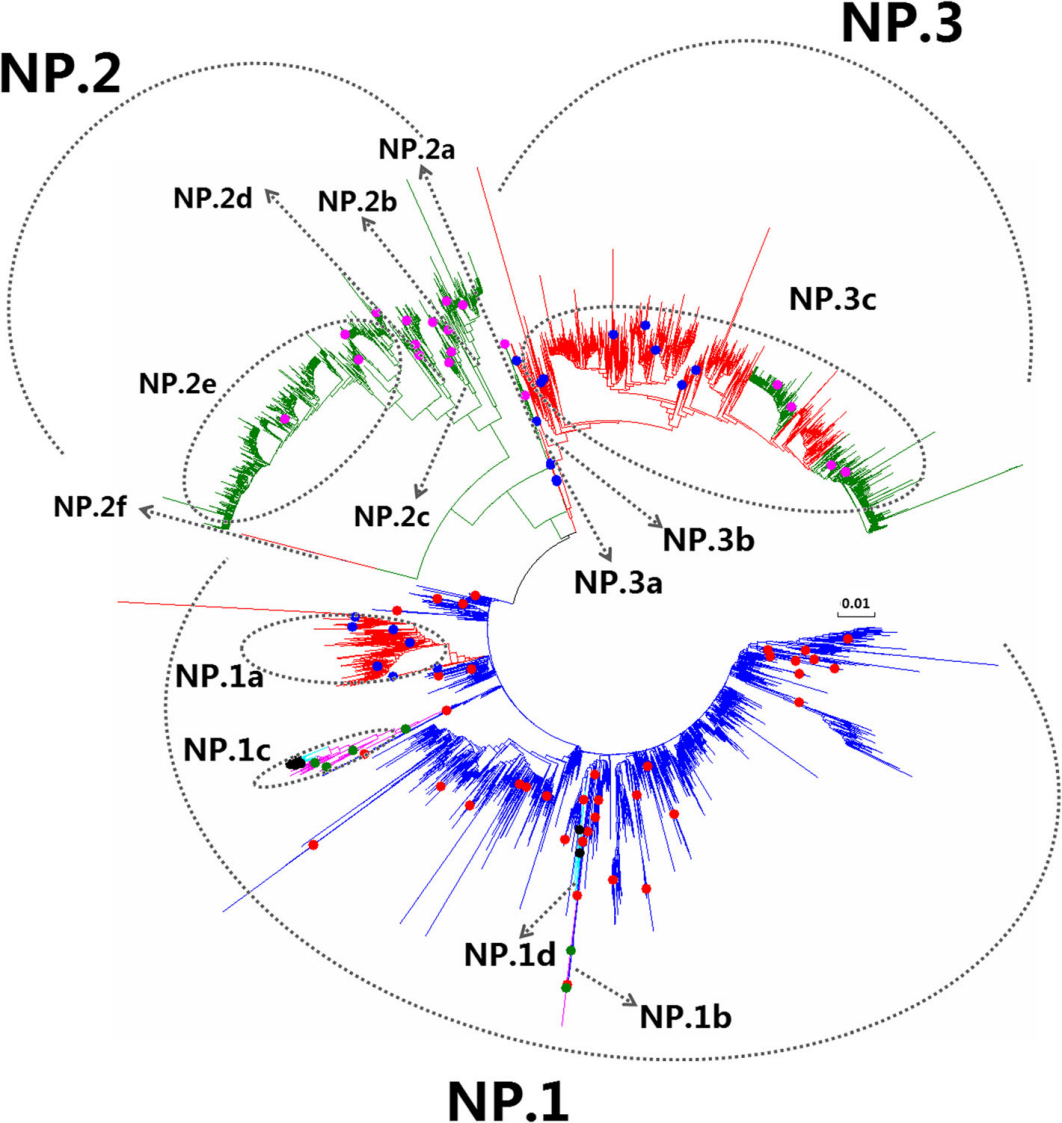
Phylogenetic diversity and distribution of IAVs based on NP gene sequences. Based on the viral NP gene sequences, IAVs were classified into four primary lineages, NP.1, NP.2 and NP.3 which mainly corresponded to AIVs, HuIVs and SIVs

**Fig. 31:**
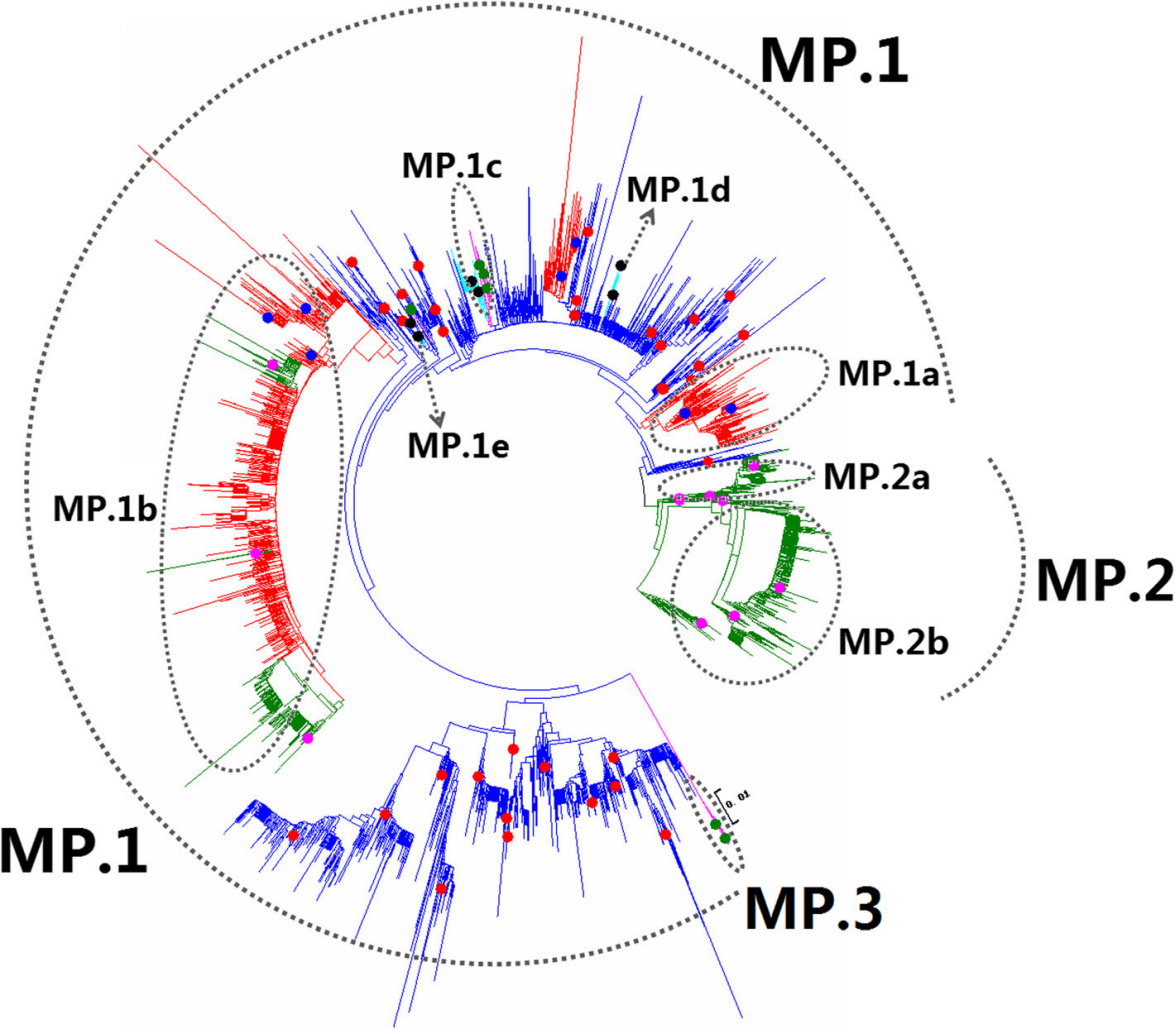
Phylogenetic diversity and distribution of IAVs based on MP gene sequences. Based on the viral MP gene sequences, IAVs were classified into three primary lineages, MP.1, MP.2 and MP.3 which mainly corresponded to AIVs, HuIVs and EIVs

**Fig. 32:**
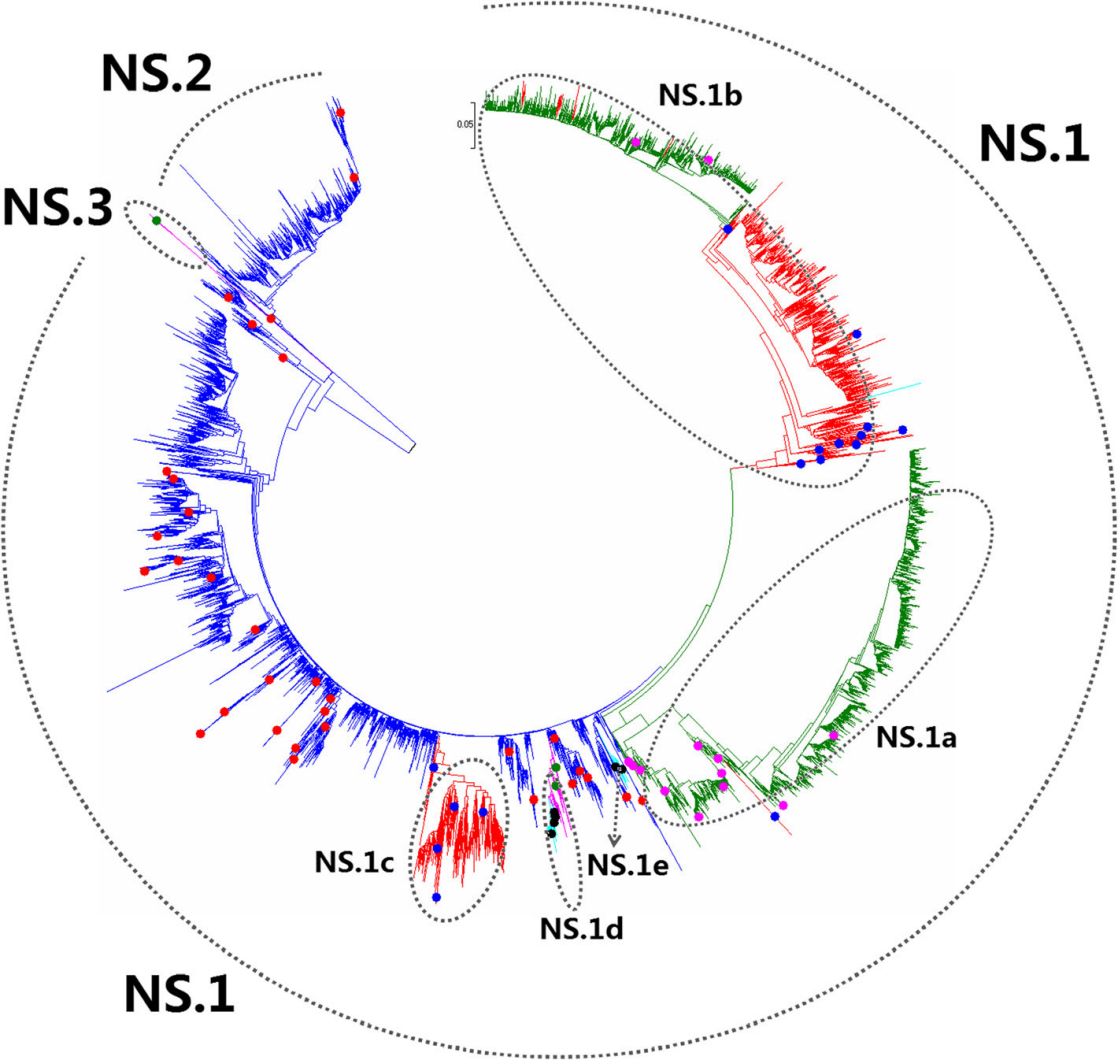
Phylogenetic diversity and distribution of IAVs based on NS gene sequences. Based on the viral NS gene sequences, IAVs were classified into three primary lineages, NS.1, NS.2 and NS.3 which mainly corresponded to AIVs, AIVs and EIVs

### Phylogenetic diversity and distribution of H1 subtype IAVs based on HA sequences

Figure 1 (the thumbnail of the phylogenetic tree) and Additional file 1: Figure SI_1 (the original phylogenetic tree) both suggested that, from a panorama view, H1 subtype IAVs could be classified into two primary lineages, H1.1 and H1.2, based on their HA sequences.

H1.1 mainly corresponded to H1 subtype AIVs and some SIVs which originated from the AIVs. Four secondary lineages within H1.1, H1.1a-H1.1d, were designated. H1.1a mainly corresponded to AIVs from North American with some exceptions, e.g. one clade within H1.1a represented by A/duck/Bavaria/1/1977(H1N1) were AIVs from the Eastern Hemisphere, and one clade within H1.1a represented by A/northern shoveler/California/K168/2005(H1N9) containing some AIVs from the Eastern Hemisphere and a few isolates from pigs. H1.1b corresponded to some AIVs from South America. H1.1c mainly corresponded to AIVs from the Eastern Hemisphere except the strain A/swine/Eire/89/1996(H1N1). H1.1d mainly corresponded to the SIVs which were previously designated as H1 subtype Eurasian avian-like SIVs. SIVs within H1.1d exclusively circulated in the Eastern Hemisphere except the strain of A/swine/Mexico/AVX47/2013(H1N1). One avian strain (A/turkey/Netherlands/543301/1999), two human strains and one strain from wild boars were also located within H1.1d.

Lineage H1.2 corresponded to classical H1 subtype HuIVs and SIVs. Four secondary lineages (H1.2a-H1.2d) were further designated within H1.2. H1.2a corresponded to one HuIV in 1918. H1.2b corresponded to some SIVs mainly circulating worldwide during the period 1930–1945, except three SIVs isolated in 1990, 2008 and 2009, respectively. H1.2c corresponded to the classical H1 subtype SIVs which have been circulating worldwide for decades. One strain within H1.2c became the pandemic H1N1 subtype HuIVs in 2009 which was designated as A(H1N1)pdm09 below. Nowadays, this group of viruses designated as H1.2c1 here has become the seasonal H1 subtype HuIVs.

H1.2d covered multiple groups of HuIVs which circulated in humans during the periods from the 1930s to the 1950s and from the 1970s to 2010. H1.2d also covered multiple groups of SIVs which were mainly isolated after 2010. Although some groups of HuIVs in H1.2d were inserted by swine groups in the phylogenetic tree (Additional file 1: Figure SI_1), this should be mainly due to that some SIVs were similar to some HuIVs in protein sequences by chance rather than through cross-species transmission. That is to say, cross-species transmission of the viruses in H1.2d did exist, but cross-species transmission has not caused frequent replacement of HuIV lineages by SIV lineages in H1.2d. This is because most HuIVs were in human-to-human transmission, and most SIVs were in swine-to-swine transmission, according to their epidemiology. On the other side, the average protein sequence identity of the HuIVs in H1.2d of the same decade after 1980 was around 96%, and its counterpart of the SIVs in H1.2d was around 93%, suggesting that SIVs in H1.2d were of more diversity than HuIVs in H1.2d (*P* < 0.01, by T test), and therefore it was possible that some SIVs were similar to some HuIVs by chance. With these considerations, we designated HuIV groups in H1.2d as H1.2d1, and SIV groups in H1.2d as H1.2d2, according to their hosts, although these two subordinate lineages inserted into each other in the phylogenetic tree. Nevertheless, the insertion suggested that H1.2d1 and H1.2d2 were of the same origin, and that the prevalent on-going circulation of the SIVs in H1.2d should pose a considerable threat to humans in the future even though H1.2d has disappeared in humans for years. Three quaternary subordinate lineages were designated within H1.2d2: H1.2d2a corresponded to SIVs in Europe, and H1.2d2b corresponded to SIVs in England, and H1.2d2c corresponded to SIVs in America.

In general, H1 subtype AIVs were exclusively located within H1.1a-H1.1c with only one exception (i.e., A/turkey/Netherlands/543301/1999 was located within H1.1d), and H1 subtype HuIVs were almost exclusively located within H1.2d in the past decades before 2009 and within H1.2c1 after 2009. In contrast, H1 subtype SIVs were quite distinct. For example, many H1 subtype SIVs isolated after 2010 belonged to lineages of H1.1d, H1.2c, H1.2c1, H1.1d, and H1.2d2 (Table 6). Table 6 also demonstrated that the dominant lineages of SIVs circulating in the two hemispheres after 2010 were different from each other.

**Table 6:** Lineage distribution of H1 and H3 subtypes of SIVs after the year 2010

	East Hemisphere	West Hemisphere	Total
H1.1d	289	1	290
H1.2c	162	1136	1298
H1.2c1	133	203	336
H1.2d2a	89		89
H1.2d2b	24		24
H1.2d2c	95	1233	1328
H3.2a	54	256	310
H3.2b1	68		68
H3.2b2	120	1056	1176

The above phylogenetic relationships were largely consistent with our previous study in 2009 which classified H1 subtype IAVs into three primary lineages, h1.1-h1.3 [31]. Of them, h1.1 (AIVs) corresponded to H1.1 in this report, and h1.2 and h1.3 corresponded to H1.2 in this report. Moreover, h1.2 corresponded to H1.2a and H1.2d in this report, and h1.3 corresponded to H1.2b and H1.2c in this report. We put h1.2 and h1.3 into the same primary lineage (i.e., H1.2) in this report because they likely shared the same origin (i.e., H1.2a) and the same host range (humans and pigs), and they were located relatively together in the phylogenetic tree. The sequences used for analysis of phylogenetic relationships were much more in this study than in our previous study in 2009, and consequently, this report exhibited more clades of H1 subtype SIVs than our previous study.

Consistent with our previous study [31], here we did not classify H1 subtype IAVs into H1 subtype AIVs, HuIVs and SIVs. This is because the avian lineage H1.1 also comprised a major group of SIVs (i.e., H1.1c), and the swine lineage H1.2c comprised a major group of HuIVs (i.e., H1.2c1). Additionally, although some groups of IAVs within a lineage of H1 subtype were of significant genetic distances between each other, we did not designate them as subordinate lineages in this report, since these AIVs were of no significant difference with respect to the host, temporal or spatial distribution.

It is interesting that some H1 subtype HuIVs isolated in Iran in recent years, A/Iran/187/2016, A/Iran/279/2016, A/Iran/1417/2016, and A/Shiraz/106/2015 were similar to the HuIVs isolated in the 1930s in lineage H1.2.

### Phylogenetic diversity and distribution of H2 subtype IAVs based on HA sequences

Figure 2 (the thumbnail of the phylogenetic tree) and Additional file 2: Figure SI_2 (the original phylogenetic tree) both suggested that, from a panorama view, H2 subtype IAVs could be classified into two primary lineages, H2.1 and H2.2, based on their HA sequences, largely corresponding to the AIVs isolated from the Western and Eastern Hemispheres, respectively.

H2.2 was more complicated than H2.1, including some HuIVs and AIVs from multiple continents. Two secondary lineages within H2.2, H2.2a and H2.2b, were designated. H2.2a corresponded to H2N2 subtype HuIVs which circulated in the world during the period 1957–1968, indicating that the HA gene of the HuIVs originated from AIVs. H2.2b corresponded to some AIVs from North America. Unlike H1 subtype IAVs, no H2 subtype IAVs have ever been isolated from pigs with only two exceptions from the USA in 2006.

The above phylogenetic relationships were largely consistent with our previous study in 2009 which classified H2 subtype IAVs into two primary lineages, h2.1 and h2.2, which corresponded to H2.1 and H2.2 in this report [31]. Similar to H1 subtype, here we did not designate subordinate lineages in this report for some groups of IAVs which were of significant genetic distances between each other, since these AIVs were of no significant difference with respect to the host, temporal or spatial distribution.

### Phylogenetic diversity and distribution of H3 subtype IAVs based on HA sequences

Figure 3 (the thumbnail of the phylogenetic tree) and Additional file 3: Figure SI_3 (the original phylogenetic tree) both suggested that, from a panorama view, H3 subtype IAVs could be classified into three primary lineages, H3.1, H3.2 and H3.3, based on their HA sequences.

H3.1 corresponded to H3 subtype AIVs, and several secondary lineages within H3.1, H3.1a-H3.1c, were designated. H3.1a corresponded to some AIVs isolated from the Western Hemisphere. H3.1b corresponded to some AIVs isolated from the Pacific. H3.1c corresponded to many AIVs distributed worldwide. Two tertiary lineages within H3.1c were designated: H3.1c1 corresponded to many AIVs isolated from the Western Hemisphere, and H3.1c2 corresponded to some canine or feline IAVs which were similar to some AIVs from South Korea, indicating that these viruses likely originated from the AIVs circulating in South Korea. We did not allocate a subordinate lineage for the groups of AIVs isolated from the Eastern Hemisphere in this study, because these groups were similar to the viruses from the Western Hemisphere (H3.1c1) as compared to H3.1a and H3.1b (Additional file 3: Figure SI_3).

Similar to H1.2d, H3.2 corresponded to many HuIVs and SIVs circulating worldwide. H3.2 covered multiple groups of HuIVs which circulated in humans during the periods from 1968 to nowadays, and multiple groups of SIVs circulating worldwide for decades. Although some groups of HuIVs in H3.2 were inserted by swine groups in the phylogenetic tree (Additional file 3: Figure SI_3), this should be mainly due to that some SIVs were similar to some HuIVs in protein sequences by chance rather than through cross-species transmission. That is to say, cross-species transmission of the viruses in H3.2 did exist, but cross-species transmission has not caused frequent replacement of HuIV lineages by SIV lineages in H3.2. This is because most HuIVs were in human-to-human transmission, and most SIVs were in swine-to-swine transmission, according to their epidemiology. On the other side, the average protein sequence identity of the HuIVs in H3.2 of the same decade after 1980 was around 97%, and its counterpart of the SIVs in H3.2 was around 91%, suggesting that SIVs in H3.2 were of more diversity than HuIVs in H3.2 (*P* < 0.01, by T test), and therefore it was possible that some SIVs were similar to some HuIVs by chance. With these considerations, we designated HuIV groups in H3.2 as H3.2a, and SIV groups in H3.2 as H3.2b, according to their hosts, although these two subordinate lineages inserted into each other in the phylogenetic tree. Nevertheless, the insertion suggested that H3.2a and H3.2b were of the same origin and possible cross-species transmission.

Two subordinate lineages of H3.2b were designated, and H3.2b1 corresponded to a group of SIVs mainly circulating in the Eastern Hemisphere with few exceptions and H3.2b2 corresponded to a group of SIVs mainly circulating in the Western Hemisphere with some exceptions.

H3.3 corresponded to the EIVs circulating from 1963 to nowadays. Some viruses within H3.3 have evolved into a secondary lineage, H3.3a, which mainly circulated in dogs.

As showed in Table 6, H3 subtype SIVs isolated after 2010 belonged to lineages of H3.2a, H3.2b1 and H3.2b2, and H3.2a and H3.2b2 circulated mainly in the Western Hemisphere and H3.2b1 exclusively in the Eastern Hemisphere.

Our previous study in 2009 classified H3 subtype IAVs into three primary lineages, h3.1-h3.3 [31]. Of them, h3.1 corresponded to H3.1 and H3.2 in this report, and h3.2 corresponded to H3.3 in this report. Lineage h3.3 which covered two viruses, A/equine/Argentina/1/2001(H3N8) and A/swine/Quebec/4001/2005(H3N2), disappeared in this report. This is because the sequence of A/swine/Quebec/4001/2005(H3N2) has been changed greatly and consequently this virus was located in the lineage H3.2b2 in this report, and the sequence of A/equine/Argentina/1/2001(H3N8) might be wrong since its nucleotide sequence is quite different from all others (sequence identity < 85%) and its amino acid sequence is quite similar to some others (sequence identity > 99%). Actually, the panorama view of this study was largely consistent with our previous study in 2009 after removing lineage h3.3 which was likely based on false sequences. In addition, two canine lineages (H3.1c2 and H3.3a) and two avian lineages (H3.1a and H3.1b) were added in this report because the related viruses were isolated after our previous study in 2009. One swine lineage (H3.2b2) was also added in this report because this lineage was neglected in our previous study in 2009.

### Phylogenetic diversity and distribution of H4 subtype of IAVs based on HA sequences

Figure 4 (the thumbnail of the phylogenetic tree) and Additional file 4: Figure SI_4 (the original phylogenetic tree) both suggested that, from a panorama view, H4 subtype IAVs could be classified into three primary lineages, H4.1-H4.3.

H4.1 and H4.2 mainly corresponded to AIVs circulating in the North America, and H4.3 corresponded to AIVs from both Hemispheres. Two secondary lineages (H4.3a and H4.3b) within H4.3 were designated, which mainly corresponded to AIVs circulating in the Western and Eastern Hemispheres.

The results of H4 subtype was different with our previous study in 2009 [31]. Our previous study in 2009 which classified H4 subtype IAVs into two primary lineages, h4.1 and h4.2, and h4.1 corresponded to H4.3a in this report, and h4.2 corresponded to H4.3b in this report. The viruses in both H4.1 and H4.2 in this study were either isolated after our previous study in 2009 or neglected by our previous study in 2009.

### Phylogenetic diversity and distribution of H7 and H15 subtypes of IAVs based on HA sequences

Figure 5 (the thumbnail of the phylogenetic tree) and Additional file 5: Figure SI_5 (the original phylogenetic tree) both suggested that, from a panorama view, H7 subtype IAVs could be classified into three primary lineages, H7.1, H7.2 and H7.3, based on their HA sequences.

H7.1 and H7.2 mainly corresponded to H7 subtype AIVs circulating in the Western and the Eastern Hemispheres. H7.3 corresponded to H7 subtype EIVs which have disappeared after the 1970s. Three secondary lineages within H7.2 were designated, and H7.2a mainly corresponded to H7 subtype AIVs circulated in the period from the 1900s to the 1940s, with two surprising exceptions (A/duck/Taiwan/33/1993(H7N7) and A/duck/Taiwan/Ya103/1993(H7N7)). H7.2b corresponded to some H7 subtype AIVs isolated in the Pacific. H7.2c corresponded to H7 subtype AIVs isolated in the Eastern Hemisphere. A tertiary lineage (H7.2c1) within H7.2c were designated, which largely corresponded to the H7N9 subtype AIVs isolated in China which have caused many human infections and fatalities since the year 2013. The H7N9 viruses were designated as A(H7N9)CN2013 below.

H15 subtype IAVs were analyzed along with H7 subtype IAVs because H15 subtype IAVs were of limited sequences and close to H7 subtype IAVs [31]. Figure 5 suggested that H15 subtype IAVs could be classified into two lineages, H15.1 and H15.2. H15.1 corresponded to some AIVs isolated in Australia in the 1970s and the 1980s. H15.2 corresponded to a few AIVs isolated from Russia around the year 2010.

The above phylogenetic relationships were largely consistent with our previous study in 2009 [31], except for the addition of lineage H15.2. Some H7 subtype AIVs without distinct distribution have not been designated as subordinate lineages, and on the other side, we did designate A(H7N9)CN2013 as a subordinate lineage, because this designation is needed to facilitate communication about the viruses of great biomedical significance.

### Phylogenetic diversity and distribution of H9 subtype IAVs based on HA sequences

Figure 6 (the thumbnail of the phylogenetic tree) and Additional file 6: Figure SI_6 (the original phylogenetic tree) both suggested that, from a panorama view, H9 subtype IAVs could be classified into two primary lineages, H9.1 and H9.2, based on their HA sequences.

H9.1 corresponded to some H9 subtype AIVs circulating worldwide, and most of the AIVs within H9.1 were isolated from wild or domestic water fowls. Some clades within H9.1, such as those represented by A/duck/Malaysia/20/1998(H9N2) or A/Chicken/Korea/MS96/96(H9N2), corresponded to some AIVs circulating in a country or region, while some other clades, such as those represented by A/ruddy turnstone/Delaware/AI03–224/2003(H9N9) or A/duck/Hong Kong/644/79(H9N2), corresponded to some AIVs circulating worldwide.

H9.2 corresponded to many AIVs circulating in the Eastern Hemisphere. Most of the AIVs within H9.2 were isolated from chickens. Two secondary lineages within H9.2, H9.2a and H9.2b, largely corresponded to the AIVs circulating in the region around the West and South Asia and the AIVs circulating in China.

It is surprising that one H9 subtype SIV isolated in Yantai in recent years, A/swine/Yantai/16/2012(H9N2), was similar to the AIV of A/turkey/Wisconsin/1/1966(H9N2) isolated in the 1960s in the lineage H9.1.

The above phylogenetic relationships were different with our previous study in 2009 which classified H9 subtype IAVs into four primary lineages, h9.1-h9.4 [31]. Of them, h9.1, h9.2 and H9.3 constituted H9.1 in this report, and h9.4 was equal to H9.2 in this report. The lineages h9.1 and h9.2 were located together with h9.3 in this study possibly because more nucleotide substitutions of the viruses within h9.1 and h9.2 led to synonymous mutations, as compared with other lineages. Three secondary lineages were designated within the primary lineage h9.3 in our previous study, while no secondary lineages were designated for these viruses in this study, as in this report we paid less attention to genetic distance in the classification. Secondary lineages in the lineage h9.4 were designated in the same way as H9.2 in this study.

### Phylogenetic diversity and distribution of H5-H6, H8, H10-H12, H14, and H16 subtypes of IAVs based on HA sequences

Figures 7-14 (the thumbnail of the phylogenetic tree) and Additional file 7: Figure SI_7, Additional file 8: Figure SI_8, Additional file 9: Figure SI_9, Additional file 10: Figure SI_10, Additional file 11: Figure SI_11, Additional file 12: Figure SI_12, Additional file 13: Figure SI_13 and Additional file 14: Figure SI_14 (the original phylogenetic tree) suggested that, from a panorama view, H5 subtype IAVs could be classified into two primary lineages, which mainly corresponded to AIVs circulating in the Western and Eastern Hemispheres, and this was similar for H6, H8, H10-H12, H14, and H16 subtypes of IAVs.

A secondary lineage (H5.1a) within H5.1 was designated because it corresponded to some strains mainly from Taiwan after 2012, and a secondary lineage (H5.2a) within H5.2 was designated because it was of distinct epidemiological significance as it corresponded to the H5 subtype HPAIVs widely circulating in the world in recent years. Multiple hierarchies of clades within H5.2a, such as clades 2, 2.3, 2.3.2, 2.3.2.1, and 2.3.2.1c, have been classified by an ad hoc expert group [18], and these clades have not been classified in this report because we paid more attention to major lineages of IAVs.

We designated two secondary lineages (H11.2a and H1.2b) within H11.2 which corresponded to some AIVs isolated from North America and the Antarctic, and one secondary lineage (H6.1a) within H6.1 which corresponded to many AIVs from both Hemispheres.

The results of the subtypes H5-H6, and H10-H12 were similar to those of our previous study in 2009 with minor revision [31]. Our previous study in 2009 which classified H5 subtype IAVs into three primary lineages, h5.1-h5.3 [31], and h5.1 corresponded to H5.1 in this study, and h5.2 and h5.3 corresponded to H5.2 in this study. Additionally, only one group of H5 subtype AIVs was designated as a subordinate lineage because of its distinct epidemiological significance rather than genetic distances, while multiple groups of AIVs were designated as subordinate lineages in our previous study in 2009. H8, H14 and H16 could not be classified into some primary lineages in our previous panorama study in 2009, and they were all classified into primary lineages mainly because more sequences have been available in GenBank after 2009.

### Phylogenetic diversity and distribution of H13 subtypes of IAVs based on HA sequences

Figure 15 (the thumbnail of the phylogenetic tree) and Additional file 15: Figure SI_15 (the original phylogenetic tree) both suggested that, from a panorama view, H13 subtype IAVs could be classified into two global primary lineages for H13 subtype. Each of them was classified into two secondary lineages, which mainly corresponded to the AIVs circulating in the Western Hemisphere (H13.1a and H13.2a) and the Eastern Hemisphere (H13.1b and H13.2b).

The results of the subtype H13 were similar to those of our previous study in 2009 with minor revision [31]. Our previous study in 2009 classified H13 subtype IAVs into three primary lineages, h13.1-h13.3. The previous primary lineages h13.1 and h13.3 constituted H13.1 in this report, and h13.2 was equal to H13.2 in this report which was further classified into two secondary lineages mainly because novel sequences have been available in GenBank after 2009.

### Phylogenetic diversity and distribution of H17 and H18 subtypes of IAVs based on HA sequences

We constructed the phylogenetic tree covering some sequences of H1-H16 subtypes and the strains of H17 and H18 subtypes based on their HA gene sequences as shown in Fig. 16 (the original phylogenetic tree). The phylogenetic tree suggested that the two bat subtypes (H17 and H18) were quite distinct in their sequences as compared with H1-H16 subtypes, and thus we proposed or supported such a hypothesis is that there should be more subtypes of IAVs in bats in the world.

#### Phylogenetic diversity and distribution of N1 subtype IAVs based on NA sequences

Figure 17 (the thumbnail of the phylogenetic tree) and Additional file 16: Figure SI_16 (the original phylogenetic tree) both suggested that, from a panorama view, N1 subtype IAVs could be classified into three lineages, N1.1, N1.2 and N1.3, largely corresponding to avian, human and classical swine N1 subtype IAVs.

Three secondary lineages within N1.1 were designated, and N1.1a corresponded to a few of AIVs circulating in the 1930s, and N1.1b corresponded to some AIVs circulating in the Western Hemisphere, and N1.1c corresponded to many AIVs circulating in the Western Hemisphere (N1.1c1) and many AIVs circulating in the Eastern Hemisphere (N1.1c2).

Two quaternary lineages within N1.1c2 were designated, and N1.1c2a corresponded to the H5N1 subtype HPAIVs widely circulating in the Eastern Hemisphere in recent years, and N1.1c2b corresponded to the H1 subtype Eurasian avian-like SIVs whose HA genes were located in H1.1d. One subordinate lineage (N1.1c2b1) within N1.1c2b corresponded to A(H1N1)pdm09.

N1.2 mainly corresponded to seasonal H1N1 subtype HuIVs circulating worldwide in humans before 2010. Four secondary lineages within N1.2 were further designated according to their circulation years. N1.2a corresponded to an H1N1 subtype HuIV isolated in 1918, and N1.2b corresponded to H1N1 subtype HuIVs circulating during the period 1933–1947, and N1.2c mainly corresponded to H1N1 subtype HuIVs circulating during the period 1948–1984, except A/Neimenggu/63/1997(H1N1) and A/swine/Tianjin/01/2004(H1N1), and N1.2d corresponded to H1N1 subtype HuIVs circulating during the period 1986–2009.

N1.3 corresponded to classical H1N1 SIVs circulating worldwide. Two secondary lineages within N1.3 were further designated, N1.3a and N1.3b. N1.3a corresponded to SIVs mainly isolated in the 1930s and the 1940s with two exceptions isolated in 1973 and 2009, respectively. N1.3b corresponded to some SIVs circulating during the period 1985–2016.

The above phylogenetic relationships were largely consistent with our previous study in 2009 which classified N1 subtype IAVs into three primary lineages, n1.1-n1.3 [31], which are equal to N1.1-N1.3 in this report. The secondary lineages of N1.1 (AIVs) was fewer than those of n1.1, but the level of subordinate lineages of N1.1 was more than that of n1.1 and a new lineage (N1.1c2b1) was added, due to different criteria for classification of subordinate lineages.

### Phylogenetic diversity and distribution of N2 subtype IAVs based on NA sequences

Figure 18 (the thumbnail of the phylogenetic tree) and Additional file 17: Figure SI_17 (the original phylogenetic tree) both suggested that, from a panorama view, N2 subtype IAVs could be classified into two primary lineages, N2.1 and N2.2, largely corresponding to avian and human/swine IAVs.

Two secondary lineages within N2.1 were designated as N2.1a (corresponding to many AIVs circulating worldwide) and N2.1b (corresponding to many H9N2 subtype AIVs circulating in Asia after 1994); Multiple tertiary lineages within N2.1a were designated. Among them, N2.1a1-N2.1a2 corresponded to two groups of AIVs mainly isolated from the Eastern Hemisphere, and N2.1a3 corresponded to AIVs distributed worldwide, and N2.1a4 corresponded to the H2N2 subtype HuIVs circulating worldwide during the period 1957–1965. One quaternary lineage (N2.1a3a) within N2.1a3 was designated, which corresponded to H3N2 subtype CIVs and feline IAVs after the 2000s.

N2.2 mainly corresponded to HuIVs and SIVs circulating worldwide. The groups of HuIVs were inserted by multiple groups of highly diversified SIVs within the phylogenetic tree (Additional file 17: Fig SI_17), making it difficult to designate subordinate lineages of N2.2. The SIVs within N2.2 were of so great diversity that it was unable to conclude any cross-species transmission between the HuIVs and SIVs in N2.2. Considering the fact that the NA genes of most of the HuIVs within N2.2 were likely not from the SIVs, we designated five subordinate lineages for the HuIVs within N2.2, N2.2a-N2.2e. N2.2a largely corresponded to the H2N2 subtype HuIVs circulating during the period 1957–1968. N2.2b largely corresponded to some H3N2 subtype HuIVs circulating in the 1970s. N2.2c largely corresponded to some H3N2 subtype HuIVs circulating in the 1980s. N2.2d largely corresponded to some H3N2 subtype HuIVs circulating in the 1990s. N2.2e largely corresponded to some H3N2 subtype HuIVs circulating after 2000. The SIVs in N2.2 were classified into two subordinate lineages, N2.2f and N2.2g. N2.2f corresponded to SIVs in Japan during the period 1980–2012. N2.2g corresponded to multiple groups of SIVs interrupted by HuIVs in the phylogenetic tree (Additional file 17: Figure SI_17).

The above phylogenetic relationships were largely consistent with our previous study in 2009 which classified N2 subtype IAVs into two primary lineages, n2.1 and n2.2 [31], which were equal to N2.1 and N2.2 in this report. N2.1 (AIVs) was given less secondary lineages in this report than n2.1 in the previous report, but N2.1 was given more tertiary lineages than n2.1, with the effect to better exhibit the phylogenetic relationships of the viruses. N2.2 (HuIVs and SIVs) was given more secondary lineages in this report than n2.2 in the previous study.

Our previous study in 2009 identified that the nucleotide sequence of the NA gene of A/swine/Quebec/4001/2005(H3N2) was quite distinct from its counterparts of all other IAVs. Now the gene sequence of the strain has been greatly changed in GenBank and was similar to those of other SIVs in the lineage N2.2 isolated from North America.

### Phylogenetic diversity and distribution of N4-N6 subtypes of IAVs based on NA sequences

Figures 19-21 (the thumbnail of the phylogenetic tree) and Additional file 18: Figure SI_18, Additional file 19: Figure SI_19 and Additional file 20: Figure SI_20 (the original phylogenetic tree) suggested that, from a panorama view, N4, N5 and N6 subtypes of IAVs all could be classified into two primary lineages, which mainly corresponded to the AIVs circulating in the Western and Eastern Hemispheres. Additionally, one secondary lineage was designated within the primary lineage, N4.2a, N5.2a and N6.2a, which all corresponded to AIVs isolated from the Pacific. Two other secondary lineages were designated within N6.2, N6.2b and N6.2c, which both corresponded to H5N6 subtype HPAIVs.

The above phylogenetic relationships of N4-N6 subtypes were largely consistent with our previous study in 2009 which also classified these subtypes into two similar primary lineages [31]. However, our previous study in 2009 did not designate any secondary lineages for the primary lineages, and this report designated one to three secondary lineages for them due to their spatial distribution or biomedical significance.

### Phylogenetic diversity and distribution of N3 and N9 subtypes of IAVs based on NA sequences

Figures 22-23 (the thumbnail of the phylogenetic tree) and Additional files 21-22: Figures SI_21–22 (the original phylogenetic tree) suggested that, from a panorama view, N3 and N9 subtypes of IAVs could be classified into two primary lineages. Three secondary lineages within N3.1 were designated, and they largely corresponded to the AIVs circulating in North America, South America and the Eastern Hemisphere, respectively. Like N3.1, three secondary lineages within N3.2 were designated, and N3.2a and N3.2b largely corresponded to the AIVs circulating in North America, and N3.2c largely corresponded to the AIVs circulating in the Eastern Hemisphere. Two secondary lineages were designated within N9.1 and N9.2 corresponding to the AIVs from the Western and Eastern Hemispheres, respectively. A tertiary lineage within N9.1b designated as N9.1b1 corresponded to A(H7N9)CN2013.

The above phylogenetic relationships were largely consistent with our previous study in 2009 which classified the two NA subtypes into two similar primary lineages [31]. However, our previous study in 2009 did not designate any secondary lineages for the primary lineages of n3.2 and n9.2, and this report designated two or three secondary lineages for them according to their spatial distribution. Moreover, one tertiary lineage emerging after 2009 (N9.1b1) was designated in this study due to its great significance in public health.

### Phylogenetic diversity and distribution of N7 and N8 subtypes of IAVs based on NA sequences

Figures 24-25 (the thumbnail of the phylogenetic tree) and Additional files 23-24: Figures SI_23–24 (the original phylogenetic tree) all suggested that, from a panorama view, N7 and N8 subtypes of IAVs could be classified into three primary lineages. N7.1 and N8.1 corresponded to AIVs circulating in the Western Hemisphere; N7.2 and N8.2 corresponded to AIVs circulating in the Eastern Hemisphere; N7.3 and N8.3 corresponded to EIVs. Additionally, a secondary lineage (N8.2a) within N8.2 corresponded to H5N8 subtype HPAIVs, and two secondary lineages (N8.3a and N8.3b) within N8.3 corresponded to H3N8 subtype CIVs.

The above phylogenetic relationships of N7 and N8 were largely consistent with our previous study in 2009 which classified N7 and N8 subtype IAVs into three primary lineages, the primary lineages were equal to the corresponding primary lineages in this study [31]. Unlike to the previous study in 2009, no secondary lineages were designated within N7.2 in this study. Meanwhile, more secondary lineages of N8.2 (AIVs) and N8.3 (EIVs) were designated than those of n8.2 and n8.3 because two secondary lineages (N8.3a and N8.3b) of H3N8 subtype CIVs emerged after 2009.

### Phylogenetic diversity and distribution of N10 and N11 subtypes of IAVs based on NA sequences

We constructed the phylogenetic tree covering some sequences of N1-N9 subtypes and the strains of N10 and N11 subtypes based on their NA gene sequences, as shown in Fig. 26 (the original phylogenetic tree). The phylogenetic relationships among the strains of N1-N9 subtypes revealed by this tree were consistent with our previous report in 2009 [31]. Similar to the H17 and H18 subtypes, the two bat subtypes (N10 and N11) were quite distinct in their sequences as compared with N1-N9 subtypes, and thus we proposed or supported such a hypothesis that there should be more subtypes of IAVs in bats in the world.

### Phylogenetic diversity and distribution of IAVs based on PB2 sequences

Figure 27 (the thumbnail of the phylogenetic tree) and Additional file 25: Figure SI_25 (the original phylogenetic tree) suggested that, based on 17,063 protein sequences of the viral PB2 gene, IAVs could be classified into four primary lineages, PB2.1, PB2.2, PB2.3, and PB2.4 which mainly corresponded to AIVs, HuIVs, SIVs, and EIVs.

PB2.1 corresponded to AIVs circulating worldwide. Four cross-species lineages within PB2.1 were designated as secondary lineages PB2.1a-PB2.1d. PB2.1a corresponded to H1 subtype Eurasian avian-like SIVs from the 1980s. PB2.1b corresponded to the SIVs which emerged in the 1990s in North American, and the tertiary lineage PB2.1b1 corresponded to A(H1N1)pdm09, and one quaternary lineage (PB2.1b1a) within PB2.1b1 corresponded to some CIVs which were assumed to be derived from the HuIVs within PB2.1b1. PB2.1c corresponded to H3N8 and H7N7 subtypes of EIVs circulating worldwide. A tertiary lineage PB2.1c1 within PB2.1c corresponded to H3N8 subtype CIVs. PB2.1d corresponded to H3N2 subtype CIVs emerged in recent years.

PB2.2 corresponded to classical HuIVs including H1N1 subtype HuIVs mainly circulating before 2010, H2N2 subtype HuIVs circulating in the 1950s and the 1960s, and H3N2 subtype HuIVs circulating from 1968. PB2.3 corresponded to classical SIVs circulating from the 1930s to the 2000s and the ancient HuIV strain of A/Brevig Mission/1/1918(H1N1). PB2.4 corresponded to H7N7 subtype EIVs circulating in the 1950s and the 1960s.

The equine lineage PB2.1c was subordinate to PB2.1 because the viruses within PB2.1c were close to some AIVs within PB2.1. This is different from the equine lineage PB2.4 as all the viruses within PB2.4 were distant from all the AIVs within PB2.1 (Additional file 25: Figure SI_25).

Our previous study in 2009 classified the viral PB2 gene into eight primary lineages, S1.1-S1.8 [32]. Of them, the avian lineages S1.1, S1.2 and S1.7 corresponded to PB2.1 in this report, and the human lineage S1.3 corresponded to PB2.2 in this report, and the swine lineage S1.4 corresponded to PB2.3 in this report. The equine lineage S1.6 corresponded to PB2.4 in this report, and the other equine lineage S1.5 corresponded to the secondary lineage PB2.1c in this report. The viruses within S1.7 were located in PB2.1 in this study possibly because multiple nucleotide substitutions of the viruses within S1.7 led to synonymous mutations. S1.8 included two viruses, A/mink/Nova-Scotia/1055488/2007(H3N2) and A/swine/Quebec/4001/2005(H3N2), and this lineage disappeared in this report because the nucleic acid sequences of the two viruses have been revised greatly, and now these two viruses were assigned into PB2.1b. Actually, the panorama view of this study was largely consistent with our previous study in 2009 after removing the lineages of S1.7 and S1.8. In addition, three canine lineages (PB2.1d, PB2.1b1a and PB2.1c1) were added in this report, and the related viruses were isolated after our previous study in 2009 or neglected by our previous study in 2009. Similarly, some other subordinate lineages, such as PB2.1b1 were added in this report because the related viruses were isolated after our previous study in 2009.

We did not classify the human lineage PB2.2 into any secondary lineages because it is difficult to assign some intermediate strains to a secondary lineage. Actually, we selected some strains within PB2.2 to represent some secondary lineages within PB2.2. Some other subordinate lineages of PB2 were also represented by strains in the same way and for the same reason.

### Phylogenetic diversity and distribution of IAVs based on PB1 sequences

Figure 28 (the thumbnail of the phylogenetic tree) and Additional file 26: Figure SI_26 (the original phylogenetic tree) suggested that, based on 16,009 protein sequences of the viral PB1 gene, IAVs could be classified into four primary lineages, PB1.1, PB1.2, PB1.3, and PB1.4 which mainly corresponded to AIVs, HuIVs, SIVs, and EIVs.

PB1.1 corresponded to AIVs circulating worldwide. Nine cross-species lineages within PB1.1 were designated as secondary lineages PB1.1a-PB1.1i. PB1.1a corresponded to H2N2 subtype HuIVs circulating during the period 1957–1968. PB1.1b corresponded to H3N2 subtype HuIVs circulating from the 1960s to the 2000s. PB1.1c corresponded to H3N2 subtype HuIVs circulating after 1990 except one H1N1 subtype HuIV. PB1.1d corresponded to A(H1N1)pdm09 and some H1N2 or H3N2 subtypes of HuIVs. The tertiary lineage PB1.1d1 within PB1.1d corresponded to H1N1 subtype CIVs which were assumed to be from HuIVs. PB1.1e corresponded to H1 subtype Eurasian avian-like SIVs circulating from the 1970s. PB1.1f corresponded to H3N2 and H1N2 subtypes of SIVs circulating from 1990. PB1.1 g corresponded to the SIVs which emerged in the 1990s in North America. PB1.1 h corresponded to H3N8 and H7N7 subtype EIVs circulating from the 1960s to the 2000s. A tertiary lineage PB1.1 h1 within PB1.1 h corresponded to H3N8 subtype CIVs. PB1.1i corresponded to H3N2 subtype CIVs which emerged in recent years.

PB1.2 corresponded to classical HuIVs including H1N1 subtype HuIVs mainly circulating before 2009. PB1.3 corresponded to classical SIVs circulated worldwide from the 1930s to the 2000s. PB1.4 corresponded to H7N7 subtype EIVs isolated in the 1950s and the 1960s.

The equine lineage PB1.1 h was subordinate to PB1.1 because the viruses within PB1.1 h were close to some AIVs within PB1.1. This is different from the equine lineage PB1.4 as all the viruses within PB1.4 were distant from all the AIVs within PB1.1 (Additional file 26: Figure SI_26).

Our previous study in 2009 classified the viral PB1 gene into eight primary lineages, S2.1-S2.8 [32]. Of them, S2.1, S2.2 and most of the isolates of S2.6 (AIVs) corresponded to PB1.1 in this report, and S2.3 corresponded to PB1.2 in this report, and S2.4 corresponded to PB1.3 in this report, and S2.5 corresponded to the secondary lineage PB1.1 h in this report, and S2.8 corresponded to PB2.4 in this report. S2.7 including two viruses, A/mink/Nova Scotia/1055488/2007(H3N2) and A/swine/Quebec/4001/2005(H3N2), disappeared in this report because their nucleic acid sequences have been revised greatly, and now the two viruses were assigned into PB2.1 g. Actually, the panorama view of this study was largely consistent with our previous study in 2009 after removing the lineage S2.7. In addition, two canine lineages (PB1.1 h1 and PB1.1i) were added in this report, and the related viruses were isolated after our previous study in 2009 or neglected by our previous study in 2009. Similarly, some other subordinate lineages, such as PB1.1d were added in this report because the related viruses were isolated after our previous study in 2009.

### Phylogenetic diversity and distribution of IAVs based on PA sequences

Figure 29 (the thumbnail of the phylogenetic tree) and Additional file 27: Fig SI_27 (the original phylogenetic tree) suggested that, based on 17,494 protein sequences of the viral PA gene, IAVs could be classified into four primary lineages, PA.1, PA.2, PA.3, and PA.4 which mainly corresponded to AIVs, HuIVs, SIVs, and EIVs.

Four cross-species lineages within PA.1 were designated as secondary lineages PA.1a-PA.1d. PA.1a corresponded to H1 subtype Eurasian avian-like SIVs circulating after 1979. PA.1b corresponded to SIVs which emerged in the 1990s in North American, and the tertiary lineage PA.1b1 within PA.1b corresponded to A(H1N1)pdm09, and one quaternary lineages (PA.1b1a) within PA.1b1 was H1N1 subtype CIVs. PA.1c corresponded to H3N8 and H7N7 subtypes of EIVs circulating worldwide. A tertiary lineage PA.1c1 within PA.1c corresponded to H3N8 subtype CIVs. PA.1d corresponded to H3N2 subtype CIVs emerged in recent years.

Six secondary lineages (PA.2a-PA.2f) were designated within PA.2. PA.2a corresponded to one H1N1 subtype HuIV in 1918. PA.2b corresponded to H1N1 subtype HuIVs circulating in the 1930s and the 1940s. Two HuIVs isolated in 1989 and 2015, A/Victoria/1/1989(H3N2) and A/Tehran/Sm05/2015(H1N1), were unexpectedly covered in PA.2b. PA.2c corresponded to H1N1 subtype HuIVs circulating in the 1940s. PA.2d corresponded to H1N1 subtype HuIVs circulating during the period 1948–2009 and H2N2 subtype HuIVs circulating during the period 1957–1968. PA.2e corresponded to multiple subtypes of HuIVs mainly circulating in the 1960s and the 1970s. PA.2f corresponded to H3N2 subtype HuIVs circulating after 1968.

Two secondary lineages (PA.3a and PA.3b) were designated within PA.3 according to circulating time. PA.3a corresponded to H1N1 subtype SIVs circulating in the 1930s and 1940s, except A/swine/Guangdong/L3/2009(H1N1). PA.3b mainly corresponded to H1N1 subtype SIVs circulating after 1950 and H1N2 subtype SIVs circulating after 1996, except A/swine/Tainan/103–11/2003(H3N1).

PA.4 corresponded to H7N7 subtype EIVs isolated in the 1950s and the 1960s. The equine lineage PA.1c was subordinate to PA.1 because the viruses within PA.1c were close to some AIVs within PA.1. This is different from the equine lineage PA.4 as all the viruses within PA.4 were distant from all the AIVs within PA.1 (Additional file 27: Fig SI_27).

Our previous study in 2009 classified the viral PA gene into nine primary lineages, S3.1-S3.9 [32]. Of them, the avian lineages S3.1, S3.2, S3.6, and S3.7 corresponded to PA.1 in this report, and the human lineage S3.3 corresponded to PA.2 in this report, and the swine lineage S3.4 corresponded to PA.3 in this report. The equine lineages S3.9 and S3.5 corresponded to PA.4 and the secondary lineage PA.1c in this report, respectively. The lineage S3.8 including two viruses, A/mink/Nova-Scotia/1055488/2007(H3N2) and A/swine/Quebec/4001/2005(H3N2), disappeared in this report because their nucleic acid sequences of the two viruses have been revised greatly, and now these two viruses were assigned into PA.1b. Actually, the panorama view of this study was largely consistent with our previous study in 2009 after removing the lineage S3.8. In addition, three canine lineages (PA.1b1a, PA.1c1 and PA.1d) were added in this report, and the related viruses were isolated after our previous study in 2009 or neglected by our previous study in 2009. Similarly, some other subordinate lineages, such as PA.1b1 were added in this report because the related viruses were isolated after our previous study in 2009.

### Phylogenetic diversity and distribution of IAVs based on NP sequences

Figure 30 (the thumbnail of the phylogenetic tree) and Additional file 28: Fig SI_28 (the original phylogenetic tree) suggested that, based on 10,470 protein sequences of the viral NP gene, IAVs could be classified into three primary lineages, NP.1, NP.2 and NP.3 which mainly corresponded to AIVs, HuIVs and SIVs.

NP.1 corresponded to AIVs circulating worldwide. Four cross-species lineages within NP.1 were designated as secondary lineages NP.1a-NP.1d. NP.1a corresponded to H1 subtype Eurasian avian-like SIVs from the 1980s. NP.1b corresponded to H7N7 subtype EIVs isolated in 1950s–1960s. NP.1c corresponded to H3N8 and H7N7 subtypes of EIVs circulating worldwide. Two tertiary lineages NP.1c1 and NP.1c2 within NP.1b corresponded to H3N8 subtype CIVs. NP.1d corresponded to H3N2 subtype CIVs similar to some AIVs from Korea and China emerged in recent years.

NP.2 corresponded to classical HuIVs, six secondary lineages were designated (NP.2a-NP.2f) within NP.2 according to circulating time and subtype, NP.2a-NP.2e were HuIV lineages. NP.2a corresponded to H1N1 subtype HuIV in 1918. NP.2b corresponded to H1N1 subtype HuIVs circulating in 1930s–1940s, except few exceptions (A/Victoria/JY2/1968(H3N2), A/Mongolia/111/91(H1N1), A/Mongolia/153/88(H1N1), A/Mongolia/231/85(H1N1), A/Nanchang/08/2010(H1N1), and A/swine/North Carolina/B58223/2014(H1N1)). NP.2c corresponded to H1N1 subtype HuIVs circulating in 1980–2009. NP.2d mainly corresponded to H2N2 subtype HuIVs in 1957–1968 and H3N2 subtype HuIVs in 1968–1984, except A/PAL/unknown(H1N2). NP.2e corresponded to H3N2 subtype HuIVs after the 1970s. NP.2f corresponded to 1 H7N9 subtype AIV (A/duck/Zhejiang/LS02/2014(H7N9)) and 1 H3N2 subtype SIV (A/swine/Jilin/19/2007(H3N2)).

NP.3 corresponded to SIVs circulating from the 1930s, three secondary lineages (NP.3a, NP.3b and NP.3c) were designated within NP.3. NP.3a mainly corresponded to H1N1 subtype SIVs circulating in 1930s-1949, NP.3b corresponded to H1N1 subtype SIVs circulating in 1930s and 1954, NP.3c corresponded to H1N1, H1N2 and H3N2 subtype SIVs circulating worldwide after 1957. Two tertiary lineages NP.3c1 and NP.3c2 within NP.3c corresponded to A(H1N1)pdm09.

It is interesting that NP.2f including A/duck/Zhejiang/LS02/2014(H7N9) and A/swine/Jilin/19/2007(H3N2), which was distinct with other viruses in NP.2. We found these two viruses were with high homology with HuIVs by BLAST in NCBI.

Our previous study in 2009 classified the viral NP gene into ten primary lineages, S5.1-S5.10 [32]. Of them, S5.1, S5.2, S5.5.1, S5.6, S5.7, and S5.9 (AIVs) corresponded to NP.1 in this report, and S5.3 (HuIVs) corresponded to NP.2 in this report, and S5.4 (SIVs) corresponded to NP.3 in this report. S5.5.2 (EIVs) corresponded to the secondary lineage NP.1c in this report, and S5.8 (EIVs) corresponded to NP.1b in this report. S5.9 disappeared in this report because the strain A/northern pintail/California/44291–259/2007(H10N3) in S5.9 was absent in GenBank and the other strain within S5.9 (A/duck/LA/17G/1987(H3N8)) was now assigned into NP.1 possibly because more nucleotide substitutions of the virus led to synonymous mutations as compared with other strains. The lineage S5.10 covering two viruses, A/mink/Nova-Scotia/1055488/2007(H3N2) and A/swine/Quebec/4001/2005(H3N2), disappeared in this report because the nucleic acid sequences of these two viruses have been revised greatly, and now the two viruses were assigned into NP.3c. Actually, the panorama view of this study was largely consistent with our previous study in 2009 after removing the lineages S 5.9 and S5.10. In addition, three canine lineages (NP.1c1, NP.1c2 and NP.1d) were added in this report, and the related viruses were isolated after our previous study in 2009 or neglected by our previous study in 2009. Similarly, some other subordinate lineages, such as NP.3c1 and NP.3c2 were added in this report because the related viruses were isolated after our previous study in 2009.

### Phylogenetic diversity and distribution of IAVs based on MP sequences

Figure 31 (the thumbnail of the phylogenetic tree) and Additional file 29: Fig SI_29 (the original phylogenetic tree) suggested that, based on 4194 protein sequences of the viral MP gene, IAVs could be classified into three primary lineages, MP.1, MP.2 and MP.3 which mainly corresponded to AIVs, HuIVs and EIVs.

Six cross-species lineages within MP.1 were designated as secondary lineages MP.1a-MP.1e. MP.1a corresponded to classical SIVs circulating from the 1930s to 2000s. MP.1b corresponded to the SIVs which emerged in the 1990s in North American, and three tertiary lineages, MP.1b1-MP.1b3, within MP.1b all corresponded to A(H1N1)pdm09. MP.1c corresponded to H3N8 subtype EIVs (except three avian isolates in this lineage) circulating worldwide. A tertiary lineage MP.1c1 corresponded to H3N8 subtype CIVs. MP.1d and MP.1e corresponded to H3N2 subtype CIVs emerged in recent years.

Two secondary lineages (MP.2a and MP.2b) were designated within MP.2. MP.2a mainly corresponded to H1N1 subtype HuIVs circulating from the 1930s to 2009, except few isolates of H2N2 subtype in the 1960s and H3N2 subtype in the 2000s. MP.2b mainly corresponded to H3N2 subtype HuIVs circulating from the 1930s to the 2010s, except a few isolates of H2N2 subtype HuIVs circulating from 1957 to 1968, some H1N1 subtype HuIVs circulating from 1943 to 1951 and one HuIV A/Helsinki/743 M/2014(H1N1).

The equine lineage MP.1c and MP.1d were subordinate to MP.1 because the viruses within MP.1c and MP.1d were close to some AIVs within MP.1. This was different from the equine lineage MP.3 as all the viruses within MP.3 were distant from all the AIVs within MP.1 (Additional file 29: Fig SI_29).

Our previous study in 2009 classified the viral MP gene into six primary lineages, S7.1-S7.8 [32]. Of them, S7.1 and S7.2 (AIVs) corresponded to MP.1 in this report, and S7.3 (HuIVs) corresponded to MP.2 in this report, and S7.6 (EIVs) corresponded to MP.3 in this report. S7.4 (SIVs) corresponded to the secondary lineage MP.1a, and S7.5 corresponded to the secondary lineages of MP.1c and MP.1d. In general, the panorama view of this study was largely consistent with our previous study in 2009. Additionally, three canine lineages (MP.1d1, MP.1e and MP.1f) were added in this report, and the related viruses were isolated after our previous study in 2009 or neglected by our previous study in 2009. Similarly, some other subordinate lineages, such as MP.1b1-MP.1b3 were added in this report because the related viruses were isolated after our previous study in 2009.

### Phylogenetic diversity and distribution of IAVs based on NS sequences

Figure 32 (the thumbnail of the phylogenetic tree) and Additional file 30: Fig SI_30 (the original phylogenetic tree) suggested that, based on 14,550 protein sequences of the viral NS gene, IAVs could be classified into three primary lineages, NS.1, NS.2 and NS.3.

Both NS.1 and NS.2 corresponded to AIVs circulating worldwide. Five cross-species lineages within NS.1 were designated as secondary lineages NS.1a-NS.1e. NS.1a corresponded to classical HuIVs, and four tertiary lineages (NS.1a1-NS.1a4) were designated within NS.1a. NS.1a1 corresponded to one H1N1 subtype HuIV in 1918; NS.1a2 corresponded to H1N1 subtype HuIVs circulating in the 1930s and the 1940s, except two HuIVs (A/KOL/536/2007(H1N1), A/Tehran/Sm05/2015(H1N1)) and one SIV (A/swine/KU/8/2001(H1N1)); NS.1a3 corresponded to H1N1 subtype HuIVs circulating during the period 1949–2009 and some H2N2 subtype HuIVs circulating during the period 1957–1968 and one H3N2 subtype HuIV A/Caen/1/1984(H3N2); NS.1a4 mainly corresponded to H3N2 subtype HuIVs after 1968 and some H2N2 subtype HuIVs circulating in the 1960s and two H1N1 subtype HuIVs (A/Uganda/MUWRP-092/2009(H1N1) and A/Singapore/66 L/2007(H1N1)). NS.1b corresponded to classical SIVs circulating from the 1930s, and two tertiary lineages (NS.1b1 and NS.1b2) were designated within NS.1b. NS.1b1 corresponded to A(H1N1)pdm09, and NS.1b2 corresponded to H1N1 subtype CIVs circulating after 2013. NS.1c corresponded to H1 subtype Eurasian avian-like SIVs from the 1970s. NS.1d corresponded to H3N8 subtype EIVs and a few strains isolated from other hosts, such as A/African starling/England-Q/983/1979(H7N1), A/swine/Anhui/01/2006(H3N8) and A/donkey/Shandong/1/2017(H3N8). Two tertiary lineages NS.1d1 and NS.1d2 within NS.1d corresponded to H3N8 subtype CIVs. NS.1e corresponded to H3N2 subtype CIVs which emerged in recent years similar to some AIVs from Korea.

NS.2 corresponded to another group of AIVs circulating worldwide which were quite distinct from NS.1. NS.3 corresponded to H7N7 subtype EIVs isolated from the 1950s to the 1970s.

The equine lineage NS.1d was subordinate to NS.1 because the viruses within NS.1d were close to some AIVs within NS.1. This is different from the equine lineage NS.3 as all the viruses within NS.3 were distant from all the AIVs within NS.1 (Additional file 30: Fig SI_30).

Our previous study in 2009 classified the viral NS gene into ten primary lineages, S8.1-S8.10 [32]. Of them, S8.1, S8.2, S8.7, S8.8, and S8.9 (AIVs) corresponded to NS.1 in this report, and S8.6 (AIVs) corresponded to primary lineage NS.2 in this report, and S8.10 (EIVs) corresponded to NS.3 in this report. S8.3 (HuIVs) corresponded to secondary lineage NS.1a in this report; S8.4 (SIVs) corresponded to secondary lineage NS.1b in this study; S8.5 corresponded to secondary lineage NS.1d. In general, the panorama view of this study was largely consistent with our previous study in 2009. Four canine lineages (NS.1b2, NS.1d1, NS.1d2, and NS.1e) were added in this report, and the related viruses were isolated after our previous study in 2009 or neglected by our previous study in 2009. Similarly, some other subordinate lineages, such as NS.1b1 was added in this report because the related viruses were isolated after our previous study in 2009.

### Phylogenetic analysis of representative sequences

**Fig. 33:**
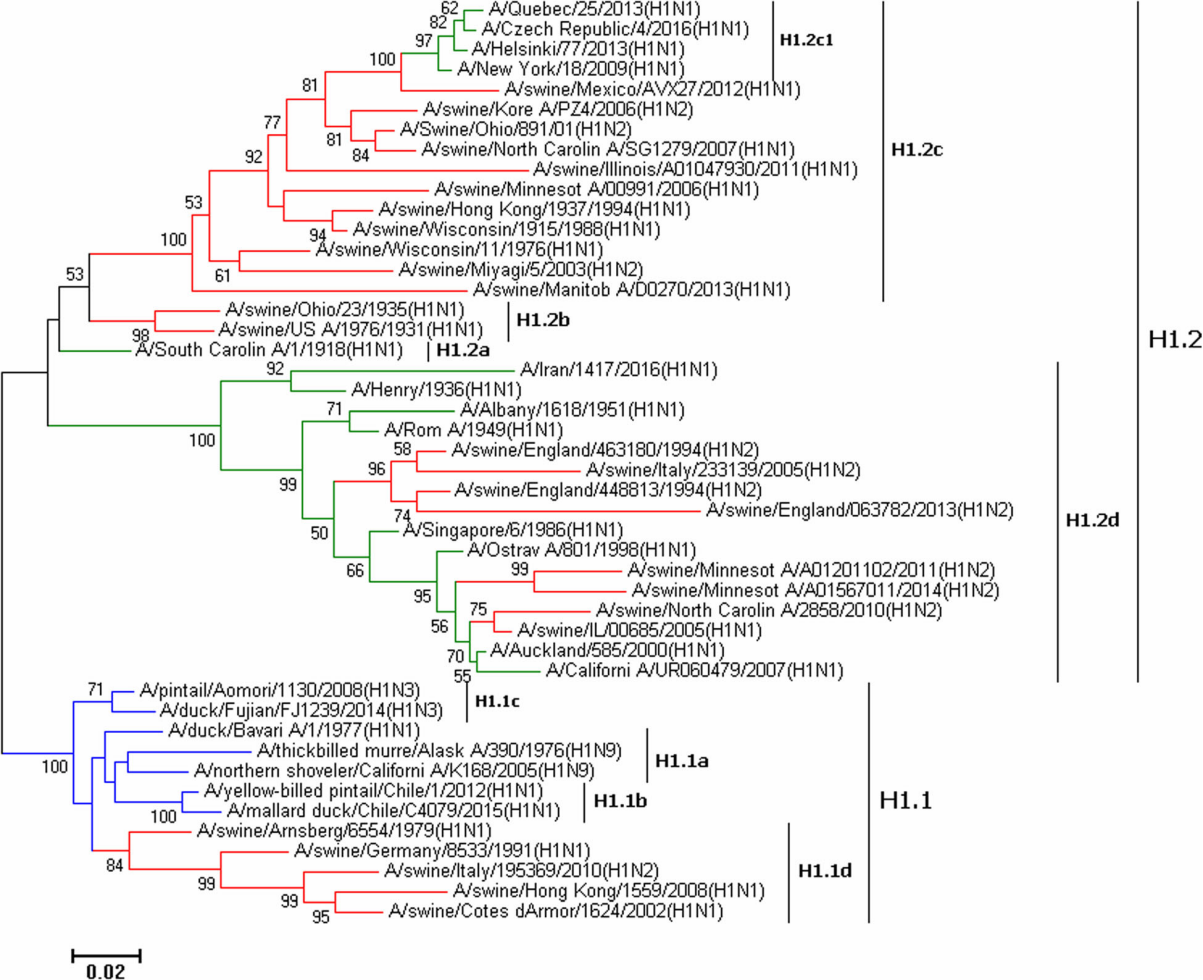
Phylogenetic tree of the viral HA gene representative sequences of H1 subtype. H1 subtype IAVs were classified into two primary lineages, H1.1 and H1.2, and each of them were further classified into four secondary lineages. Bootstrap values were given at relevant nodes

Phylogenetic relationships of representative sequences were calculated using the same software tool and the same parameters as the above sequences, and bootstraps values were calculated with 1000 replicates. The results suggested that most of the lineages and subordinate lineages designated in this report were supported by the bootstrap values calculated based on the representative sequences (Fig. 33 and Additional file 31: Figure S2_1, Additional file 32: Figure S2_2, Additional file 33: Figure S2_3, Additional file 34: Figure S2_4, Additional file 35: Figure S2_5, Additional file 36: Figure S2_6, Additional file 37: Figure S2_7, Additional file 38: Figure S2_8, Additional file 39: Figure S2_9, Additional file 40: Figure S2_10, Additional file 41: Figure S2_11, Additional file 42: Figure S2_12, Additional file 43: Figure S2_13, Additional file 44: Figure S2_14, Additional file 45: Figure S2_15, Additional file 46: Figure S2_16, Additional file 47: Figure S2_17 and Additional file 48: Figure S2_18). For example, as showed in Fig. 33, H1 subtype IAVs could be classified into two primary lineages, H1.1 and H1.2, and each of them were further classified into four secondary lineages, and six of the eight secondary lineages were of the support of bootstrap values > 70.

## Discussion

In order to calculate more accurately the diversity and distribution of IAVs, much more sequences were calculated in this study than in previous studies [17, 31–33]. This is also useful to demonstrate the panorama diversity and distribution of IAVs in a direct way. For example, more groups, clades or lineages of SIVs in H1 and H3 subtypes were demonstrated directly in this study than our previously studies which were based on selected representative sequences [31].

Beside our two panorama studies published in 2009, Shi et al. also reported a similar panorama analysis of the diversity and distribution of IAVs in 2010 based on the HA and NA genes, although Shi et al. did not analyze the diversity and distribution of IAVs based on the viral internal genes [33]. All these reports should be updated because multiple subtypes and lineages have emerged since 2010, and some sequences have been revised.

The diversity and distribution of IAVs were calculated and updated from a panorama view in this study. The results further confirmed some previous conclusions, such as that none of the HA gene of H3, H13, the NA gene of N2 subtypes of AIVs and the viral NS gene of AIVs could be simply classified into two lineages according to the Eastern and Western Hemispheres. The results also revised some previous conclusions, such as that some lineages designated previously (e.g. S1.7, S1.8, S2.7, S3.8, S5.9, S5.10, h3.3, and h5.3 [31, 32]) were deleted in this report because these lineages were designated previously with wrong sequences which have been revised thereafter. Meanwhile, some novel lineages, such as PB2.1b1, PB1.1f and H11.2b were added in this report mainly because these lineages did emerge in recent years.

This report also provided some novel suggestions on the diversity and distribution of SIVs. First, the SIVs in H1.2d should pose a considerable threat to humans in the future even though H1.2d has disappeared in humans for years. Second, SIVs were of more diversity than HuIVs during the same year if they were of the same HA primary lineage, and consequently we could not differentiate cross-species transmissions from accidental similarity through phylogenetic analysis. Third, it was rare for SIVs circulating in the Eastern Hemisphere to circulate in the Western Hemisphere, but not that rare for SIVs circulating in the Western Hemisphere to circulate in the Eastern Hemisphere.

This report also provided some novel suggestions on other respects of IAVs. First, it is not rare that IAVs from some avian lineages were isolated from humans, pigs or other mammalian hosts, but very rare that IAVs from any mammalian lineages were isolated from any birds. Second, isolated regions are favorable for forming some clades of IAVs because multiple clades or lineages of IAVs corresponded to some islands including Taiwan, Japan or Australia. Third, since multiple canine clades were classified through this report, and close contacts between humans and dogs are frequent, it is possible that dogs play a more important role in the ecology and evolution of IAVs than that we have imagined, and more surveillance of CIVs should be conducted to monitor the relevant risk [34, 35]. Fourth, it suggested that there are likely some other subtypes of IAVs in bats which have not been identified with the consideration of the diversity of known bat IAVs and the fact that bats usually do not move faraway. Fifth, most AIVs from Oceania belong to the lineages of the Eastern Hemisphere and most AIVs from South America belong to the lineages of the Western Hemisphere, but most AIVs from Oceania have formed specific subordinate lineages within the lineages of the Eastern Hemisphere, and most AIVs from South America have formed specific subordinate lineages within the lineages of the Western Hemisphere [33, 36–41]. Sixth, a few lineages of AIVs (H6.1a, H9.1, H13.1 and N2.1a) are distributed worldwide rather than restricted to the Western or Eastern Hemisphere, and this is consistent with a global pattern of AIV in wild birds proposed by Olsen et al. [42].

The panorama views provided by this report could act as a map for rapid identification of a strain or a group of IAVs is of a special significance. For example, the MP gene of two H3N8 subtype equine strains isolated in Japan in the 1970s ((A/equine/Sachiyama/1/1971(H3N8) and A/equine/Tokyo/2/1971(H3N8)) was located in the clade different from that of other H3N8 subtype equine strains isolated in the same decade, while the other genes of the two Japanese strains were located in the same clade with other H3N8 subtype equine strains isolated in the same decade (Additional file 3: Fig SI_3, Additional file 24: Fig S1_24 and Additional files 25-30: Figs S1_25–30), suggesting that the two Japanese strains were from gene reassortment with IAVs from other hosts.

This report identified some unexpected strains of IAVs. For example, A/Iran/187/2016, A/Iran/279/2016, A/Iran/1417/2016, and A/Shiraz/106/2015 were similar to the HuIVs isolated in the 1930s in lineage H1.2, and their sequences were indeed of high identity through nucleotide and protein BLAST analysis. This report also identified that seven genes excluding the PB2 gene of the swine strain A/swine/Yantai/16/2012(H9N2) were located in the same clades with the avian strain A/turkey/Wisconsin/1/1966(H9N2), and their sequences were indeed of high identity through nucleotide and protein BLAST analysis. In NP gene, one human secondary lineage (NP.2f) harbored one H7N9 subtype AIV (A/duck/Zhejiang/LS02/2014(H7N9)) and one H3N2 subtype SIV (A/swine/Jilin/19/2007(H3N2)), and their sequences were indeed of high identity through nucleotide and protein BLAST analysis. All of these special sequences were of no references and their origin and reliability need further confirmation.

Three influenza virus sequence databases have been available for this study. Among them, the NCBI FLU Database which we used for the analysis covers more sequences of the viral internal genes than the Influenza Research Database, and the Influenza Research Database covers more sequences of the viral HA and NA genes than the NCBI FLU Database, after collapsing the same protein sequences. The third database GISAID really covers more sequences than the NCBI FLU Database and the Influenza Research Database nearly by 30%, but GISAID could not support online collapsing the same protein sequences which is assumed to be important for random selection of the viral sequences for the analysis. Because we only used a part of randomly selected sequences rather than all the sequences covered by the NCBI FLU Database, to run the analysis, we may have not revealed the whole diversity and distribution of the viruses. We dowloaded 2722 H5 subtype of HA gene sequences and 1697 H7 subtype of HA gene sequences which are only available in GISAID, and phylogenetic analysis of these sequences fully supported our results in this study and no new groups or lineages were found for the addition of these sequences only available in GISAID (Additional files 49-50: Figs S1_31–32). This can be explained by the two facts: one is that the groups or lineages we classified are only the trunks rather than twigs of the relevant phylogenetic trees; the other is that we have used many randomly selected sequences for the analysis.

The analysis of this report was based on protein sequences rather than nucleotide sequences due to the need for simplification of calculation. Otherwise, so many nucleotide sequences should be beyond the capability of computers in laboratory. Also for the same reason the phylogenetic relationships were calculated using the neighbor-joining model rather than the maximum likelihood model. We have tested previously that the phylogenetic relationships calculated using the neighbor-joining model were quite similar to those calculated using the maximum likelihood model for IAVs [31]. The neighbor-joining model used in this report is of the advantage that the calculated phylogenetic tree is unique.

Analysis of the viral diversity and distribution based on protein sequences was reliable mainly because frame shift mutations are quite rare in IAVs [1]. Otherwise, the analysis should be questionable as one nucleotide substitution can lead to changes of multiple amino acid residues. We have tested 18,589 randomly selected sequences and no frame shift mutations were found (unpublished data).

The reliability of this paper was supported by the facts that the conclusions of this paper are largely consistent with many studies based on limited sequences [15–17, 21, 24, 33, 43, 44], and largely consistent with our previous panorama studies [31, 32]. Reasonable explanations were all given with the respect to the results different from our previous panorama studies (Additional file 51: Table S1). The reliability of this paper was further supported by the phylogenetic trees calculated using randomly selected representative sequences (Fig. 33 and Additional files 31-48: Figs S2_1-S2_18).

On the other side, many synonymous mutations occurring in IAVs could not be considered in our analysis based on protein sequences. Consequently, the internal genes of AIVs could not be classified into some lineages corresponding to the Western or Eastern Hemisphere, as could using nucleotide sequences in previous studies [32]. Nevertheless, this report did reveal the panorama views of AIVs, HuIVs, SIVs, EIVs, and CIVs successfully in a sketchy way, which are difficult to be revealed through other approaches.

Miscellaneous nomenclature systems have been reported targeting one specific gene, subtype or a small number of IAVs in previous studies [15, 16, 18–21, 43, 45–47], and some of these nomenclature systems are misleading. For example, some so-called “North American lineages” of SIVs or AIVs actually circulated widely in South America and/or Asia as well. Great efforts have been given to unify the nomenclature systems for IAVs, including the ones for designations of some lineages of H5 subtype HPAIVs (e.g. clade 2.3.4.4) [18, 19], for designations of HA and NA genes of IAVs (e.g. h9.4.2 or H2g2.3) [31, 33], and for designations of internal genes of IAVs (e.g. S8.6.3) [32], to facilitate communications on IAVs. In this report we further simplified the universal nomenclature system by alternately using numbers and letters. This simplified nomenclature system, if widely accepted, should simply communications on the ecology, evolution and epidemiology of IAVs among researchers.

An important issue regarding nomenclature of IAVs is that sometimes we should differentiate the HuIVs from a lineage of HuIVs and the HuIVs from a lineage of AIVs or SIVs caused by accidental cross-species transmission. We proposed here that, if needed, the HuIVs from a lineage of HuIVs be designated as HuIVs or adapted HuIVs, and the HuIVs from a lineage of AIVs or SIVs caused by accidental cross-species transmission be designated as unadapted HuIVs.

## Conclusions

In this study, 139,872 protein sequences available in GenBank with clear background were analyzed phylogenetically, and lineages and subordinate lineages were designated for each of the genes based on the updated panorama views using a novel universal nomenclature system. Phylogenetic trees of the two external viral genes (HA and NA) and six internal genes (PB2, PB1, PA, NP, MP, and NS) were constructed, and the diversity and the host, temporal and spatial distribution of all genes were calculated, statistically analyzed and updated. Various features regarding the diversity and distribution of IAVs were confirmed, revised or added through this study, as compared with previous reports. The updated panorama views of the diversity and distribution of IAVs and the relevant nomenclature system in this report is of high value for studies and communication of IAVs.

## Additional files

Additional file 1: **Figure SI_1.** The original phylogenetic tree of H1 subtype IAVs based on the HA gene sequences. (TIF 4507 kb)

Additional file 2: **Figure SI_2.** The original phylogenetic tree of H2 subtype IAVs based on the HA gene sequences. (TIF 95 kb)

Additional file 3: **Figure SI_3.** The original phylogenetic tree of H3 subtype IAVs based on the HA gene sequences. (TIF 5301 kb)

Additional file 4: **Figure SI_4.** The original phylogenetic tree of H4 subtype IAVs based on the HA gene sequences. (TIF 267 kb)

Additional file 5: **Figure SI_5.** The original phylogenetic tree of H7 and H15 subtypes of IAVs based on the HA gene sequences. (TIF 155 kb)

Additional file 6: **Figure SI_6.** The original phylogenetic tree of H9 subtype IAVs based on the HA gene sequences. (TIF 118 kb)

Additional file 7: **Figure SI_7.** The original phylogenetic tree of H5 subtype IAVs based on the HA gene sequences. (TIF 336 kb)

Additional file 8: **Figure SI_8.** The original phylogenetic tree of H6 subtype IAVs based on the HA gene sequences. (TIF 169 kb)

Additional file 9: **Figure SI_9.** The original phylogenetic tree of H8 subtype IAVs based on the HA gene sequences. (TIF 101 kb)

Additional file 10: **Figure SI_10.** The original phylogenetic tree of H10 subtype IAVs based on the HA gene sequences. (TIF 194 kb)

Additional file 11: **Figure SI_11.** The original phylogenetic tree of H11 subtype IAVs based on the HA gene sequences. (TIF 179 kb)

Additional file 12: **Figure SI_12.** The original phylogenetic tree of H12 subtype IAVs based on the HA gene sequences. (TIF 127 kb)

Additional file 13 **Figure SI_13.** The original phylogenetic tree of H14 subtype IAVs based on the HA gene sequences. (TIF 120 kb)

Additional file 14 **Figure SI_14.** The original phylogenetic tree of H16 subtype IAVs based on the HA gene sequences. (TIF 124 kb)

Additional file 15: **Figure SI_15.** The original phylogenetic tree of H13 subtype IAVs based on the HA gene sequences. (TIF 154 kb)

Additional file 16: **Figure SI_16.** The original phylogenetic tree of N1 subtype IAVs based on the NA gene sequences. (TIF 5681 kb)

Additional file 17: **Figure SI_17.** The original phylogenetic tree of N2 subtype IAVs based on the NA gene sequences. (TIF 2221 kb)

Additional file 18: **Figure SI_18.** The original phylogenetic tree of N4 subtype IAVs based on the NA gene sequences. (TIF 189 kb)

Additional file 19: **Figure SI_19.** The original phylogenetic tree of N5 subtype IAVs based on the NA gene sequences. (TIF 218 kb)

Additional file 20: **Figure SI_20.** The original phylogenetic tree of N6 subtype IAVs based on the NA gene sequences. (TIF 399 kb)

Additional file 21: **Figure SI_21.** The original phylogenetic tree of N3 subtype IAVs based on the NA gene sequences. (TIF 257 kb)

Additional file 22: **Figure SI_22.** The original phylogenetic tree of N9 subtype IAVs based on the NA gene sequences. (TIF 208 kb)

Additional file 23: **Figure SI_23.** The original phylogenetic tree of N7 subtype IAVs based on the NA gene sequences. (TIF 187 kb)

Additional file 24: **Figure SI_24.** The original phylogenetic tree of N8 subtype IAVs based on the NA gene sequences. (TIF 432 kb)

Additional file 25: **Figure SI_25.** The original phylogenetic tree of IAVs based on the PB2 gene sequences. (TIF 7104 kb)

Additional file 26: **Figure SI_26.** The original phylogenetic tree of IAVs based on the PB1 gene sequences. (TIF 6986 kb)

Additional file 27: **Figure SI_27.** The original phylogenetic tree of IAVs based on the PA gene sequences. (TIF 723 kb)

Additional file 28: **Figure SI_28.** The original phylogenetic tree of IAVs based on the NP gene sequences. (TIF 514 kb)

Additional file 29: **Figure SI_29.** The original phylogenetic tree of IAVs based on the MP gene sequences. (TIF 340 kb)

Additional file 30: **Figure SI_30.** The original phylogenetic tree of IAVs based on the NS gene sequences. (TIF 4262 kb)

Additional file 31: **Figure S2_1.** Phylogenetic tree of the viral HA gene representative sequences of H3 subtype. (TIF 57 kb)

Additional file 32: **Figure S2_2.** Phylogenetic tree of the viral HA gene representative sequences of H4 and H14 subtypes. (TIF 44 kb)

Additional file 33: **Figure S2_3.** Phylogenetic tree of the viral HA gene representative sequences of H2, H5 and H6 subtypes. (TIF 90 kb)

Additional file 34: **Figure S2_4.** Phylogenetic tree of the viral HA gene representative sequences of H7, H10 and H15 subtypes. (TIF 56 kb)

Additional file 35: **Figure S2_5.** Phylogenetic tree of the viral HA gene representative sequences of H8, H9 and H12 subtypes. (TIF 51 kb)

Additional file 36: **Figure S2_6.** Phylogenetic tree of the viral HA gene representative sequences of H11 subtype. (TIF 37 kb)

Additional file 37: **Figure S2_7.** Phylogenetic tree of the viral HA gene representative sequences of H13 and H16 subtypes. (TIF 39 kb)

Additional file 38: **Figure S2_8.** Phylogenetic tree of the viral NA gene representative sequences of N1 subtype. (TIF 58 kb)

Additional file 39: **Figure S2_9.** Phylogenetic tree of the viral NA gene representative sequences of N2 subtype. (TIF 63 kb)

Additional file 40: **Figure S2_10.** Phylogenetic tree of the viral NA gene representative sequences of N3 and N4 subtypes. (TIF 51 kb)

Additional file 41: **Figure S2_11.** Phylogenetic tree of the viral NA gene representative sequences of N5 and N8 subtypes. (TIF 51 kb)

Additional file 42: **Figure S2_12.** Phylogenetic tree of the viral NA gene representative sequences of N6, N7 and N9 subtypes. (TIF 51 kb)

Additional file 43: **Figure S2_13.** Phylogenetic tree of representative sequences of the viral PB2 gene sequences. (TIF 83 kb)

Additional file 44: **Figure S2_14**. Phylogenetic tree of representative sequences of the viral PB1 gene sequences. (TIF 101 kb)

Additional file 45: **Figure S2_15.** Phylogenetic tree of representative sequences of the viral PA gene sequences. (TIF 123 kb)

Additional file 46: **Figure S2_16.** Phylogenetic tree of representative sequences of the viral NP gene sequences. (TIF 101 kb)

Additional file 47: **Figure S2_17.** Phylogenetic tree of representative sequences of the viral MP gene sequences. (TIF 80 kb)

Additional file 48: **Figure S2_18.** Phylogenetic tree of representative sequences of the viral NS gene sequences. (TIF 95 kb)

Additional file 49: **Figure S1_31.** The original phylogenetic tree of H5 subtype IAVs based on the HA gene sequences in NCBI Flu database and 2722 sequences which are only available in GISAID. (TIF 475 kb)

Additional file 50: **Figure S1_32.** The original phylogenetic tree of H7 subtype IAVs based on the HA gene sequences in NCBI Flu database and 1697 sequences which are only available in GISAID. (TIF 273 kb)

Additional file 51: **Table S1.** Comparison of lineage classification with the two panorama reports published in 2009(references 31 and 32). (DOC 56 kb)

## Data Availability

The datasets used and/or analyzed during the current study are available from the corresponding author on reasonable request.

## Abbreviations

A(H1N1)pdm09: The pandemic H1N1 subtype HuIVs in 2009
A(H7N9)CN2013: The H7N9 subtype AIVs isolated in China which have caused many human infections and fatalities since the year 2013
AIVs: type A avian influenza viruses
CIVs: Canine influenza viruses (CIVs)
EIVs: Equine influenza viruses
HPAIVs: Highly pathogenic avian influenza viruses
HuIVs: Human influenza viruses
IAVs: Type A influenza viruses
SIVs: Swine influenza viruses (SIVs)
