# Supplementary figures and images for: Diversity and distribution of type A influenza viruses: an updated panorama analysis based on protein sequences
Source: Virol J. 2019 Jun 26;16:85. doi: 10.1186/s12985-019-1188-7 (PMC6595669; doi:10.1186/s12985-019-1188-7)

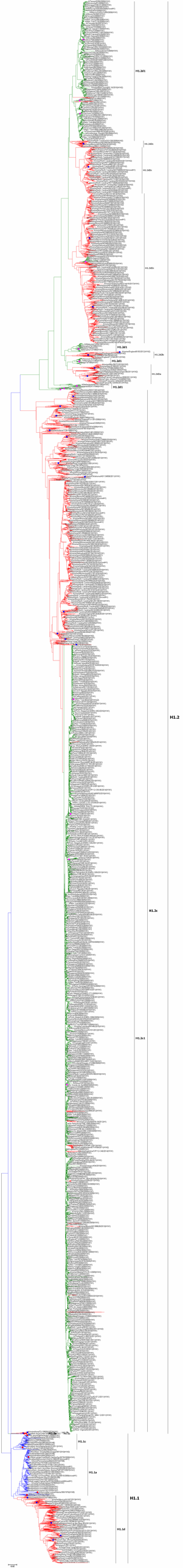

Supplement: Supplementary file 1 — Figure SI_1. The original phylogenetic tree of H1 subtype IAVs based on the HA gene sequences. (TIF 4507 kb) [file 12985_2019_1188_MOESM1_ESM.tif]

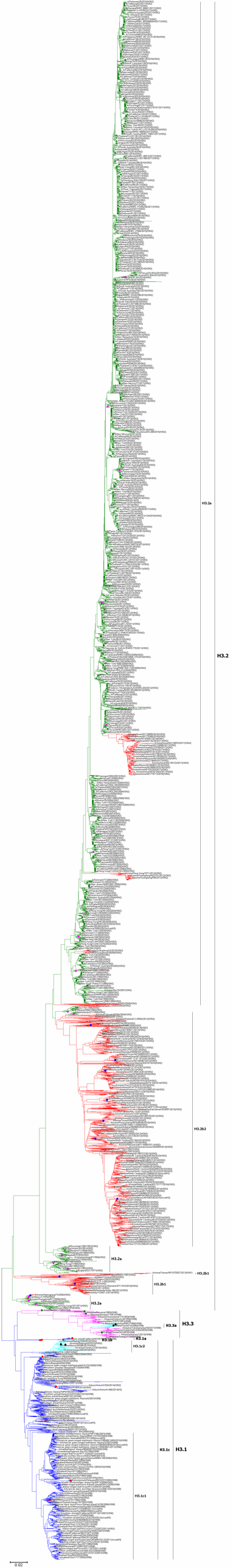

Supplement: Supplementary file 3 — Figure SI_3. The original phylogenetic tree of H3 subtype IAVs based on the HA gene sequences. (TIF 5301 kb) [file 12985_2019_1188_MOESM3_ESM.tif]

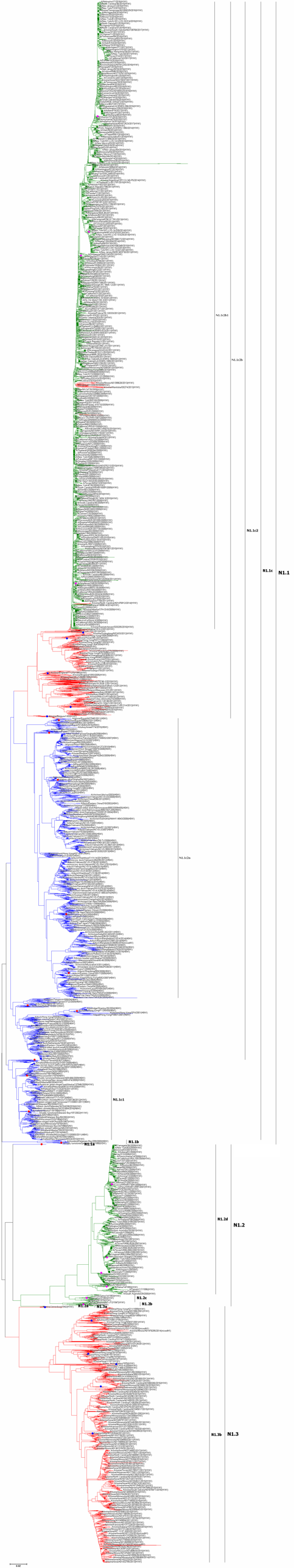

Supplement: Supplementary file 16 — Figure SI_16. The original phylogenetic tree of N1 subtype IAVs based on the NA gene sequences. (TIF 5681 kb) [file 12985_2019_1188_MOESM16_ESM.tif]

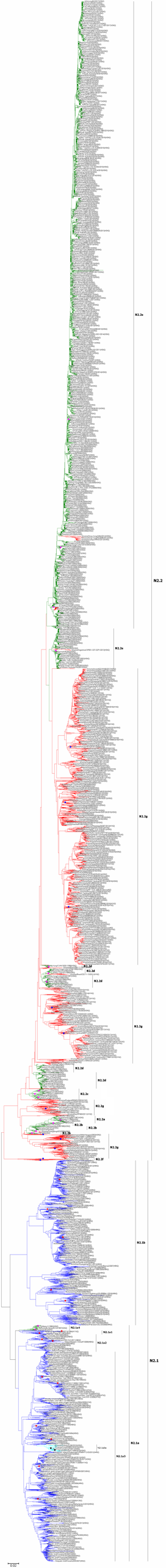

Supplement: Supplementary file 17 — Figure SI_17. The original phylogenetic tree of N2 subtype IAVs based on the NA gene sequences. (TIF 2221 kb) [file 12985_2019_1188_MOESM17_ESM.tif]

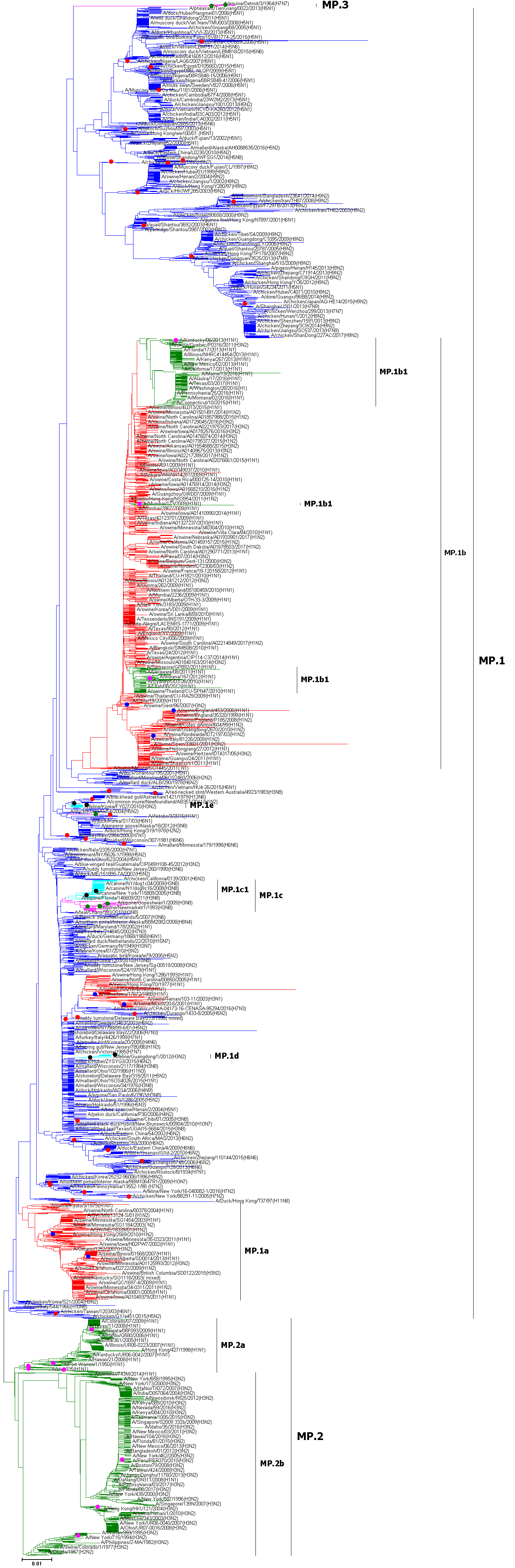

Supplement: Supplementary file 29 — Figure SI_29. The original phylogenetic tree of IAVs based on the MP gene sequences. (TIF 340 kb) [file 12985_2019_1188_MOESM29_ESM.tif]

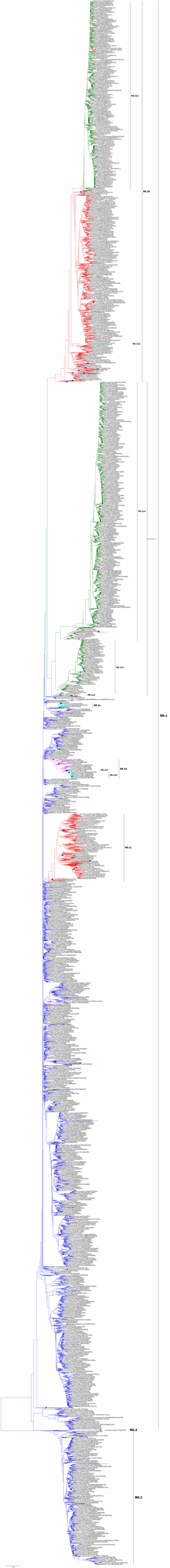

Supplement: Supplementary file 30 — Figure SI_30. The original phylogenetic tree of IAVs based on the NS gene sequences. (TIF 4262 kb) [file 12985_2019_1188_MOESM30_ESM.tif]

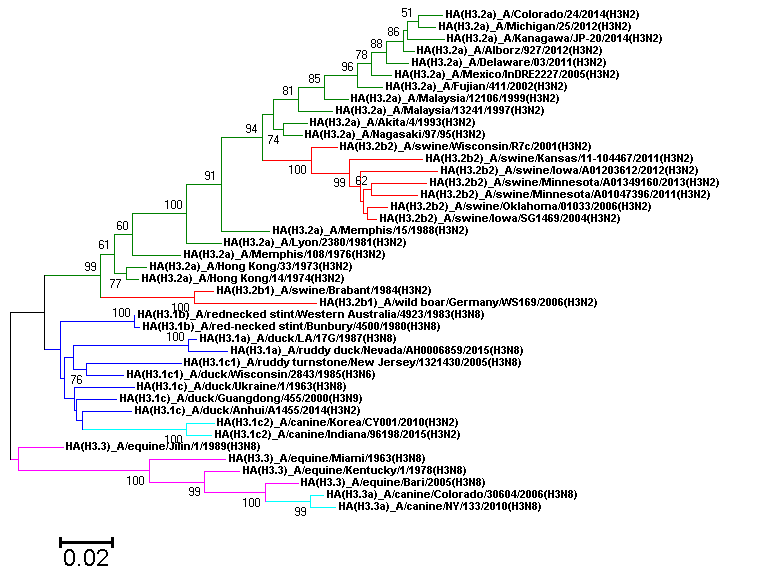

Supplement: Supplementary file 31 — Figure S2_1. Phylogenetic tree of the viral HA gene representative sequences of H3 subtype. (TIF 57 kb) [file 12985_2019_1188_MOESM31_ESM.tif]

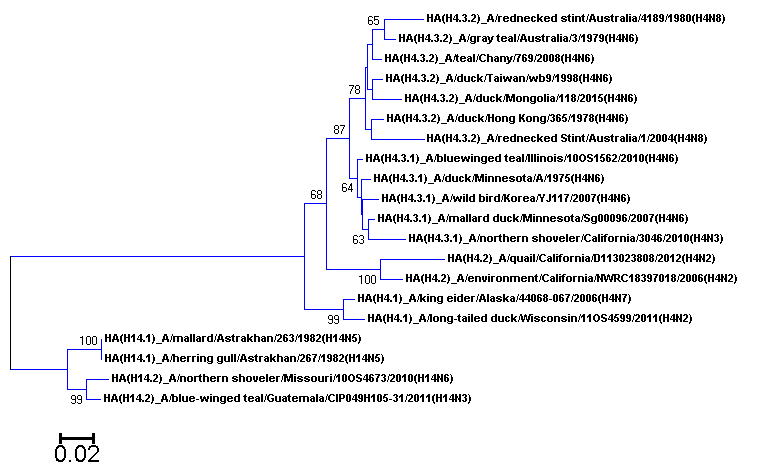

Supplement: Supplementary file 32 — Figure S2_2. Phylogenetic tree of the viral HA gene representative sequences of H4 and H14 subtypes. (TIF 44 kb) [file 12985_2019_1188_MOESM32_ESM.tif]

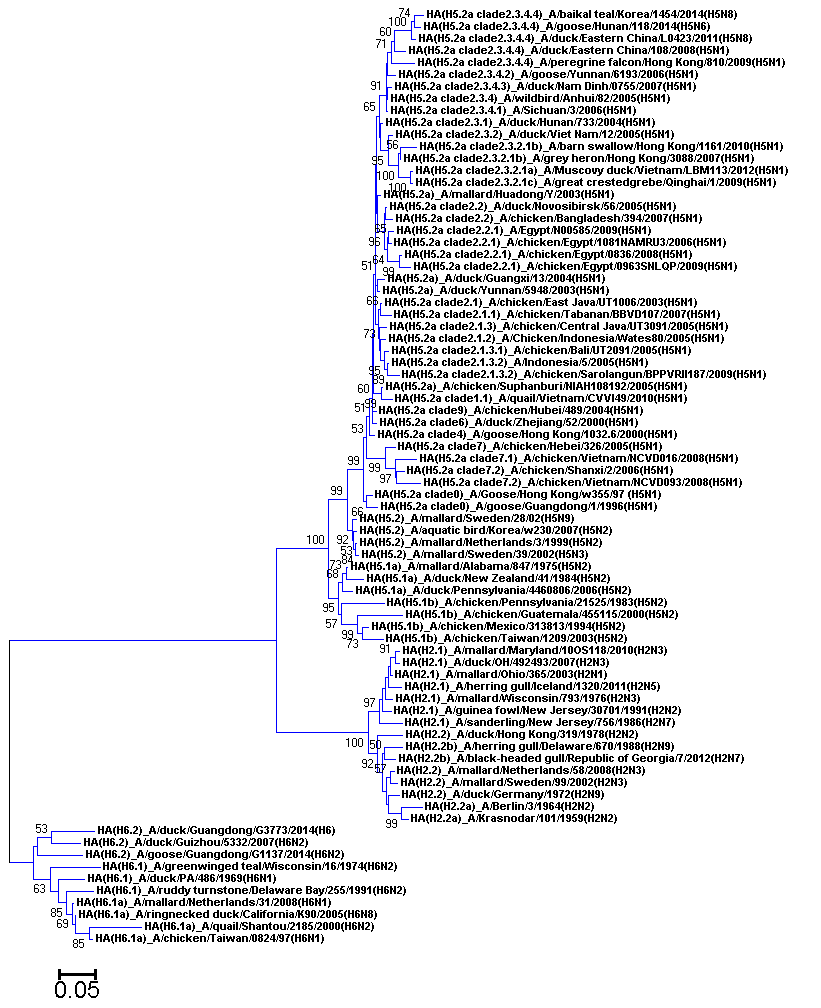

Supplement: Supplementary file 33 — Figure S2_3. Phylogenetic tree of the viral HA gene representative sequences of H2, H5 and H6 subtypes. (TIF 90 kb) [file 12985_2019_1188_MOESM33_ESM.tif]

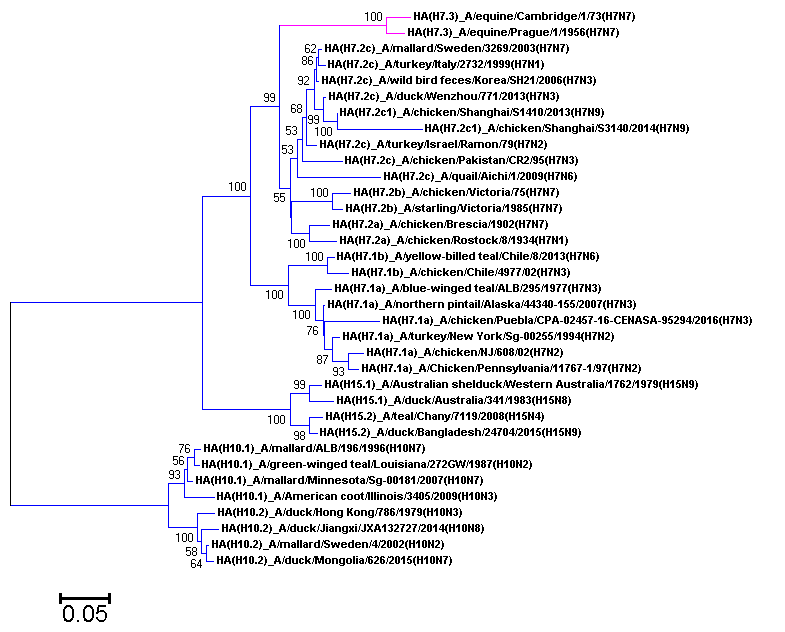

Supplement: Supplementary file 34 — Figure S2_4. Phylogenetic tree of the viral HA gene representative sequences of H7, H10 and H15 subtypes. (TIF 56 kb) [file 12985_2019_1188_MOESM34_ESM.tif]

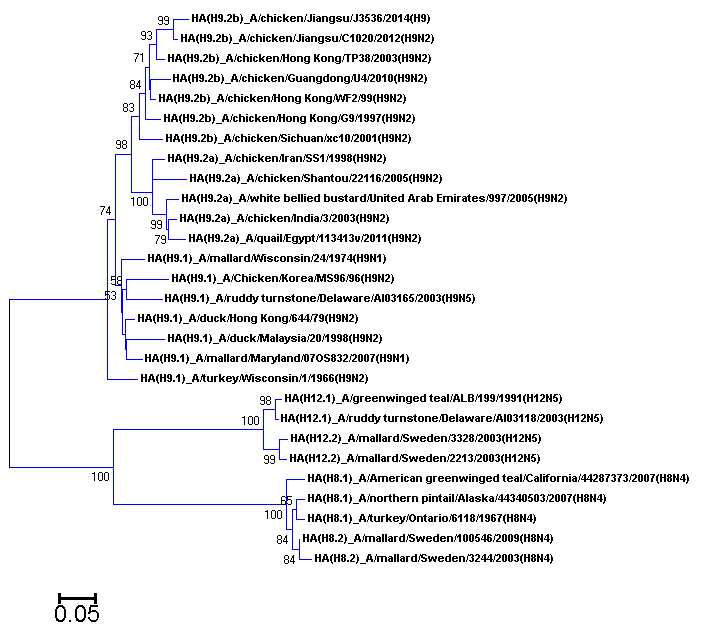

Supplement: Supplementary file 35 — Figure S2_5. Phylogenetic tree of the viral HA gene representative sequences of H8, H9 and H12 subtypes. (TIF 51 kb) [file 12985_2019_1188_MOESM35_ESM.tif]

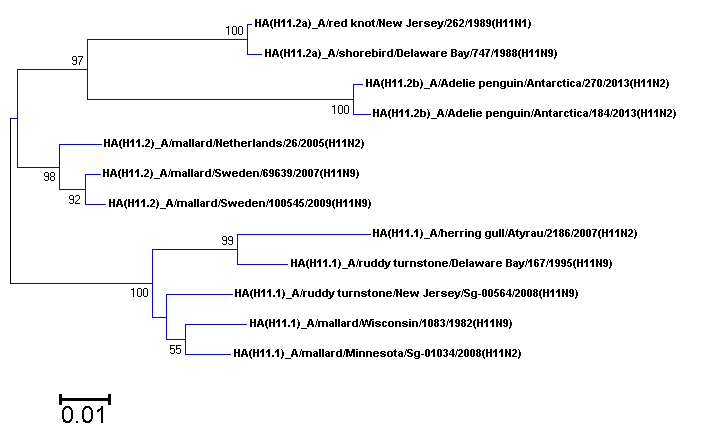

Supplement: Supplementary file 36 — Figure S2_6. Phylogenetic tree of the viral HA gene representative sequences of H11 subtype. (TIF 37 kb) [file 12985_2019_1188_MOESM36_ESM.tif]

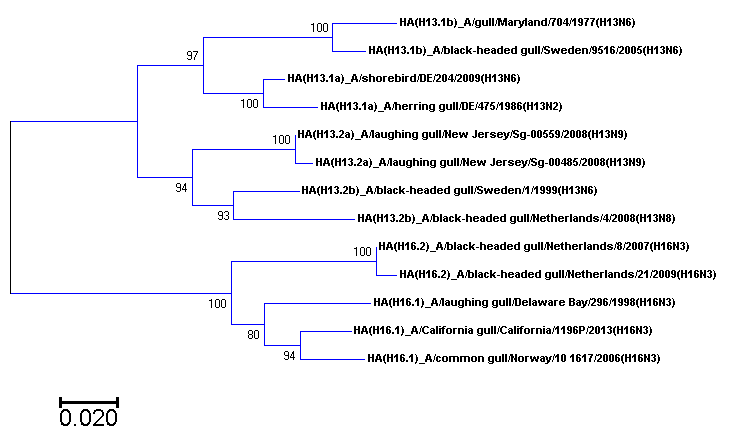

Supplement: Supplementary file 37 — Figure S2_7. Phylogenetic tree of the viral HA gene representative sequences of H13 and H16 subtypes. (TIF 39 kb) [file 12985_2019_1188_MOESM37_ESM.tif]

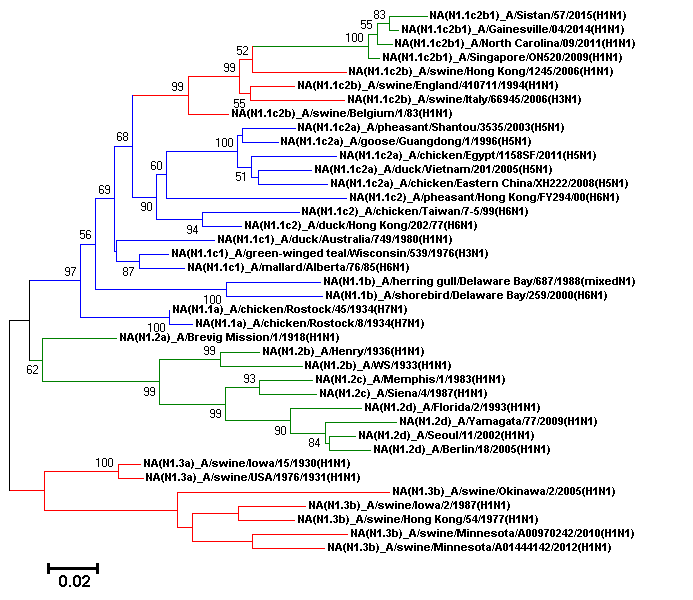

Supplement: Supplementary file 38 — Figure S2_8. Phylogenetic tree of the viral NA gene representative sequences of N1 subtype. (TIF 58 kb) [file 12985_2019_1188_MOESM38_ESM.tif]

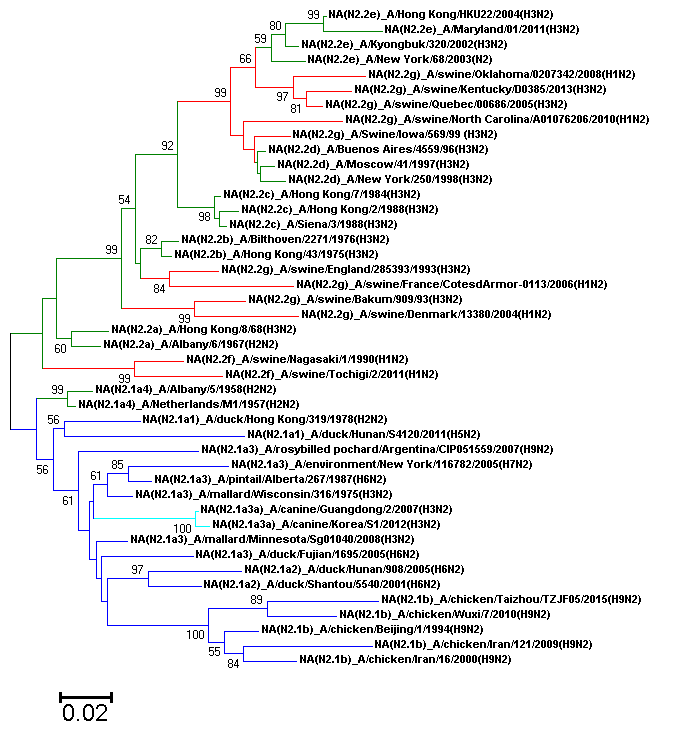

Supplement: Supplementary file 39 — Figure S2_9. Phylogenetic tree of the viral NA gene representative sequences of N2 subtype. (TIF 63 kb) [file 12985_2019_1188_MOESM39_ESM.tif]

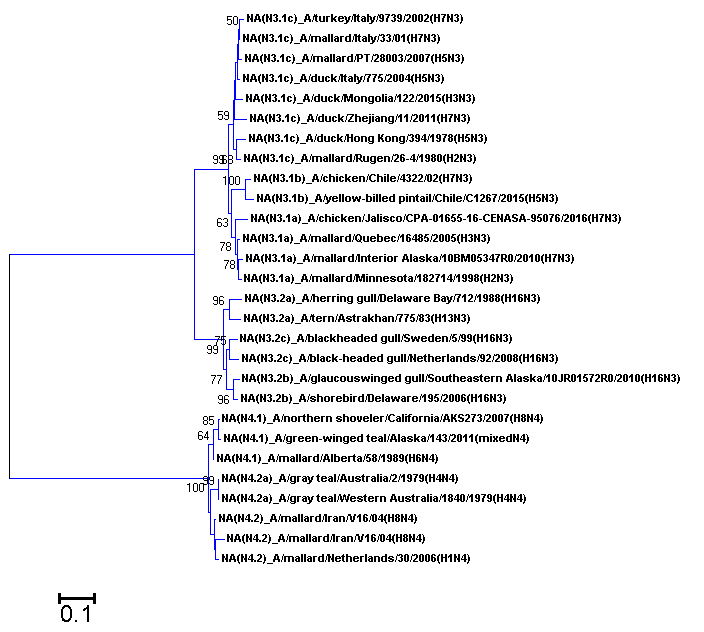

Supplement: Supplementary file 40 — Figure S2_10. Phylogenetic tree of the viral NA gene representative sequences of N3 and N4 subtypes. (TIF 51 kb) [file 12985_2019_1188_MOESM40_ESM.tif]

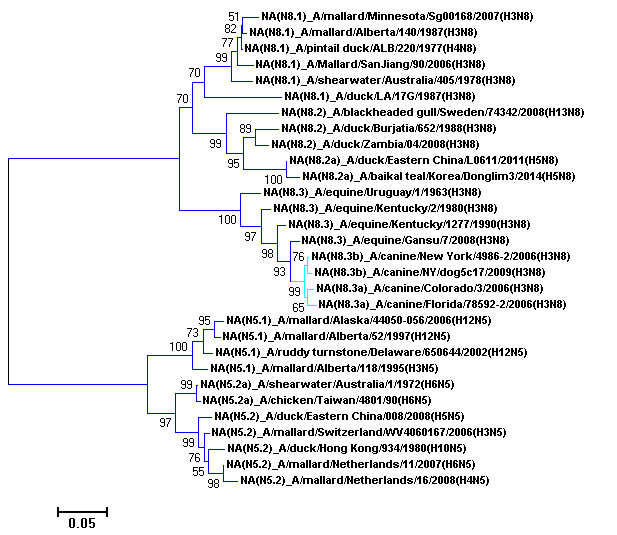

Supplement: Supplementary file 41 — Figure S2_11. Phylogenetic tree of the viral NA gene representative sequences of N5 and N8 subtypes. (TIF 51 kb) [file 12985_2019_1188_MOESM41_ESM.tif]

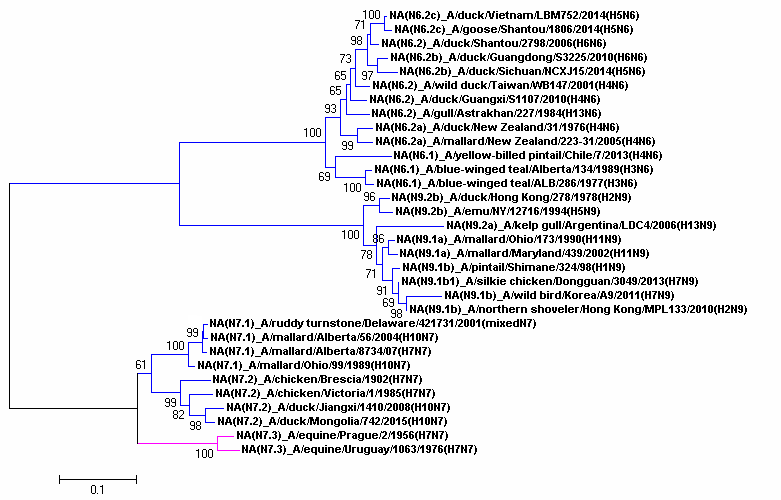

Supplement: Supplementary file 42 — Figure S2_12. Phylogenetic tree of the viral NA gene representative sequences of N6, N7 and N9 subtypes. (TIF 51 kb) [file 12985_2019_1188_MOESM42_ESM.tif]

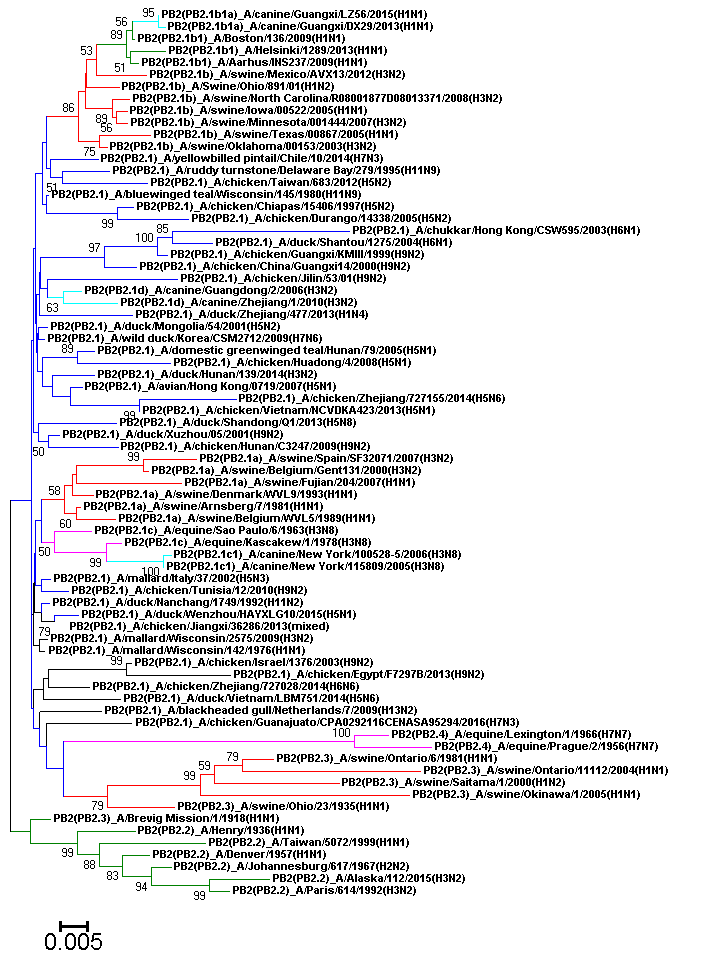

Supplement: Supplementary file 43 — Figure S2_13. Phylogenetic tree of representative sequences of the viral PB2 gene sequences. (TIF 83 kb) [file 12985_2019_1188_MOESM43_ESM.tif]

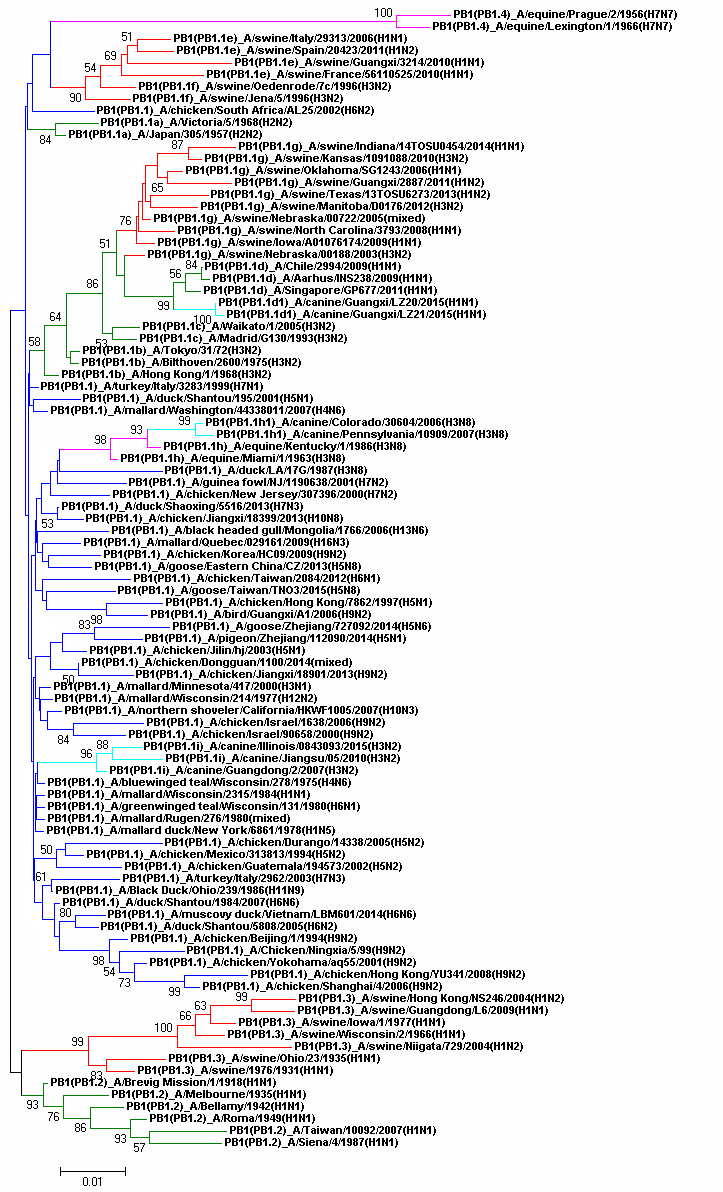

Supplement: Supplementary file 44 — Figure S2_14. Phylogenetic tree of representative sequences of the viral PB1 gene sequences. (TIF 101 kb) [file 12985_2019_1188_MOESM44_ESM.tif]

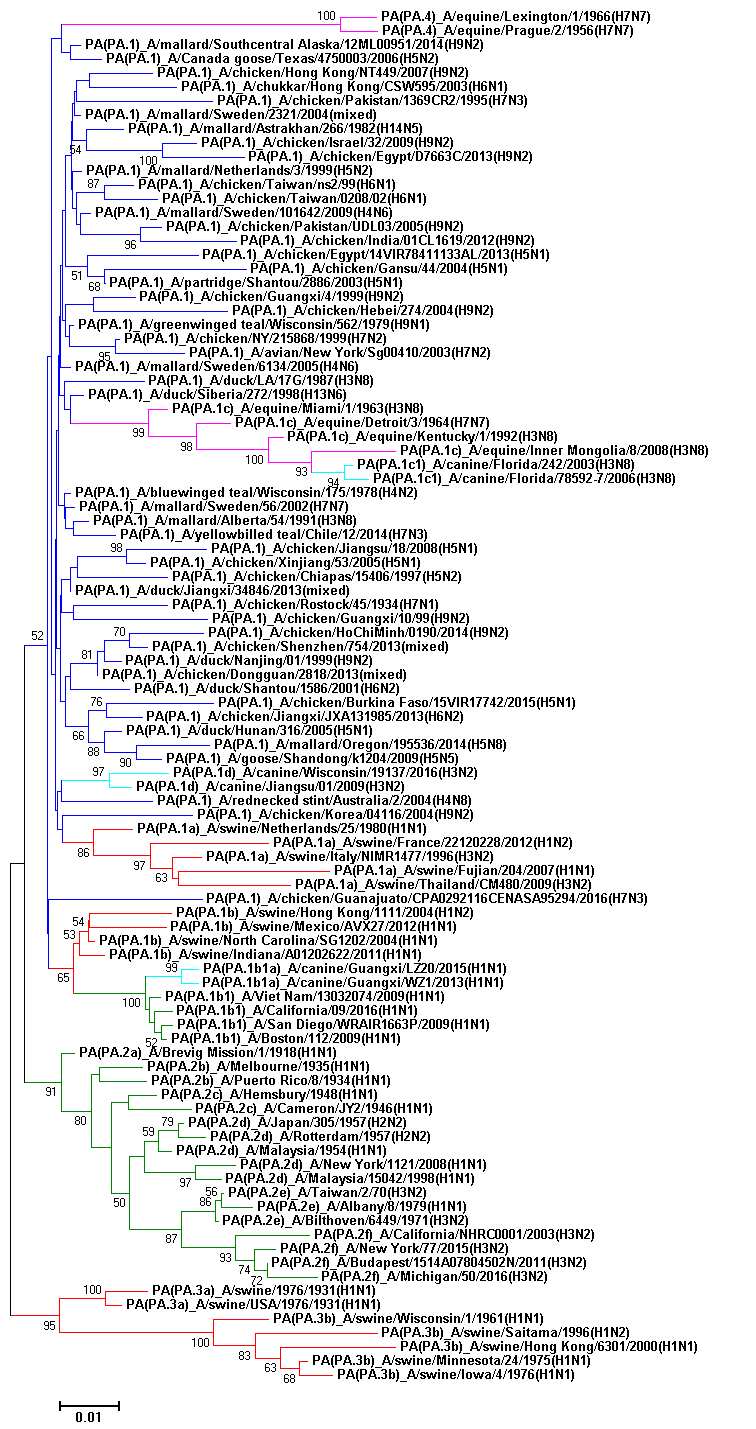

Supplement: Supplementary file 45 — Figure S2_15. Phylogenetic tree of representative sequences of the viral PA gene sequences. (TIF 123 kb) [file 12985_2019_1188_MOESM45_ESM.tif]

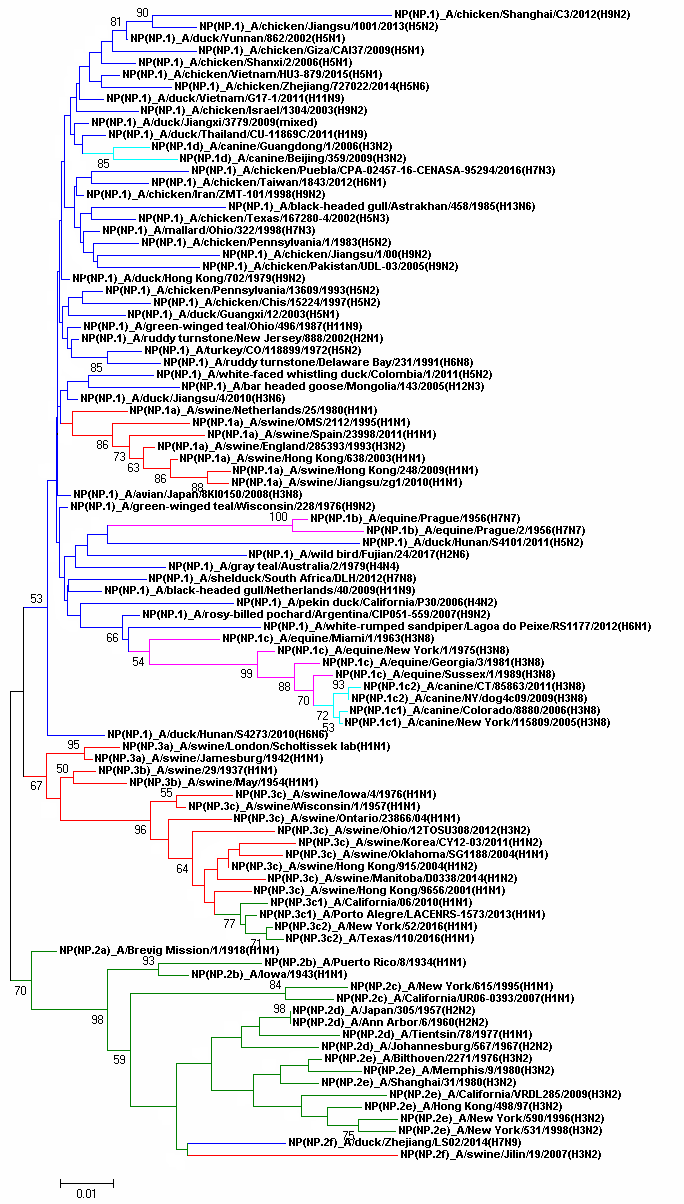

Supplement: Supplementary file 46 — Figure S2_16. Phylogenetic tree of representative sequences of the viral NP gene sequences. (TIF 101 kb) [file 12985_2019_1188_MOESM46_ESM.tif]

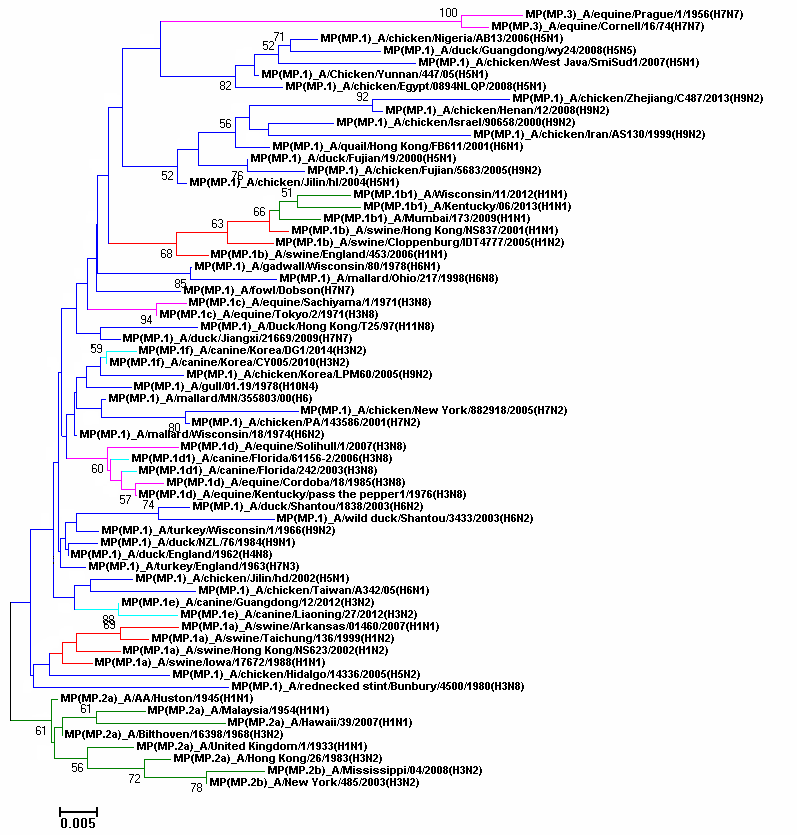

Supplement: Supplementary file 47 — Figure S2_17. Phylogenetic tree of representative sequences of the viral MP gene sequences. (TIF 80 kb) [file 12985_2019_1188_MOESM47_ESM.tif]

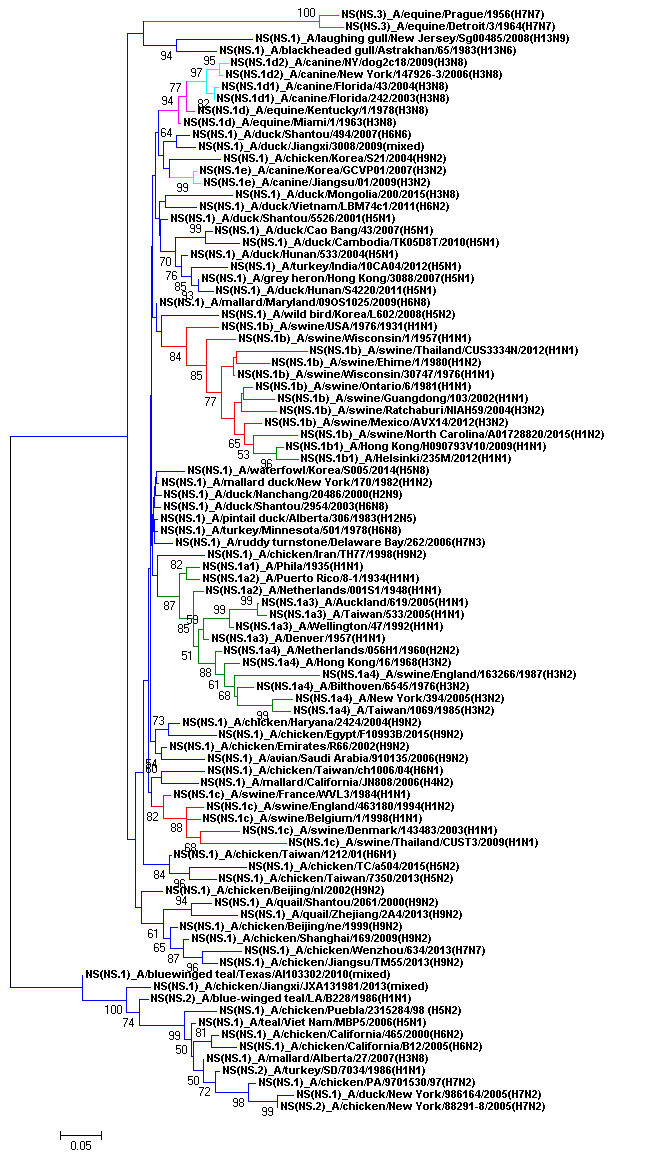

Supplement: Supplementary file 48 — Figure S2_18. Phylogenetic tree of representative sequences of the viral NS gene sequences. (TIF 95 kb) [file 12985_2019_1188_MOESM48_ESM.tif]

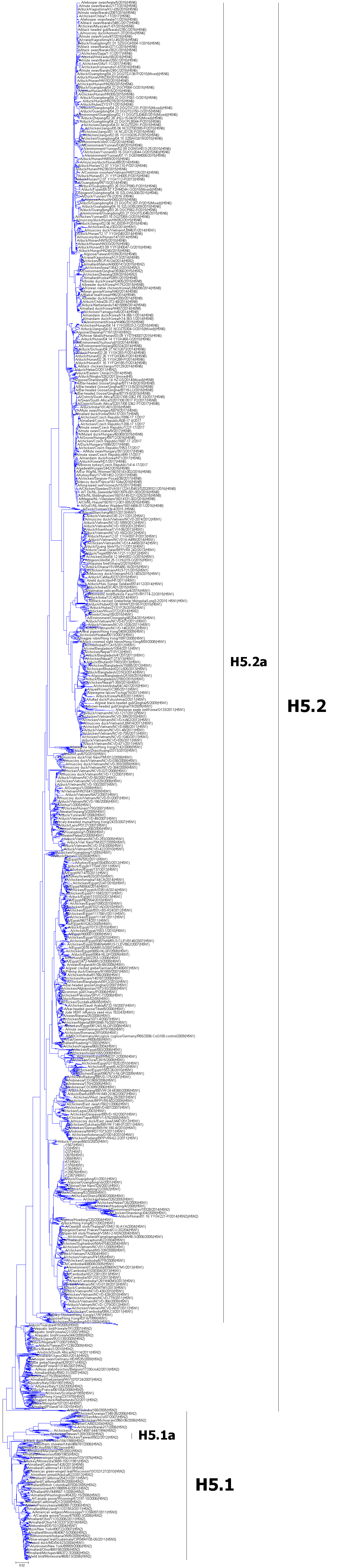

Supplement: Supplementary file 49 — Figure S1_31. The original phylogenetic tree of H5 subtype IAVs based on the HA gene sequences in NCBI Flu database and 2722 sequences which are only available in GISAID. (TIF 475 kb) [file 12985_2019_1188_MOESM49_ESM.tif]

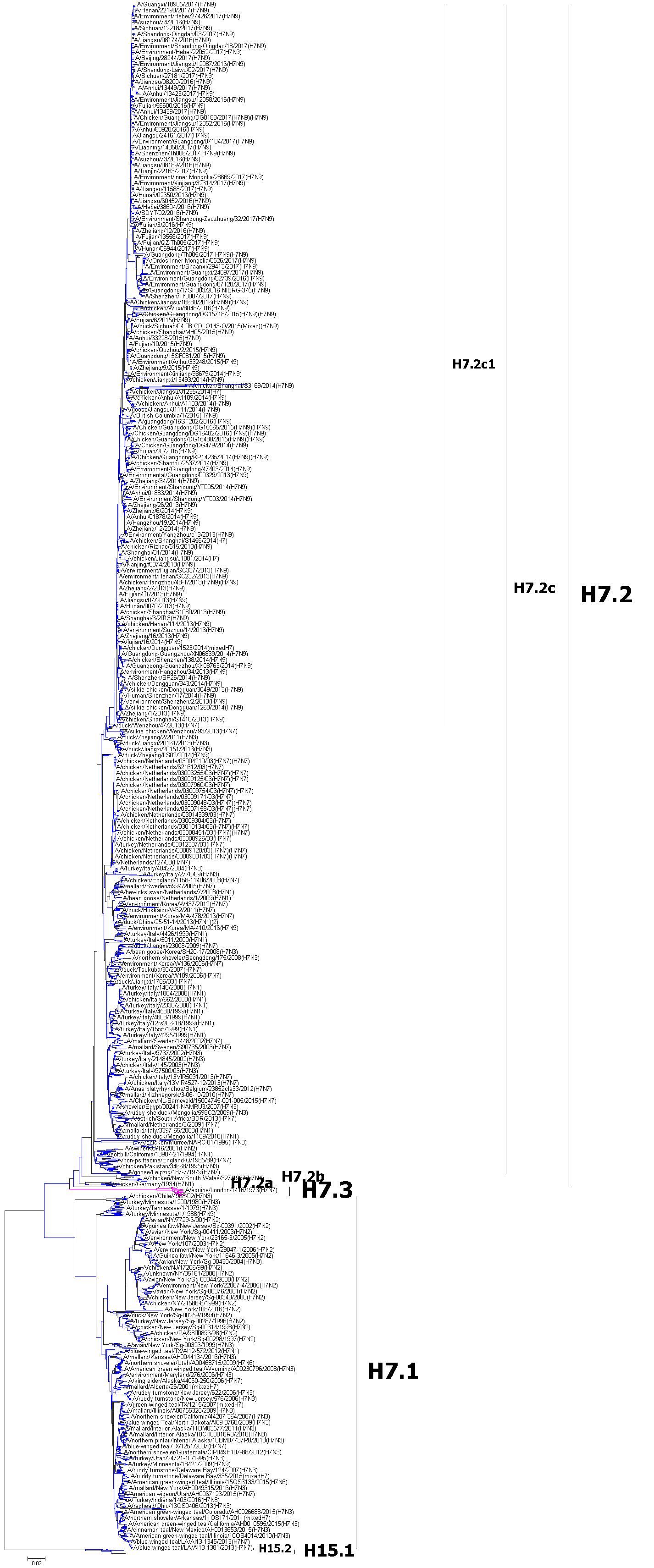

Supplement: Supplementary file 50 — Figure S1_32. The original phylogenetic tree of H7 subtype IAVs based on the HA gene sequences in NCBI Flu database and 1697 sequences which are only available in GISAID. (TIF 273 kb) [file 12985_2019_1188_MOESM50_ESM.tif]
