# Supplementary material for: Diversity and distribution of type A influenza viruses: an updated panorama analysis based on protein sequences
Source: Virol J. 2019 Jun 26;16:85. doi: 10.1186/s12985-019-1188-7 (PMC6595669; doi:10.1186/s12985-019-1188-7)
Supplement: Supplementary file 51 — Table S1. Comparison of lineage classification with the two panorama reports published in 2009(references 31 and 32). (DOC 56 kb) [file 12985_2019_1188_MOESM51_ESM.doc]

**Additional file 49: Table S1. Comparison of lineage classification with the two panorama reports published in 2009(references 31 and 32).**

| **Subtype** | **Primary lineage** | **Secondary lineage** | **Deletion of  primary lineages** | **Addition of lineages** |
| --- | --- | --- | --- | --- |
| **H1** | h1.1=H1.1; h1.2+h1.3=H1.2; h1.2=H1.2a+H1.2d; h1.3=H1.2b+H1.2c | h1.1.1=H1.1a; h1.1.2=H1.1c; h1.1.3=H1.1d h1.2.1=H1.2a; h1.2.2–h1.2.4=H1.2d1; h1.2.5=H1.2d2; h1.3.1=H1.2b; h1.3.2=H1.2c |  | H1.1b^g^, H1.2c1^d,g,i^ |
| **H2** | h2.1=H2.1; h2.2=H2.2 | h2.1.1+h2.2.2=H2.1; h2.2.6=H2.2a; h2.2.4=H2.2b |  |  |
| **H3** | h3.1=H3.1+H3.2; h3.2=H3.3; | h3.1.2=H3.1c; h3.1.1=H3.1c2; h3.1.3+h3.1.6=H3.2d1+H3.2d2; h3.2.1+h3.2.2=H3.3 | h3.3^a^ | H3.1a^g^, H3.1b^g^, H3.1c2^c^, H3.2b2^h^, H3.3a^c^ |
| **H4** | h4.1=H4.1+H4.2+H4.3a; h4.2=H4.3b |  |  | H4.1^h^, H4.2^h^ |
| **H5** | h5.1=H5.1; h5.2+h5.3=H5.2 | h5.2.3=H5.2a | h5.3^b^ | H5.1a^g,i^ |
| **H6** | h6.1=H6.1; h6.2=H6.2 |  |  | H6.1a^i^ |
| **H7** | h7.1=H7.1; h7.2=H7.2; h7.3=H7.3 | h7.1.1=H7.1a; h7.1.2=H1.1b; h7.2.2=H7.2a; h7.2.1=H7.2b; h7.2.3=H7.2c |  | H7.2c1^f,g,i^ |
| **H8** | No lineage |  |  | H8.1^e^ H8.2^e^ |
| **H9** | h9.1+h9.2+h9.3=H9.1; h9.4=H9.2 | h9.4.1=H9.2a; h9.4.2=H9.2b | h9.1^b^, h9.2^b^ |  |
| **H10** | h10.1=H10.1; h10.2=H10.2 |  |  |  |
| **H11** | h11.1=H11.1; h11.2=H11.2 |  |  | H11.2a^i^ H11.2b^g,i^ |
| **H12** | h12.1=H12.1; h12.2=H12.2 |  |  |  |
| **H13** | h13.1+h13.3=H13.1; h13.2=H13.2 | h13.1=H13.1a; h13.3=H13.1b; h13.2=H13.2a+H13.2b |  | H13.2a^e^, H13.2b^e^ |
| **H14** | No lineage |  |  | H14.1^e^, H14.2^e^ |
| **H15** | No lineage |  |  | H15.1^e^, H15.2^e^ |
| **H16** | No lineage |  |  | H16.1^e^, H16.2^e^ |
| **N1** | n1.1=N1.1; n1.2=N1.2; n1.3=N1.3 | n1.1.11=N1.1c2a; n1.1.1–n1.1.11=N1.1a–N1.1c; n1.2.1–n1.2.4=N1.2a–N1.2d; n1.3.1–n1.3.3=N1.3a+N1.3b |  | N1.1c2b1^d,g,i^ |
| **N2** | n2.1=N2.1; n2.2=N2.2 | n2.1.1–n2.1.7=N2.1a1–N2.1a4; n2.2.1–n2.2.4=N2.2a–N2.2g |  | N2.1a2^c^, N2.1a3a^c^ |
| **N3** | n3.1=N3.1; n3.2=N3.2 | n3.1.1=N3.1a; n3.1.2=N3.1b; n3.1.3=N3.1c; n3.2=N3.2a–N3.2c |  | N3.2a–N3.2c^e^ |
| **N4** | n4.1=N4.1; n4.2=N4.2 |  |  | N4.2a^i^ |
| **N5** | n5.1=N5.1; n5.2=N5.2 |  |  | N5.2a^i^ |
| **N6** | n6.1=N6.1; n6.2=N6.2 |  |  | N6.2a^e^,N6.2b^e^, N6.2c^e^ |
| **N7** | n7.1=N7.1; n7.2=N7.2; n7.3=N7.3 | n7.2.1–n7.2.3=N7.2 |  |  |
| **N8** | n8.1=N8.1; n8.2=N8.2; n8.3=N8.3 |  |  | N8.2a^i^,N8.3a^c^,N8.3b^1^ |
| **N9** | n9.1=N9.1; n9.2=N9.2 |  |  | N9.1b1^f,g,i^ |
| **PB2** | S1.1+S1.2+S1.7=PB2.1; S1.3=PB2.2; S1.4=PB2.3; S1.6=PB2.4; S1.5=PB2.1c | S1.1.4=PB2.1b; S1.1.5=PB2.1b1; S1.2.6=PB2.1a; S1.4.1+S1.4.2=PB2.3; S1.3.1–S1.3.4=PB2.2 | S1.7^b^ S1.8^a^ | PB2.1d^c^, PB2.1b1a^c^, PB2.1c1^c^,PB2.1b1^d,g,i^ |
| **PB1** | S2.1+S2.2+S2.6=PB1.1; S2.3=PB1.2; S2.4=PB1.3; S2.8=PB2.4; S2.5=PB1.1h | S2.1.5=PB1.1a; S2.1.6+S2.1.7=PB1.1b; S2.1.8=PB1.1c+PB1.1f; S2.1.9=PB1.1g; S2.1.10=PB1.1d; S2.2.6=PB1.1e | S2.7^a^ | PB1.1f^h^,PB1.1h1^c^, PB1.1i^c^,PB1.1d^d,g,i^ |
| **PA** | S3.1+S3.2+S3.6+S3.7=PA.1; S3.3=PA.2; S3.4=PA.3; S3.9=PA.4; S3.5=PA.1c | S3.2.6=PA.1a; S3.2.10=PA.1b; S3.2.11=PA.1b1; S3.4.1=PA.3a; S3.4.2=PA.3b; S3.3.1–S3.3.4=PA.2a–PA.2e | S3.8^a^ | PA.1b1^d,g,i^,PA.1b1a^c^,  PA.1c1^c^, PA.1d^c^ |
| **NP** | S5.1+S5.2+S5.5.1+S5.6+S5.7+S5.9=NP.1; S5.3=NP.2; S5.4=NP.3 | S5.2.3=NP.1a; S5.5.2=NP.1c; S5.3.1=NP.2a S5.3.2+S5.3.3=NP.2b; S5.3.4=NP.2c; S5.3.5=NP.2d; S5.3.6=NP.2e; S5.4.1=NP.3a+NP.3b; S5.4.2=NP.3c; S5.4.3=NP.3c1+NP.3c2 | S5.9^b^ S5.10^a^ | NP.1c1^c^, NP.1c2^c^, NP.1d^c^, NP.3c1^d,g,i^,NP.3c2^d,g,i^, NP.2f^h^ |
| **MP** | S7.1+S7.2=MP.1; S7.3=MP.2; S7.6=MP.3; S7.4=MP.1a; S7.5=MP.1c+MP.1d | S7.2.6=MP.1b; S7.2.7=MP.1b1–MP.1b3; S7.3.2+S7.3.4=MP.2a; S7.3.3+S7.3.5+S7.3.6=MP.2b |  | MP.1d1^c^, MP.1e^c^,MP.1f^c^, MP.1b1–MP.1b3^d,g,i^ |
| **NS** | S8.1+S8.2+S8.7+S8.8+S8.9=NS.1; S8.6=NS.2; S8.10=NS.3; S8.3=NS.1a; S8.4=NS.1b; S8.5=NS.1d | S8.2.2=NS.1c; S8.4.1–S8.4.3=NS.1b; S8.4.4=NS.1b1; S8.3.1=NS.1a1; S8.3.3+S8.3.4=NS.1a4+NS.1a5 |  | NS.1b2^c^,NS.1d1^c^, NS.1d2^c^, NS.1e^c^, NS.1b1^d,g,i^ |

Notes of the superscript:

a: Those lineages were deleted because the sequences have been revised greatly or the sequences might be wrong.

b: Those lineages were deleted because more nucleotide substitutions of the viruses within the lineages led to synonymous mutations.

c: Those lineages were added because the related viruses were isolated from dogs.

d: Those lineages were added the related viruses corresponded to A(H1N1)pdm09.

e: Those lineages were added because more novel sequences were available in GenBank after 2009.

f: Those lineages were added because the related viruses corresponded to the H7N9 subtype AIVs isolated in China since 2013.

g: Those lineages were added because the related viruses were isolated after our previous study in 2009.

h: Those lineages were added because the related viruses were neglected by our previous study in 2009.

i: Those lineages were added because of its distinct epidemiological significance rather than genetic distances.
